# One-pot synthesis of primary phosphines from white phosphorus†

**DOI:** 10.1039/d5cc03444b

**Published:** 2025-07-21

**Authors:** Michael Mende, Jose Cammarata, Daniel J. Scott, Robert Wolf

**Affiliations:** a Institute of Inorganic Chemistry, University of Regensburg 93040 Regensburg Germany robert.wolf@ur.de; b Department of Chemistry, University of Bath, Claverton Down Bath BA2 7AX UK ds2630@bath.ac.uk

## Abstract

Aryl and alkyl chlorides are inexpensive and readily accessible, making them ideal reagents for converting white phosphorus (P_4_) into primary phosphines RPH_2_. However, methods for achieving this transformation directly, bypassing undesired intermediates like PCl_3_, have remained elusive. This report describes the ‘one-pot’ synthesis of primary organophosphines from P_4_ using organotin compounds, including a multi-gram synthesis of PhPH_2_ featuring efficient recovery of organotin reagents for potential recycling.

## Abstract 2

‘One-pot’ synthesis of primary phosphines from P_4_ using organotin compounds and aryl or alkyl chlorides, including multi-gram synthesis of PhPH_2_.

## Graphical abstract

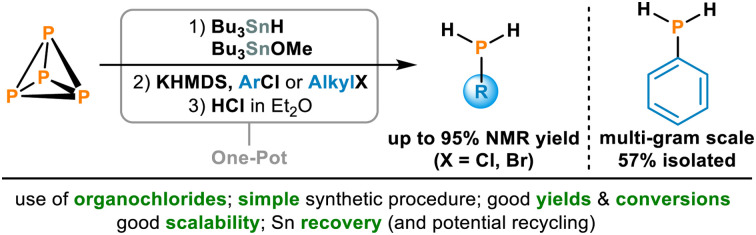

White phosphorus (P_4_) is a crucial industrial feedstock used for the generation of all commercially relevant organophosphorus compounds (OPCs). However, current industrial preparation of these P_1_ products relies on elaborate multistep processes, where P_4_ is typically oxidized using hazardous Cl_2_ gas to give PCl_3_, which must then be further functionalized towards the desired P_1_ products in separate steps with concomitant generation of chloride waste (Scheme 1a).^1,2^ As a result, a major goal for industry and academia is the development of improved, direct functionalisation of P_4_ towards useful P_1_ products avoiding hazardous intermediates and wasteful by-products.^3^ In recent years, several major breakthroughs in this field of chemistry have been reported.^4^

**Scheme 1:**
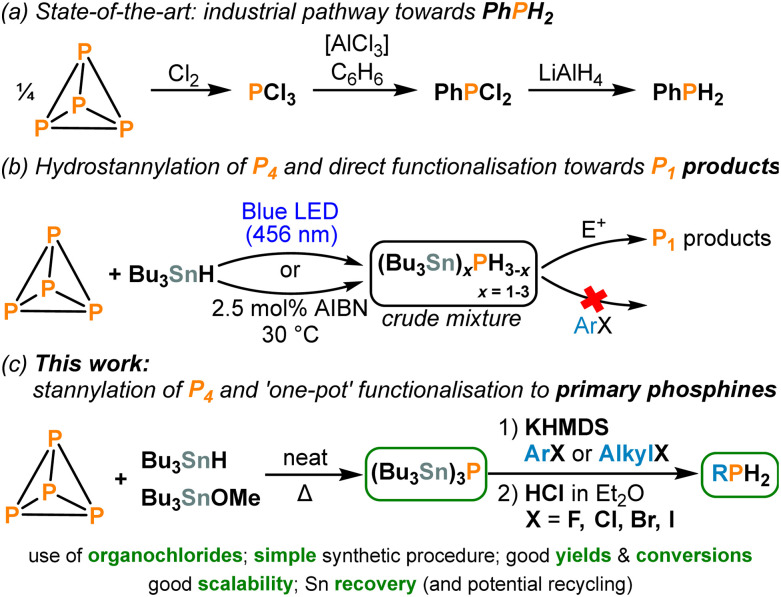
(a) Current industrial pathway for the synthesis of phenylphosphine (PhPH_2_). (b) Previously reported hydrostannylation of P_4_ followed by direct functionalisation using electrophiles (E^+^).^4*c*^ (c) This work: one-pot transformation of P_4_ into primary phosphines (RPH_2_; R = aryl or alkyl).

Phosphines are among the most important phosphorus compounds as they are ubiquitous in inorganic, organometallic and catalytic chemistry.^5^ Their versatility as ligands stems primarily from their ability to modify the steric and electronic properties of metal centres, which can be tailored by changing the substituents and the structure of the phosphine.^5*c*^ As the demand for new complexes and catalysts with unique characteristics continues to grow, there is an increasing need for more specialized phosphine ligands. Primary phosphines are key P_1_ intermediates through which the syntheses of many of these more exotic heteroleptic (and even *P*-stereogenic) phosphine ligands are achievable.^6^ Other important applications of primary phosphines can also be found throughout medicinal chemistry, polymer science, carbohydrate modification and macrocycle research.^7–10^

Recently, we demonstrated that hydrostannylation of P_4_, using the readily available radical reagent Bu_3_SnH, can successfully produce a hydrostannylphosphine mixture, leading to various desirable P_1_ compounds in a convenient ‘one-pot’ manner (see Scheme 1b).^4*c*,11^ However, several important limitations affected this reaction. Firstly, the functionalisation of the (Bu_3_Sn)_3−*x*_PH_*x*_ mixture (*x* = 0–3), which functions as an *in situ* “P^3−^” synthon, using aryl halides was not possible. The use of alkyl chlorides as electrophiles was also not feasible, even though these would be preferable precursors as they constitute the majority of cheap and commercially available organohalides.^12^ Indeed, using organochlorides in synthesis is often difficult in general due to their relatively strong C–Cl bonds.^13^ Secondly, the selective addition of only one electrophile per P atom was not achievable (*i.e.*, primary phosphines were not accessible).

Herein, we address both problems and present a straightforward, scalable method for generating primary phosphines from P_4_, using only inexpensive, readily available terminal reagents and abundant aryl/alkyl chlorides as the key electrophiles (Scheme 1c).

We have recently demonstrated that the phosphine (Bu_3_Sn)_3_P can be synthesized directly from P_4_.^4*c*^ This simplified alternative exhibits similar reactivity to the (Bu_3_Sn)_3−*x*_PH_*x*_ mixture, and we anticipated that this chemical simplicity would facilitate selective mono-functionalisation reactions.^14^ Thus, we began this project by further optimising this transformation. We were pleased to discover that (Bu_3_Sn)_3_P can be isolated in excellent yield at mmol scale (3.29 g, 91%) by simply heating the reagents (P_4_, Bu_3_SnH, Bu_3_SnOMe) in the absence of a solvent, without the need for auxiliary radical initiation (Scheme 2a; see also Section S2, ESI†).

**Scheme 2:**
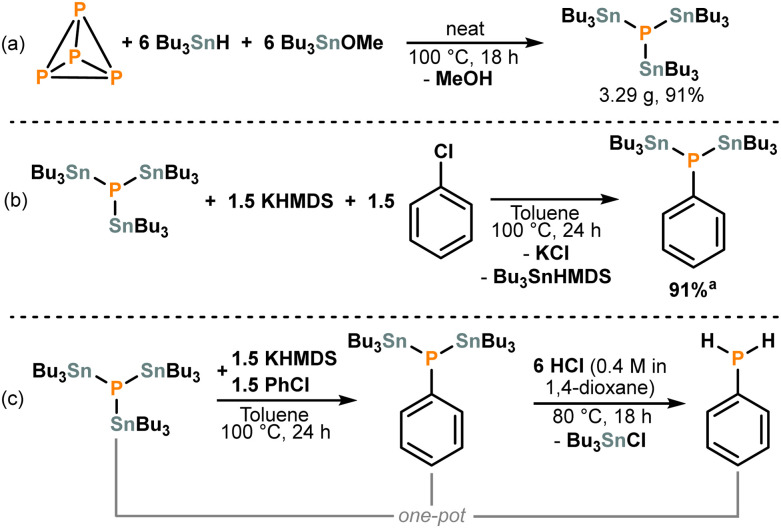
(a) Synthesis of (Bu_3_Sn)_3_P from P_4_ under neat conditions. (b) Optimised mono-arylation of (Bu_3_Sn)_3_P using PhCl. (c) One-pot transformation of (Bu_3_Sn)_3_P into PhPH_2_. ^*a*^ Spectroscopic yield.

The arylation of isolated (Bu_3_Sn)_3_P was then investigated. In an initial test reaction, combining (Bu_3_Sn)_3_P with three equivalents each of potassium bis(trimethylsilyl)amide (KHMDS) and chlorobenzene (PhCl) in toluene resulted in selective transformation into a single phosphorus-containing product with a chemical shift of −156.2 ppm in the ^31^P{^1^H} NMR spectrum, with 79% spectroscopic yield. ^117/119^Sn satellite analysis indicated the presence of two Sn atoms attached to phosphorus, confirming the selective monoarylation of (Bu_3_Sn)_3_P by PhCl to form (Bu_3_Sn)_2_PPh (see Section S3, ESI†).

Following this successful proof of principle, a range of other strong bases were screened as potential alternatives to KHMDS. However, with the sole exception of NaHMDS, which gave similar conversion (76%), all other replacements led to significantly reduced performance, with most resulting in complete loss of the desired reactivity (see Table S1, ESI†). In contrast, screening of other reaction parameters revealed that good conversion to the target product could be achieved without the need for excess base or electrophile (69% after 18 h), although use of small excesses was found to be beneficial, with 1.5 equivalents of both leading to an excellent spectroscopic yield of 91% after 24 h (Scheme 2b; see also Table S2, ESI†).

To complete the transformation of P_4_ into the parent primary phosphine, the two Bu_3_Sn moieties in (Bu_3_Sn)_2_PPh must be replaced with H atoms. Fortunately, we have previously shown that analogous exchange can be easily achieved by addition of HCl.^4*c*^ And indeed, addition of excess HCl (4 M in 1,4-dioxane) to the *in situ* generated (Bu_3_Sn)_2_PPh resulted in clean conversion into the parent phenylphosphine (PhPH_2_) as observed by ^31^P{^1^H} and ^31^P NMR spectroscopy (Scheme 2c; see also Section S4, ESI†).^15^

With each individual reaction step optimised, the one-pot transformation of P_4_ into PhPH_2_ was then targeted. To demonstrate scalability, as well as aid in product isolation, this was pursued at 80 mmol scale (2 × 40 mmol reactions, combined during workup). Thus, after neat reaction of P_4_ with Bu_3_SnH and Bu_3_SnOMe to afford (Bu_3_Sn)_3_P, toluene, KHMDS and PhCl were directly added, and the resulting solution heated overnight (to further transform to (Bu_3_Sn)_2_PPh). Following removal of volatiles, quenching with HCl in Et_2_O, and workup *via* filtration and fractional distillation, the target product PhPH_2_ could be successfully isolated as a clear, colourless liquid in good yield (20.0 g, 57%; Scheme 3a; see also Section S5, ESI†). This isolated material contains only traces of residual Sn (288 ppm by ICP-OES; Table S4, ESI†), which compares well with guidelines even for sensitive applications such as oral pharmaceuticals (up to 600 ppm).^16^ Moreover, the final fractional distillation also enabled recovery of the major Sn-containing byproduct, Bu_3_SnCl, in excellent yield (93%, 292.4 g). Since Bu_3_SnCl can easily be transformed back into Bu_3_SnH and Bu_3_SnOMe using very inexpensive reagents (*e.g.* NaBH_4_ or MeOH, respectively),^17^ this allows the formation of stoichiometric organotin waste to be bypassed, and creates a simple synthetic loop with very cheap terminal reagents (see Fig. S20, ESI†).

**Scheme 3:**
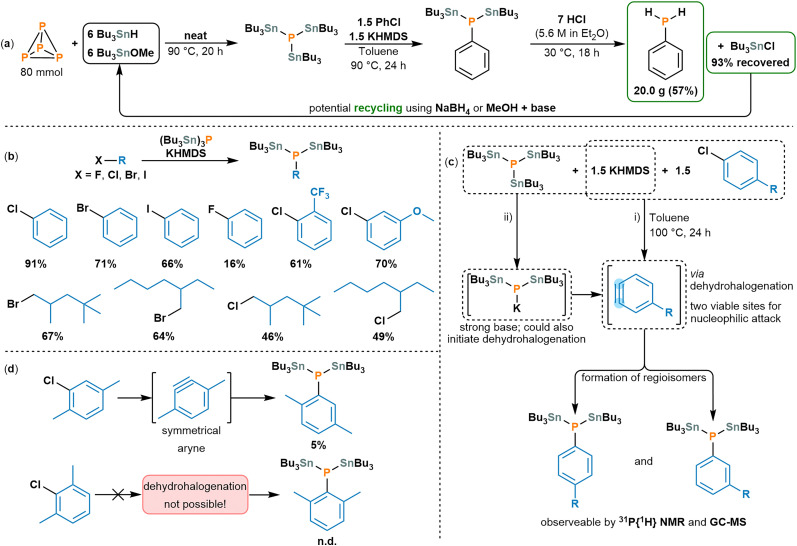
(a) One-pot synthesis and isolation of PhPH_2_ starting from P_4_, with recovery Bu_3_SnCl byproduct; (b) substrate scope of aryl/alkyl halides giving rise to products (Bu_3_Sn)_2_PAr or (Bu_3_Sn)_2_PAlk, respectively (for full reaction conditions, see Section S6, ESI†); (c) reaction of (Bu_3_Sn)_3_P (0.04 mmol), KHMDS, and substituted aryl chlorides and proposed intermediate aryne *via* dehydrohalogenation by KHMDS (i) or by the potassium phosphide KP(SnBu_3_)_2_ (ii); (d) mechanistic probe experiments using 2-chloro-*p*-xylene (top) and 2-chloro-*m*-xylene (bottom) (for full details, see Section S6, ESI†). Percentages in part (a) refer to isolated yields and in part (b) to spectroscopic yields determined using an internal standard (Ph_3_PO; for full details see Section S6, ESI†). Recovered yield in part (a) refers to Bu_3_Sn moieties used.

Having achieved a successful proof of concept for the parent primary aryl phosphine PhPH_2_, the generality of this transformation was then investigated by screening the reactivity of isolated (Bu_3_Sn)_3_P towards other aryl halides, as well as some alkyl derivatives. Interestingly, replacement of PhCl with the other parent halobenzenes led to inferior results, with conversion to (Bu_3_Sn)_2_PPh decreasing in the order Cl (91%) > Br (71%) > I (66%) ≫ F (16%). In contrast, excellent conversions to (Bu_3_Sn)_2_PAr could be achieved for certain other model ArCl substrates bearing both electron-donating and electron-withdrawing substituents (*m*-OMe and *o*-CF_3_, 70% and 61% respectively, Scheme 3b; for isomer identification, see Section S8, ESI†). Moreover, the aryl chloride substrates could be directly replaced with alkyl halides, leading to primary alkyl phosphines bearing industrially relevant alkyl motifs (2-ethylhexyl, 2,4,4-trimethylpentyl). In these cases, the bromides were found to slightly outperform the chlorides, although the latter still provided good spectroscopic conversions (64% *vs.* 49% for 2-ethylhexyl, 67% *vs.* 46% for 2,4,4-trimethylpentyl; Scheme 3b).

Alongside these successful substrates, several other alkyl halides were investigated, including secondary and tertiary substrates, but were found to give significantly inferior results (see Section S6, ESI†). Other aryl chlorides provided excellent conversion to (Bu_3_Sn)_2_PAr products; however, close inspection typically revealed two (or more) separate product signals in each of the ^31^P{^1^H} NMR reaction spectra, with similar chemical shifts and ^117/119^Sn satellites. This duplication was retained upon acidification with HCl, with ^31^P{^1^H} and ^31^P NMR spectra suggesting the formation of two isomeric primary aryl phosphines, which was further supported by GC-MS (Scheme 3c; for more detailed discussion, see Section S7.2, ESI†).

The observation of regioisomers was unexpected and suggests that the observed arylation reactions are unlikely to proceed *via* simple nucleophilic attack of (Bu_3_Sn)_3_P (or derivatives) on the electrophile, as was assumed for other electrophiles in our previous reports.^4*c*,14^ Indeed, S_N_Ar reactivity is unlikely on more fundamental grounds, since S_N_Ar is generally infeasible for Ar–Cl bonds in the absence of strongly electron-withdrawing substituents (and would be more favourable for Ar–F; *cf.* reduced reactivity of PhF *vs.* PhCl). Instead, we speculated that the reaction might proceed *via* an aryne intermediate. It is well known that aryl halides can form aryne intermediates upon dehydrohalogenation by strong bases, and this has been explicitly reported using various aryl halides and KHMDS.^18^ The aryne thus generated would feature two adjacent carbon atoms that are both viable sites for nucleophilic attack (Scheme 3c, pathway i).^19,20^ Unless the aryne is symmetrical, this should then lead to the formation of two regioisomeric products (at least in the absence of strong steric or electronic biases).

To test this proposal, two additional substrates were investigated: 2-chloro-1,3-dimethylbenzene and 2-chloro-1,4-dimethylbenzene. If the reaction does proceed *via* an aryne intermediate, this should lead to the generation of a single product for the former (as the aryne should be symmetrical) or no product for the latter (as aryne generation is precluded by the absence of an adjacent C–H bond). Both outcomes were confirmed experimentally by NMR spectroscopic analysis, albeit with low conversion in the former case, possibly due to steric hindrance (Scheme 3d; for further discussion see also Sections S6 and S7.2, ESI†).

Finally, the reaction between KHMDS and (Bu_3_Sn)_3_P in the absence of an organic substrate was also examined. ^31^P{^1^H} NMR monitoring showed the formation of a new, more upfield signal at −376.2 ppm with ^117/119^Sn satellites corresponding to two Sn atoms attached to the phosphorus. A similar upfield shift is observed during the known reaction of (Me_3_Si)_3_P and KO^*t*^Bu (another reaction between a tetrel-substituted phosphine and a strong, non-nucleophilic base) to generate the related potassium phosphide KP(SiMe_3_)_2_. As such, the new signal is assigned to the potassium phosphide KP(SnBu_3_)_2_ (see Section S7.1, ESI†).^21^ This suggests that rather than being deprotonated by KHMDS directly, aryne generation could also occur *via* initial formation of KP(SnBu_3_)_2_, which would also be a strong base (Scheme 3c, pathway ii).

In conclusion, we have developed a simple and efficient one-pot reaction for the direct generation of primary phosphines from P_4_*via* stannylation, using inexpensive and readily available terminal reagents. Focusing on the parent primary phosphine PhPH_2_, we have demonstrated the selectivity and scalability of the reaction, which avoids the chlorination of P_4_, including the efficient recovery of the key Bu_3_SnX reagents to minimize the formation of toxic waste. Several aryl chlorides substituted with electronically distinct functional groups also gave very good conversions and selectivity, as did several primary alkyl bromides and chlorides. We have also investigated the underlying reaction mechanism, which for aryl chlorides appears to proceed *via* dehydrohalogenation to an aryne and can therefore lead to two regioisomeric phosphine products. Further research on this topic is ongoing, particularly concerning the additional functionalisation of the intermediate products (Bu_3_Sn)_2_PR (R = aryl, alkyl), which still bear two Bu_3_Sn moieties. Their further *in situ* derivatization could therefore potentially yield heteroleptic and even chiral phosphines.

We thank Petra Lugauer for her valuable experimental assistance and Vanessa Tomanek for the ICP-OES measurement. We acknowledge the DFG (RW, WO 1496/12-1, project number 548830090) and EPSRC (DJS, EP/V056069/1) for their financial support, as well as the Alexander von Humboldt Foundation for awarding a postdoctoral fellowship to DJS.

## Conflicts of interest

A patent covering all the results described herein has been filed (as of 13 February 2020) by the University of Regensburg (EP 20,157,197.3; inventors, D. J. S. and R. W.). The authors declare no other competing interests.

## Supplementary Material

CC-061-D5CC03444B-s001

## Data Availability

The data supporting this article have been included as part of the ESI.†
