# Supplementary material for: One-pot synthesis of primary phosphines from white phosphorus
Source: Chem Commun (Camb). 2025 Jul 21;61(68):12721–4. doi: 10.1039/d5cc03444b (PMC12292815; doi:10.1039/d5cc03444b)
Supplement: CC-061-D5CC03444B-s001 [file CC-061-D5CC03444B-s001.pdf]

Supplementary Information (SI) for ChemComm.
This journal is © The Royal Society of Chemistry 2025

# Supplementary Information

# One-Pot Synthesis of Primary Phosphines from White Phosphorus

Michael Mende,[1] Jose Cammarata,[1] Daniel J. Scott*[2] and Robert Wolf*[1]

[1]Institute of Inorganic Chemistry, University of Regensburg, 93040 Regensburg, Germany

E-Mail: robert.wolf@ur.de

[2]Department of Chemistry, University of Bath, Claverton Down, Bath, BA2 7AY, UK

E-Mail: ds2630@bath.ac.uk

## Table of Contents

**S1. General information ... 2**

**S2. Synthesis and isolation of $(Bu_3Sn)_3P$ (1 mmol scale) ... 3**

**S3. Representative procedure and optimisation for the phenylation of $(Bu_3Sn)_3P$ using chlorobenzene (PhCl) towards (Bu3Sn)2PPh ... 6**

**S4. Representative procedure and optimisation for the protonation of $(Bu_3Sn)_2PPh$ to afford $PhPH_2$ ... 10**

**S5. 'One-pot' synthesis and isolation of $PhPH_2$ starting from $P_4$ *via* $(Bu_3Sn)_3P$ and $(Bu_3Sn)_2PPh$ (80 mmol scale) with $Bu_3SnCl$ recovery ... 14**

**S6. Characterization of reactions of $(Bu_3Sn)_3P$ and aryl/alkyl halides on 0.04 mmol scale ... 20**

**S7. Mechanistic investigations into the arylation of $(Bu_3Sn)_3P$ ... 39**

**S8. Structural investigation of reactions of $(Bu_3Sn)_3P$ and selected aryl chlorides ... 44**

**S9. References ... 51**

# S1. General information

All reactions and manipulations were performed under an $N_2$ atmosphere (< 0.1 ppm $O_2$, $H_2O$) through use of MBraun Unilab and GS MEGA Line gloveboxes, and standard Schlenk line techniques. All glassware was oven-dried (160 °C) overnight prior to use. Toluene, THF and diethyl ether were purified using an MBraun SPS-800 system and stored over molecular sieves (3 Å). MTBE was distilled from $CaH_2$ and stored over molecular sieves (3 Å). $C_6D_6$ was distilled from K and stored over molecular sieves (3 Å). $CDCl_3$ was degassed and stored over molecular sieves (3 Å). Tributyltin hydride and tributyltin methoxide were purchased from Thermo Scientific (both 97%) and degassed prior to use. KHMDS was purchased from Sigma-Aldrich (95%) and used as received. All other chemicals were also purchased from major suppliers; liquids were purified by Kugelrohr distillation and freeze-pump-thaw degassed three times prior to use; $P_4$ and $Ph_3PO$ were purified by sublimation; all others were used as received.

Qualitative NMR spectra were recorded at room temperature on Bruker Avance III HD 400 (400 MHz) or Bruker Avance *Neo* 500 (500 MHz) spectrometers and were processed using MestReNova 14.0. Chemical shifts, δ, are reported in parts per million (ppm); $^{1}H$ and $^{13}C$ shifts are reported relative to $SiMe_4$ and were calibrated internally to residual solvent peaks, while $^{31}P$ shifts and $^{119}Sn$ shifts were referenced externally to 85% $H_3PO_4$ (aq.) and $Me_4Sn$, respectively. NMR samples were prepared in the glovebox using NMR tubes fitted with screw caps. Optimization reactions towards $(Bu_3Sn)_2PPh$ and stannylation of $P_4$ to $(Bu_3Sn)_3P$ and subsequent functionalization to $P_1$ products were analysed by $^{31}P\{^{1}H\}$ spectra using triphenylphosphine oxide, $Ph_3PO$, as a subsequently added internal standard. Except where stated otherwise, integrals for $^{31}P\{^{1}H\}$ and $^{31}P$ spectra are provided for the purpose of qualitative comparison only and should not be considered quantitatively accurate. The abbreviations s, d, t, q, m are used to indicate singlet, doublets, triplets, quartets and multiplets, respectively.

Gas chromatography with mass-selective detector (GC-MS) was conducted with an Agilent 8860 GC system with mass detector 5977C. $H_2$ as carrier gas and a HP-5MS (30 m x 0.25 mm x 0.25 µm) column were used. The standard heating procedure was: 50 °C → 300 °C.

## S2. Synthesis and isolation of $(Bu_3Sn)_3P$ (1 mmol scale)

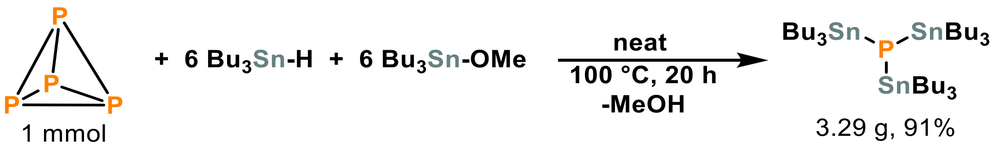


To a 100 mL Schlenk flask were added $P_4$ (123.9 mg, 1 mmol, 1 equiv.), $Bu_3SnH$ (1.61 mL, 6 mmol, 6 equiv.) and $Bu_3SnOMe$ (1.72 mL, 6 mmol, 6 equiv.). After stirring neat at 100 °C for 20 h volatiles were removed under vacuum. After distillation of the resulting oily liquid under high vacuum (*ca*. 100 °C, $10^{-5}$ mbar), $(Bu_3Sn)_3P$ was isolated as the remaining yellowish oil (3.29 g, 91%).

**$^{1}$H NMR** (400 MHz, 300 K, $C_6D_6$) δ[ppm] = 1.83-1.63 (2H, m), 1.51-1.40 (2H, m), 1.32-1.14 (2H, m), 0.98 (3H, t, $^{3}J(^{1}H-^{1}H)$ = 7.4 Hz).

**$^{31}$P{$^{1}$H} NMR** (162 MHz, 300 K, $C_6D_6$) δ[ppm] = –346.5 (s).

**$^{31}$P NMR** (162 MHz, 300 K, $C_6D_6$) δ[ppm] = –346.5 (s).

**$^{119}$Sn{$^{1}$H} NMR** (121 MHz, 300 K, $C_6D_6$) δ[ppm] = 37.6 (d, $^{1}J(^{31}P-^{119}Sn)$ = 911 Hz, $^{2}J(^{119}Sn-^{117}Sn)$ = 277 Hz).

**$^{13}$C{$^{1}$H} NMR** (101 MHz, 300 K, $C_6D_6$) δ[ppm] = 29.6 (d, $J(^{31}P-^{1}H)$ = 1.4 Hz), 27.6 (s), 14.8 (d, $J(^{31}P-^{1}H)$ = 3.7 Hz), 13.5 (s).

NMR data are consistent with previous reports.[1]

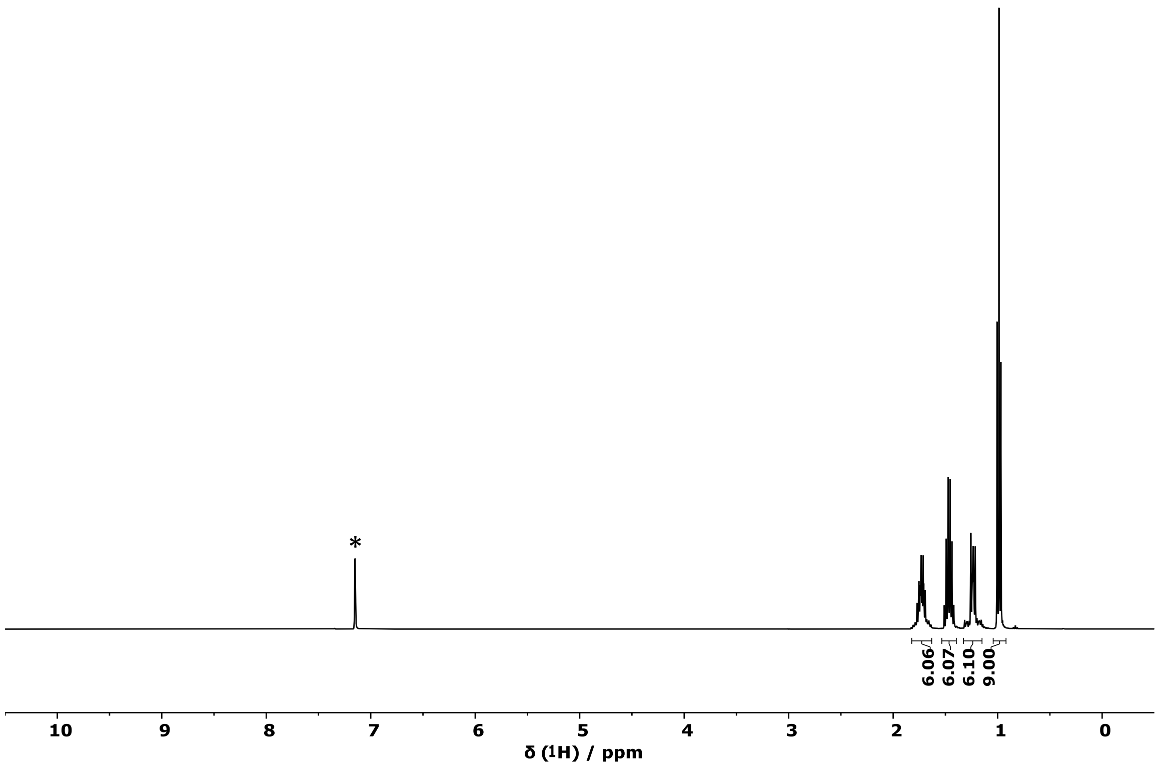


**Figure S1**: $^{1}H$ NMR spectrum of $(Bu_3Sn)_3P$ in $C_6D_6$ (*).

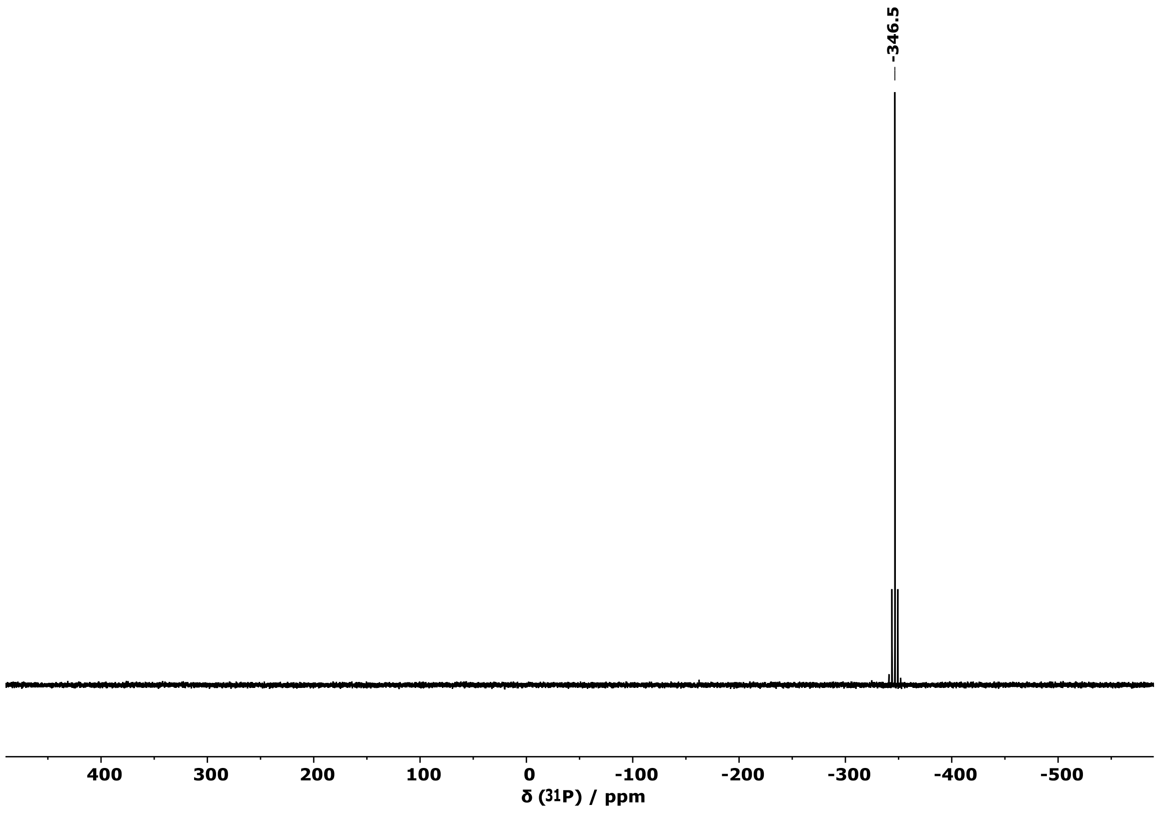


**Figure S2**: $^{31}P\{^{1}H\}$ NMR spectrum of $(Bu_3Sn)_3P$ in $C_6D_6$.

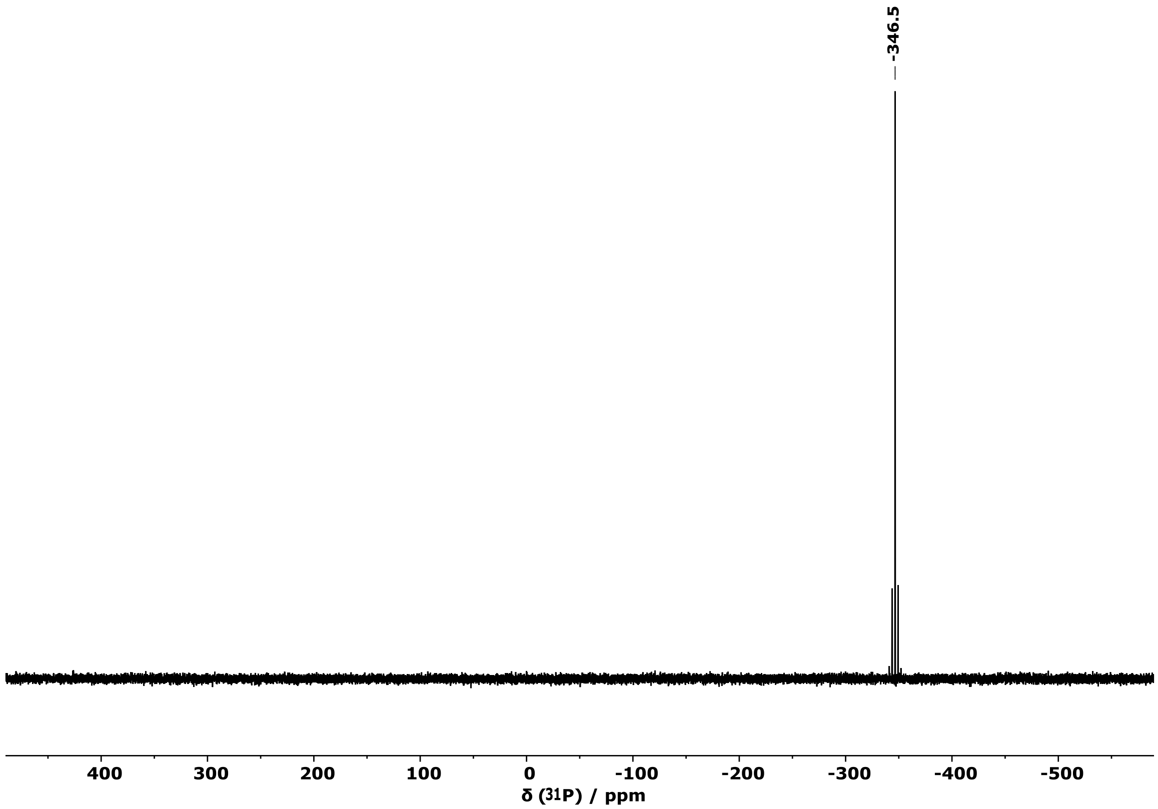


**Figure S3**: $^{31}P$ NMR spectrum of $(Bu_3Sn)_3P$ in $C_6D_6$.

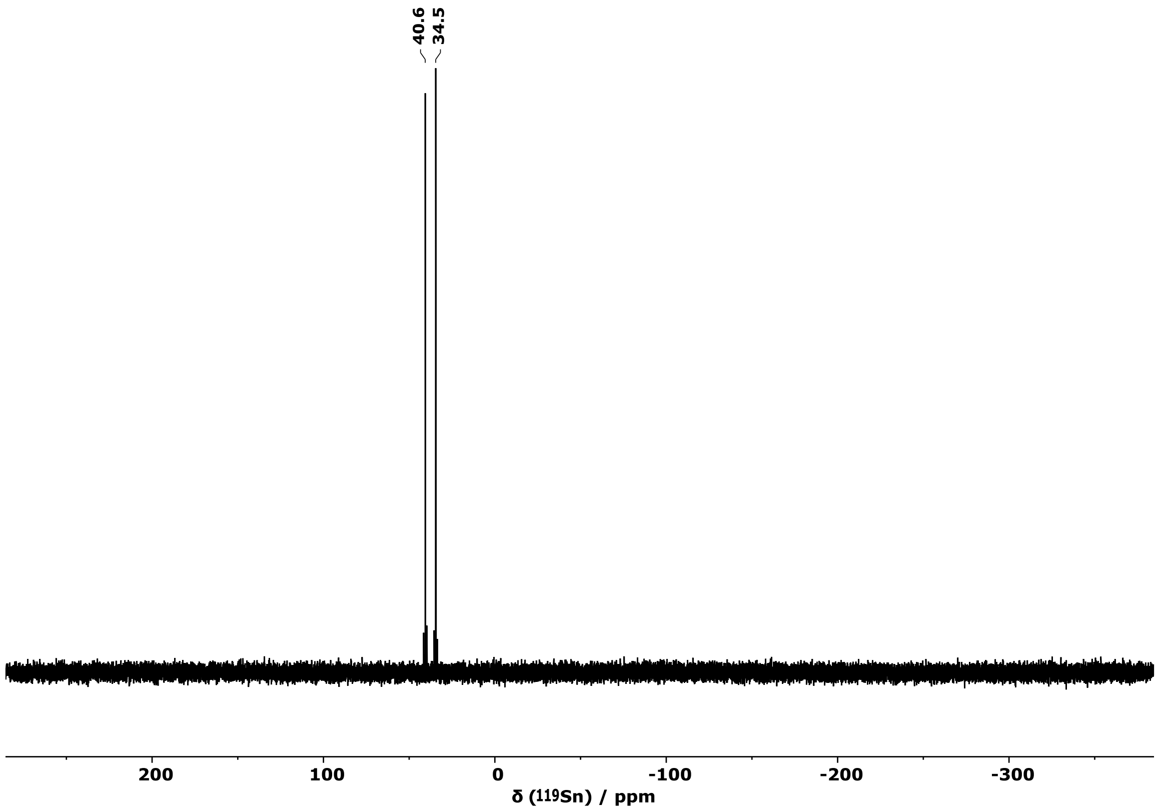


**Figure S4**: $^{119}Sn\{^1H\}$ NMR spectrum of $(Bu_3Sn)_3P$ in $C_6D_6$.

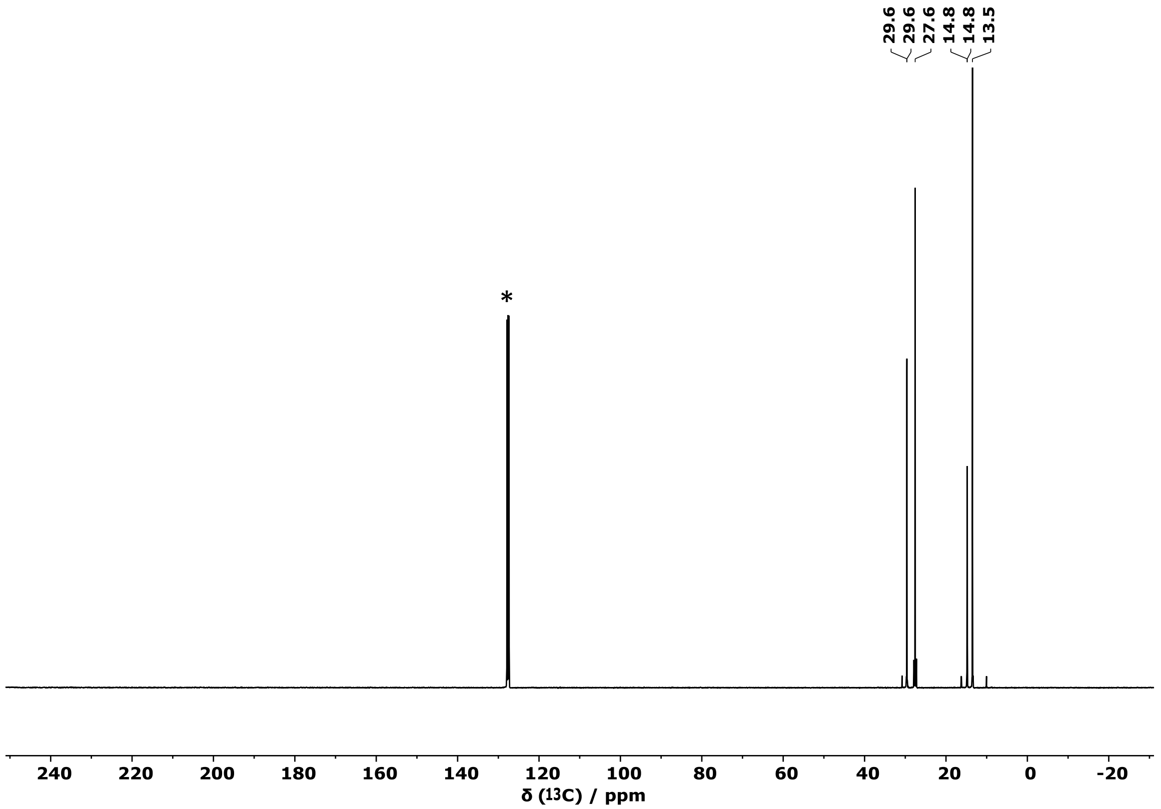


**Figure S5**: $^{13}C\{^1H\}$ NMR spectrum of $(Bu_3Sn)_3P$ in $C_6D_6$ (*).

## S3. Representative procedure and optimisation for the phenylation of $(Bu_3Sn)_3P$ using chlorobenzene (PhCl) towards (Bu3Sn)2PPh

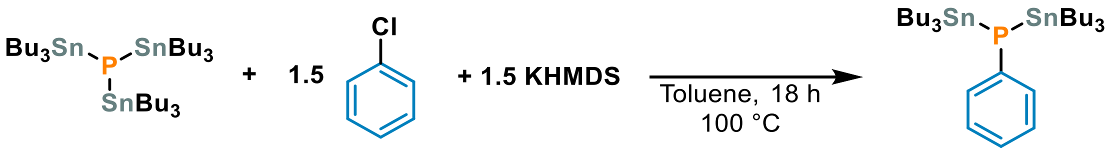


To a 10 mL Schlenk flask were added $(Bu_3Sn)_3P$ (0.04 mmol, 36.0 mg, 1 equiv.), PhCl (0.06 mmol, 6.1 µL, 1.5 equiv.) and KHMDS (0.06 mmol, 11.9 mg, 1.5 mg) in toluene (500 µL). The solution was stirred at 100 °C for 18 h. The resulting solution was analysed by $^1H$, $^{31}P\{^1H\}$, $^{31}P$ NMR spectroscopy.

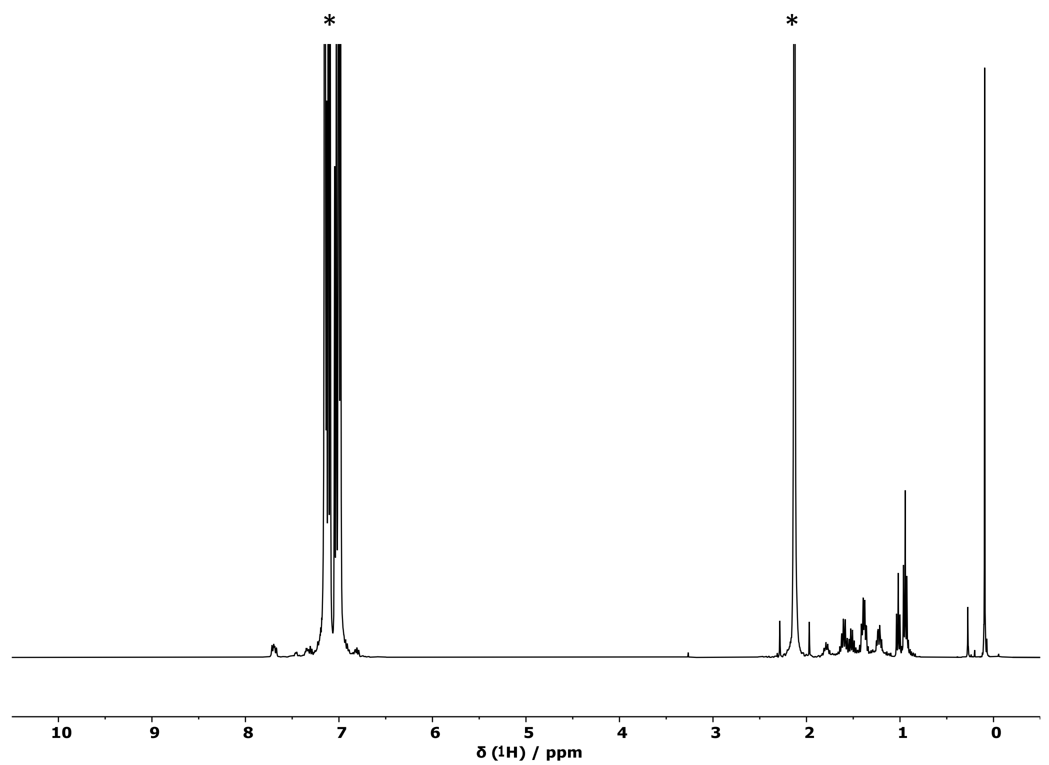


**Figure S6**: $^{1}H$ NMR spectrum for the reaction of $(Bu_3Sn)_3P$ (0.04 mmol) with PhCl and KHMDS in toluene at 100 °C for 18 h. Solvent resonances are marked with an asterisk and are truncated for clarity.

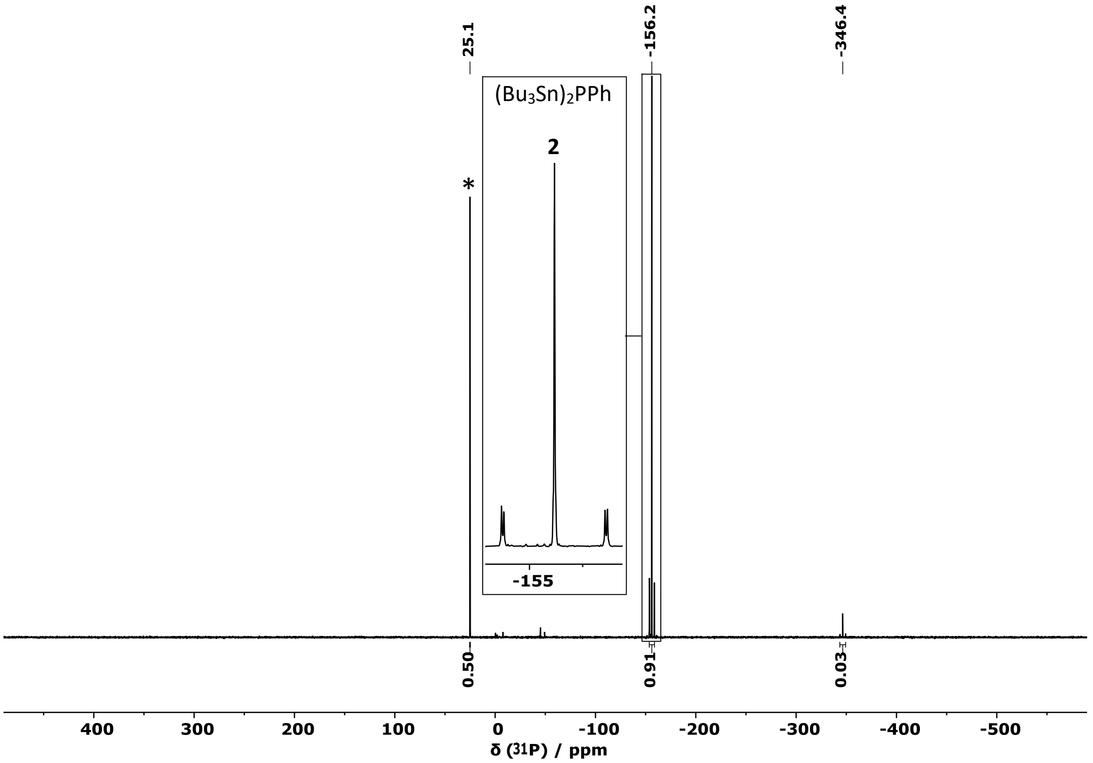


**Figure S7**: $^{31}P\{^{1}H\}$ NMR spectrum for the reaction of $(Bu_3Sn)_3P$ (0.04 mmol) with PhCl and KHMDS in toluene at 100 °C for 18 h. The insets show expansion of the signal attributed to $(Bu_3Sn)_2PPh$, highlighting the presence of $^{117/119}Sn$ satellites. * marks the internal standard $Ph_3PO$ (0.02 mmol).

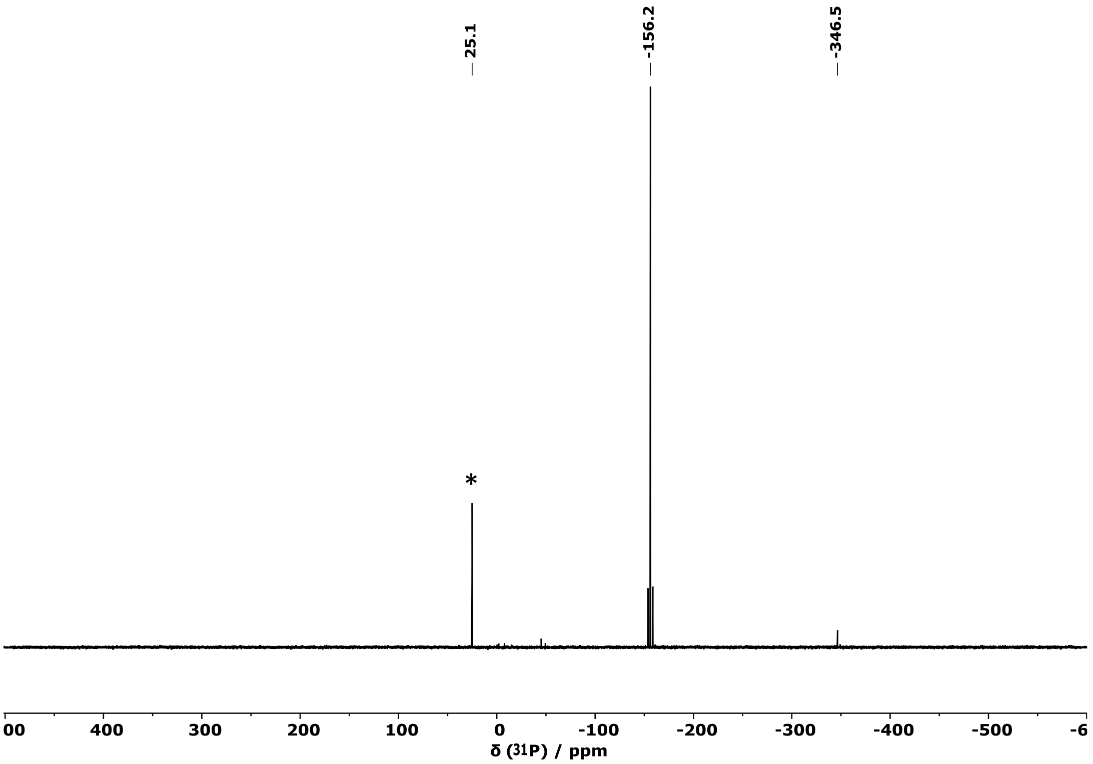


**Figure S8**: $^{31}P$ NMR spectrum for the reaction of $(Bu_3Sn)_3P$ (0.04 mmol) with PhCl and KHMDS in toluene at 100 °C for 18 h. * marks the internal standard $Ph_3PO$ (0.02 mmol).

For reasons of experimental expediency, during the optimization of the phenylation of $(Bu_3Sn)_3P$, acquisition of quick but non-quantitative $^{31}P\{^1H\}$ NMR spectra was used to analyse each experiment and to assess the relative conversions towards $(Bu_3Sn)_2PPh$. Although it did not provide quantitative conversions, it did allow for meaningful, qualitative comparisons between experiments.

**Table S1**: Optimisation of phenylation of $(Bu_3Sn)_3P$ using PhCl: screening of alkali metal salts and bases.[a]

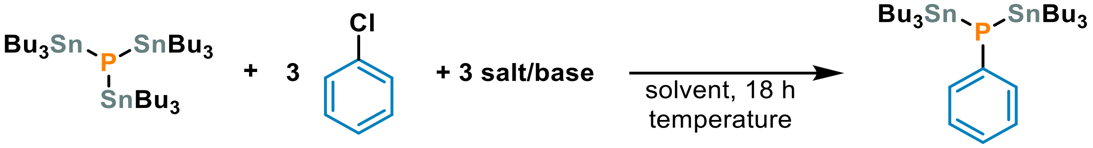


| Entry | Salt or Base used | Solvent (500 µL) | Temperature (°C) | Full conv. of $(Bu_3Sn)_3P$?[b] | Relative conv. to $(Bu_3Sn)_2PPh$ (%) |
|---|---|---|---|---|---|
| **1** | **KHMDS** | **toluene** | **100** | ✓ | **79** |
| 2 | - | toluene | 100 | ✗ | n.d. |
| 3 | NaHMDS | toluene | 100 | ✓ | 76 |
| 4 | LiHMDS | toluene | 100 | ✗ | n.d. |
| 5 | KF | THF | 60 | ✗ | n.d. |
| 6 | KCl | THF | 60 | ✗ | n.d. |
| 7 | KBr | THF | 60 | ✗ | n.d. |
| 8 | KI | THF | 60 | ✗ | n.d. |
| 9 | KH | toluene | 100 | ✗ | n.d. |
| 10 | KO*t*Bu | toluene | 100 | ✗ | n.d. |
| 11 | KO*t*Bu | THF | 60 | ✗ | n.d. |
| 12 | KO*t*Bu | DMSO | 100 | ✓ | n.d. |
| 13 | $PhCH_2K$ | toluene | 100 | ✗ | 8 |
| 14 | LiOMe | toluene | 100 | ✗ | n.d. |
| 15 | KOMe | toluene | 100 | ✗ | n.d. |
| 16 | $K_2CO_3$ | toluene | 100 | ✗ | n.d. |
| 17[c] | BuLi | toluene | −30→r.t. | ✗ | n.d. |
| 18[c] | MeLi | toluene | −30→r.t. | ✗ | n.d. |
| 19[c] | $Bu_2Mg$ | toluene | −30→r.t. | ✗ | n.d. |
| 20[c] | LDA | toluene | −30→r.t. | ✗ | 51 |
| 21 | Diphenylamine | toluene | 90 | ✗ | n.d. |
| 22 | 9H-Carbazole | toluene | 90 | ✗ | n.d. |

[a] The procedure was modified to use the indicated alkali metal salts and bases. [b] The full consumption of $(Bu_3Sn)_3P$ was assessed by $^{31}P\{^1H\}$ NMR spectroscopy and the disappearance of the corresponding signal at around −346 ppm. [c] A solution of $(Bu_3Sn)_3P$ and PhCl in toluene was prepared and cooled down to −30 °C. The respective bases were added, and the solutions stirred at −30 °C for 30 min before being allowed to warm to room temperature and further stirred for 18 h.

**Table S2**: Optimisation of phenylation of $(Bu_3Sn)_3P$ using PhCl and KHMDS: screening of reaction parameters.[a]

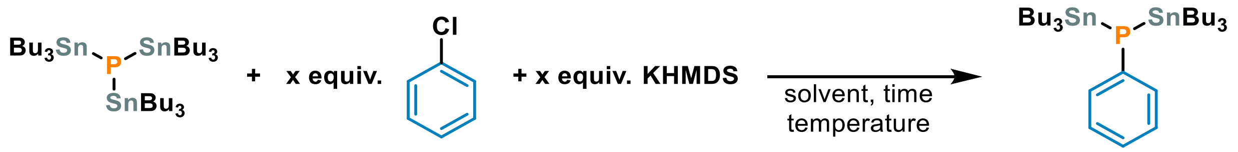


| Entry | Equivalents (x) | Solvent (500 µL) | Reaction time (h) | Temperature (°C) | Full conv. of $(Bu_3Sn)_3P$?[b] | Relative conv. to $(Bu_3Sn)_2PPh$ (%) |
|---|---|---|---|---|---|---|
| 1 | 3 | toluene | 2 | 100 | ✓ | 79 |
| 2 | 3 | THF | 2 | 50 | ✗ | 37 |
| 3 | 3 | MTBE | 2 | 50 | ✗ | 75 |
| 4 | 3 | MTBE | 2 | 80 (reflux) | ✗ | n.d. |
| 5 | 3 | MTBE | 90 | 50 | ✗ | 85 |
| **6** | **1.5** | **toluene** | **24** | **100** | ✗ | **91** |
| 7 | 1.5 | MTBE | 18 | 55 | ✗ | 87 |
| 8 | 1.1 | toluene | 6 | 100 | ✗ | 42 |
| 9 | 1.1 | toluene | 24 | 100 | ✗ | 61 |
| 10 | 1.1 | toluene | 48 | 100 | ✗ | 65 |
| **11** | **1** | **toluene** | **18** | **100** | ✗ | **69** |
| 12 | 2 | toluene | 18 | 100 | ✓ | 87 |

[a] The procedure was modified to use the corresponding reaction conditions. [b] The full consumption of $(Bu_3Sn)_3P$ was assessed by $^{31}P\{^1H\}$ NMR spectroscopy and the disappearance of the corresponding signal at around −346 ppm.

## S4. Representative procedure and optimisation for the protonation of $(Bu_3Sn)_2PPh$ to afford $PhPH_2$

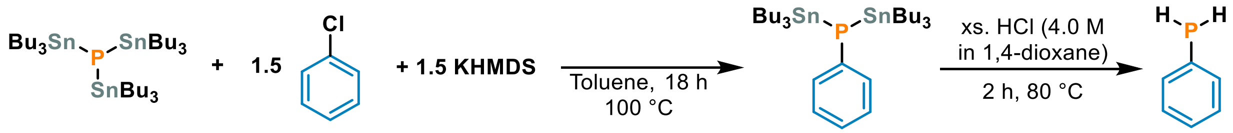


To a 10 mL Schlenk flask were added $(Bu_3Sn)_3P$ (0.04 mmol, 36.0 mg, 1 equiv.), PhCl (0.06 mmol, 6.1 µL, 1.5 equiv.) and KHMDS (0.06 mmol, 11.9 mg, 1.5 mg) in toluene (500 µL). The solution was stirred at 100 °C for 18 h. After cooling the solution in an ice bath, HCl (4 M in 1,4-dioxane, 0.1 mL, 0.4 mmol, excess) was added and the solution was allowed to warm to room temperature before heating at 80 °C for 2 h. The resulting solution was analysed by $^1H$, $^{31}P\{^1H\}$, $^{31}P$ NMR spectroscopy.

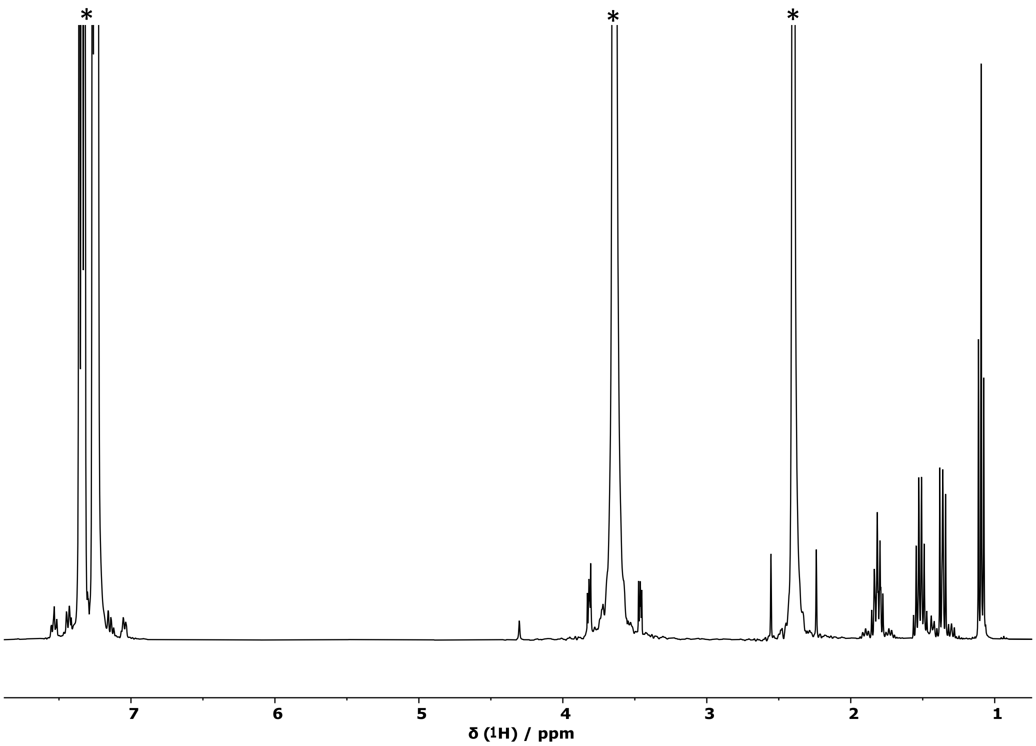


**Figure S9**: $^{1}$H NMR spectrum for the reaction of $(Bu_3Sn)_3P$ (0.04 mmol) with PhCl and KHMDS in toluene at 100 °C for 18 h and subsequent protonation using HCl. Solvent resonances are marked with an asterisk and are truncated for clarity.

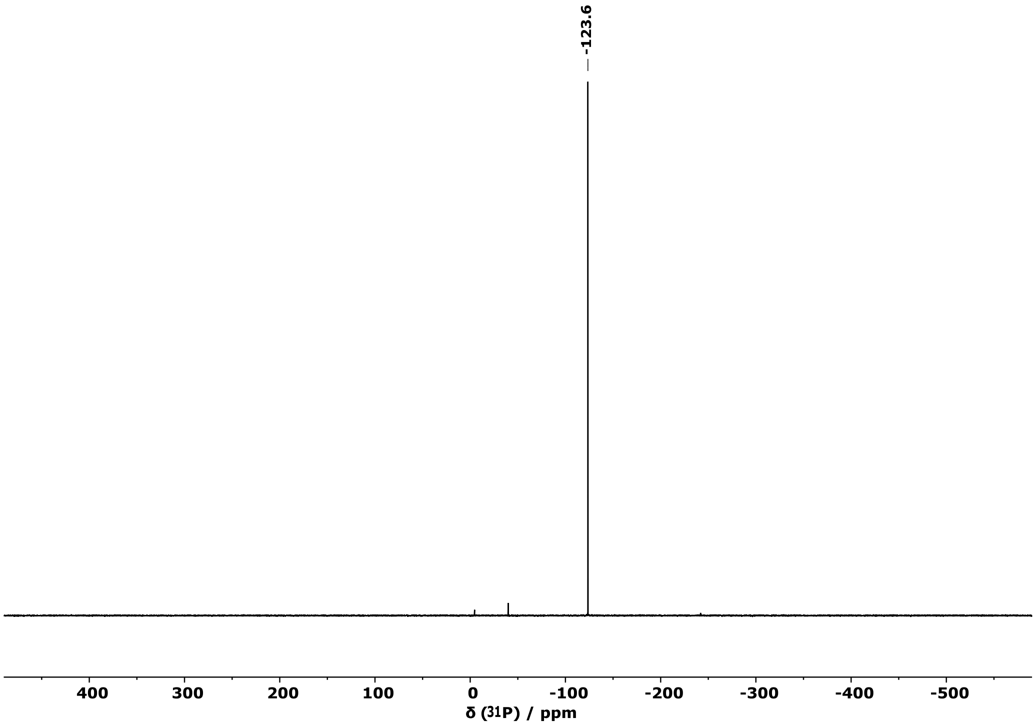


**Figure S10**: $^{31}P\{^{1}H\}$ NMR spectrum for the reaction of $(Bu_3Sn)_3P$ (0.04 mmol) with PhCl and KHMDS in toluene at 100 °C for 18 h and subsequent protonation using HCl.

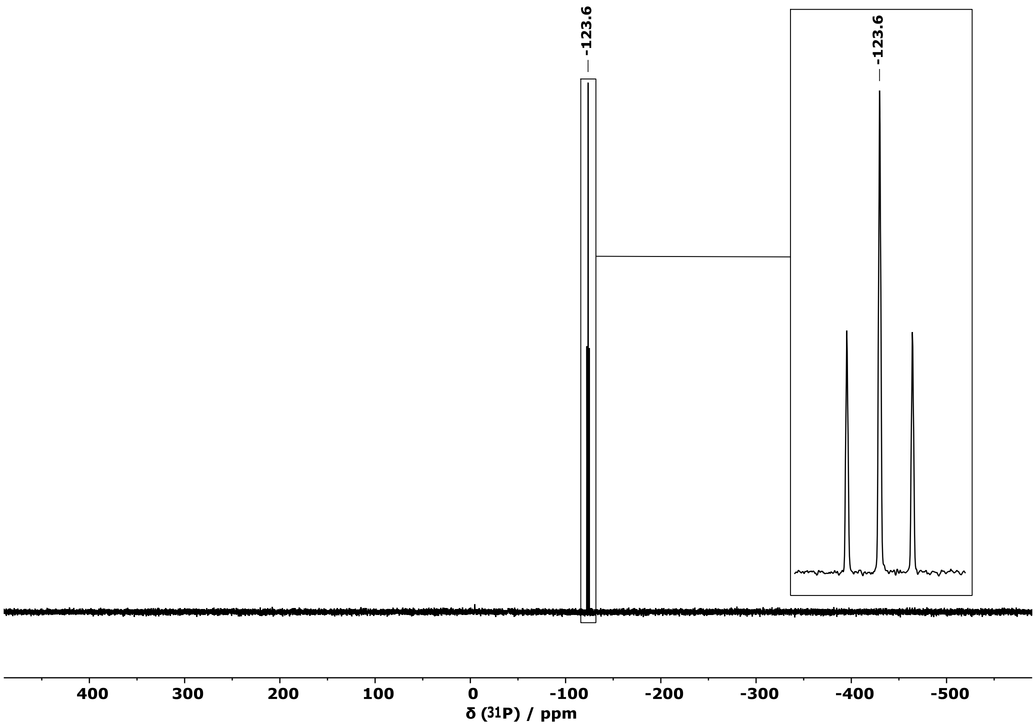


**Figure S11**: $^{31}P$ NMR spectrum for the reaction of $(Bu_3Sn)_3P$ (0.04 mmol) with PhCl and KHMDS in toluene at 100 °C for 18 h and subsequent protonation using HCl.

As judged by NMR spectroscopy, the protonation of *in situ* generated $(Bu_3Sn)_2PPh$ with an excess of HCl proceeded with full conversion of $(Bu_3Sn)_2PPh$ and selective formation of $PhPH_2$ at previously reported shifts with characteristic $^1J_{P-H}$ coupling of 199 Hz.[2] When this reaction was carried out with a stoichiometric amount (2 equiv. per P atom) of HCl, the signal of product $PhPH_2$ was shifted downfield to around −115.9 ppm, with similar $^1J_{P-H}$ coupling of 199 Hz. This downfield shift is probably due to the Lewis acidity of the concomitantly forming $Bu_3SnCl$ and the weak Lewis basicity of $PhPH_2$, forming a weak Lewis acid-base adduct.[3,4] Since an excess amount of HCl counteracts this adduct formation, a screening of the equivalents of HCl required to cleave the adduct was conducted, as shown in Table S3 below.

**Table S3**: Optimisation of protonation of *in situ* generated $(Bu_3Sn)_2PPh$ using HCl (0.4 M in 1,4-dioxane).[a]

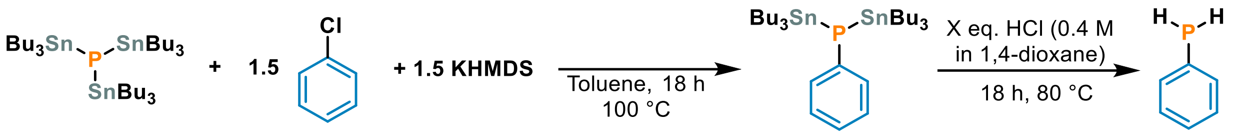


| Entry | Equivalents (X, per P) | Full conv. of $(Bu_3Sn)_2PPh$?[b] | Ratio of product signal at −123.6 ppm and −115.9 ppm[c] |
|---|---|---|---|
| 1 | 2 | ✓ | 0.08:1 |
| 2 | 3 | ✓ | 0.05:1 |
| 3 | 4 | ✓ | 0.1:1 |
| 4 | 5 | ✓ | 0.38:1 |
| 5 | 6 | ✓ | 1:0 |
| 6 | 7 | ✓ | 1:0 |

[a] The procedure was modified to use the corresponding reaction conditions. [b] The full conversion of $(Bu_3Sn)_2PPh$ was assessed by $^{31}P\{^1H\}$ NMR spectroscopy and the disappearance of the corresponding signal at around −156.9 ppm. [c] The ratios between the signals of $PhPH_2$ at −123.6 ppm and −115.9 ppm were assessed by integration in the $^{31}P\{^1H\}$ NMR spectrum. The signal at −123.6 ppm is in accordance with the literature.[2]

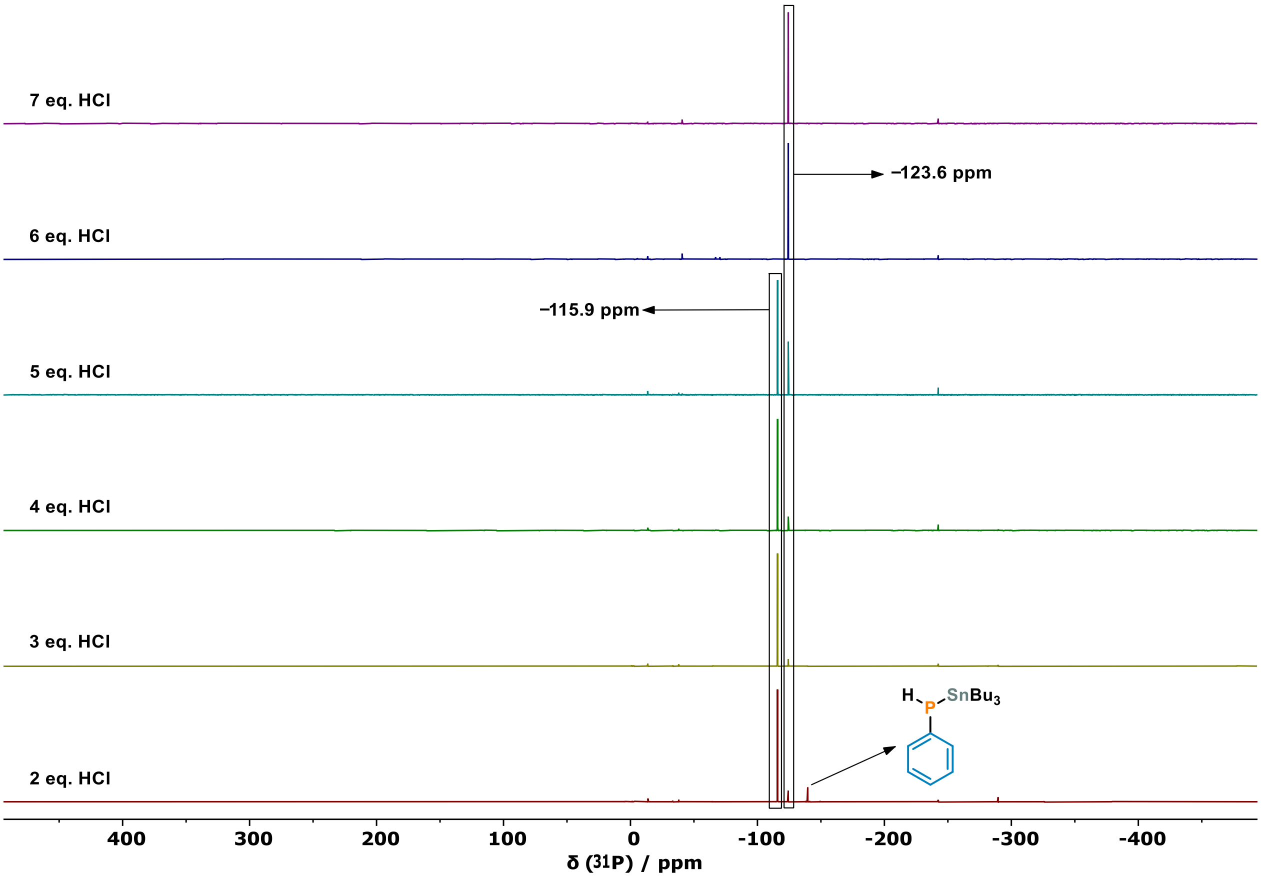


**Figure S12**: $^{31}P\{^1H\}$ NMR spectra of the reaction of $(Bu_3Sn)_3P$ (0.04 mmol) with KHMDS and PhCl towards $(Bu_3Sn)_2PPh$ in toluene at 100 °C for 18 h and subsequent protonation with differing equivalents (2-7, bottom to top) of HCl (0.4 M in 1,4-dioxane) at 80 °C for 18 h.

Based on these results, first scale-up reactions were performed starting from $P_4$. However, in these reactions, full conversion to the expected shift of $PhPH_2$ at −123.6 ppm was not observed using 6 equiv. of HCl. Therefore, 7 equiv. of HCl were instead used for scale-up reactions, which solved this issue. Furthermore, HCl in $Et_2O$ was used, as it was anticipated that this would make the isolation of $PhPH_2$ less challenging ($Et_2O$ more volatile than 1,4-dioxane).

## S5. 'One-pot' synthesis and isolation of $PhPH_2$ starting from $P_4$ *via* $(Bu_3Sn)_3P$ and $(Bu_3Sn)_2PPh$ (80 mmol scale) with $Bu_3SnCl$ recovery

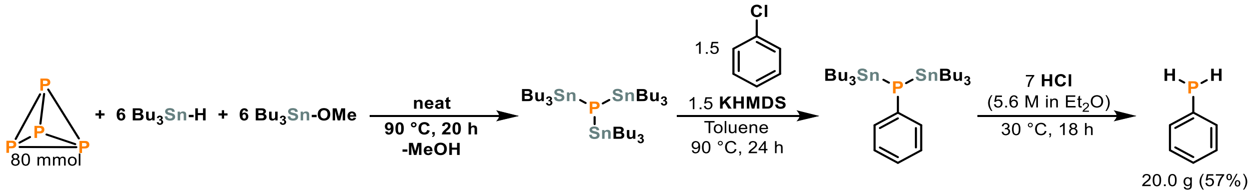


<u>Synthesis and isolation of $PhPH_2$:</u>

The following procedure was performed two times in parallel to get a total scale of 80 mmol. To a 1 L Schlenk flask were added $P_4$ (4.96 g, 40 mmol, 1 equiv.), $Bu_3SnH$ (63.5 mL, 240 mmol, 6 equiv. per $P_4$) and $Bu_3SnOMe$ (69.1 mL, 240 mmol, 6 equiv. per $P_4$). After stirring the mixture at 90 °C for 20 h, volatiles were removed under vacuum. To the resulting yellowish oil were added PhCl (24.3 mL, 240 mmol, 1.5 equiv. per P), KHMDS (47.88 g, 240 mmol, 1.5 equiv. per P) and toluene (400 mL). The mixture was heated at 90 °C while stirring for 24 h. Afterwards, volatiles were removed under vacuum at ~60 °C. To the resulting highly viscous, dark brown oil were added 300 mL $Et_2O$ before cooling the solution to 0 °C. To the cooled solution was added cold HCl (5.6 M in $Et_2O$, 240 mL, 1.12 mol, 7 equiv. per P) dropwise. The solution was warmed to room temperature and heated at 30 °C for 18 h. The resulting yellowish precipitate was removed by filtration using a frit (P4) and the two reaction solutions were combined. After concentrating the combined solution, fractional distillation was performed. $PhPH_2$ was isolated as a clear, colourless liquid (bp. 45 °C at 12.2 mbar; 20.0 g, 57%).

**$^{1}$H NMR** (400 MHz, 300 K, $C_6D_6$) δ[ppm] = 7.26 (td, *J* = 7.1, 2.2 Hz, 2H), 6.99 (m, 3H), 3.82 (d, $^{1}J(^{31}P-^{1}H)$ = 199.3 Hz, 2H).

**$^{31}$P{$^{1}$H} NMR** (162 MHz, 300 K, $C_6D_6$) δ[ppm] = –123.3 (s).

**$^{31}$P NMR** (162 MHz, 300 K, $C_6D_6$) δ[ppm] = –123.3 (t, $^{1}J(^{31}P-^{1}H)$ = 199.4 Hz).

**$^{13}$C{$^{1}$H} NMR** (101 MHz, 300 K, $CDCl_3$) δ[ppm] = 134.7 (d, *J* = 15.4 Hz), 128.5 (d, *J* = 7.6 Hz), 128.4 (d, *J* = 5.9 Hz), 128.0 (s).

NMR data are consistent with previous reports.[2]

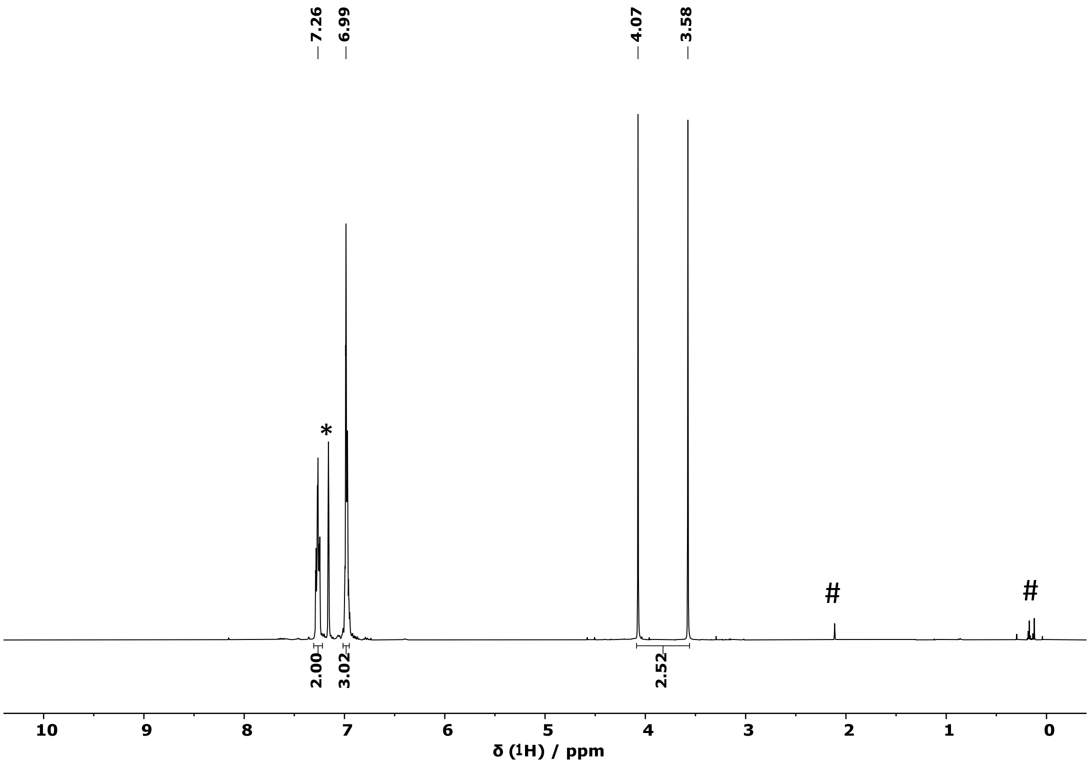


**Figure S13**: $^{1}H$ NMR spectrum of $PhPH_2$ in $C_6D_6$ (*). # marks minor unknown impurities.

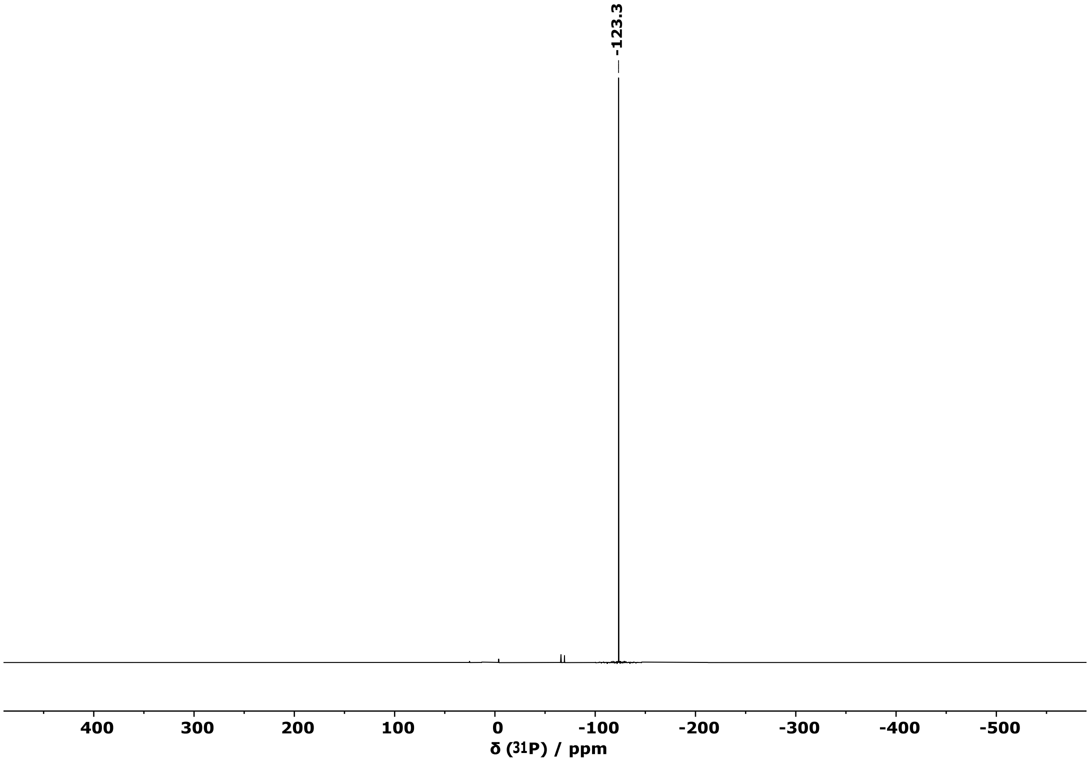


**Figure S14**: $^{31}P\{^{1}H\}$ NMR spectrum of $PhPH_2$ in $C_6D_6$.

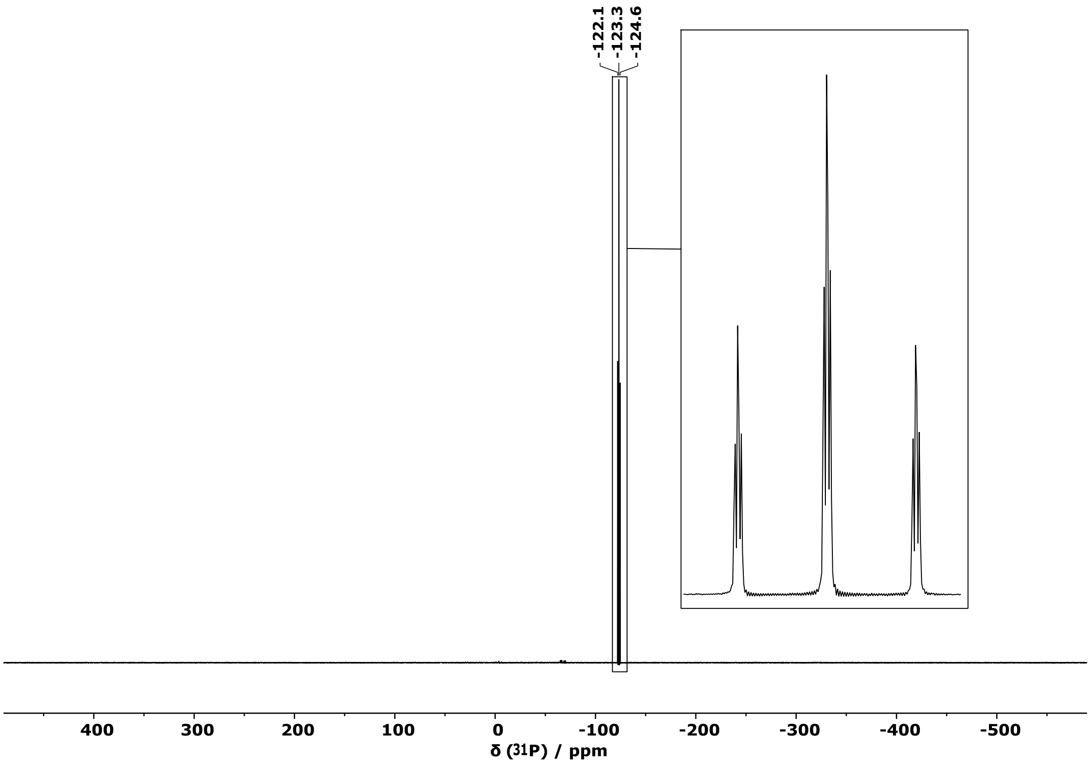


**Figure S15**: $^{31}P$ NMR spectrum of $PhPH_2$ in $C_6D_6$.

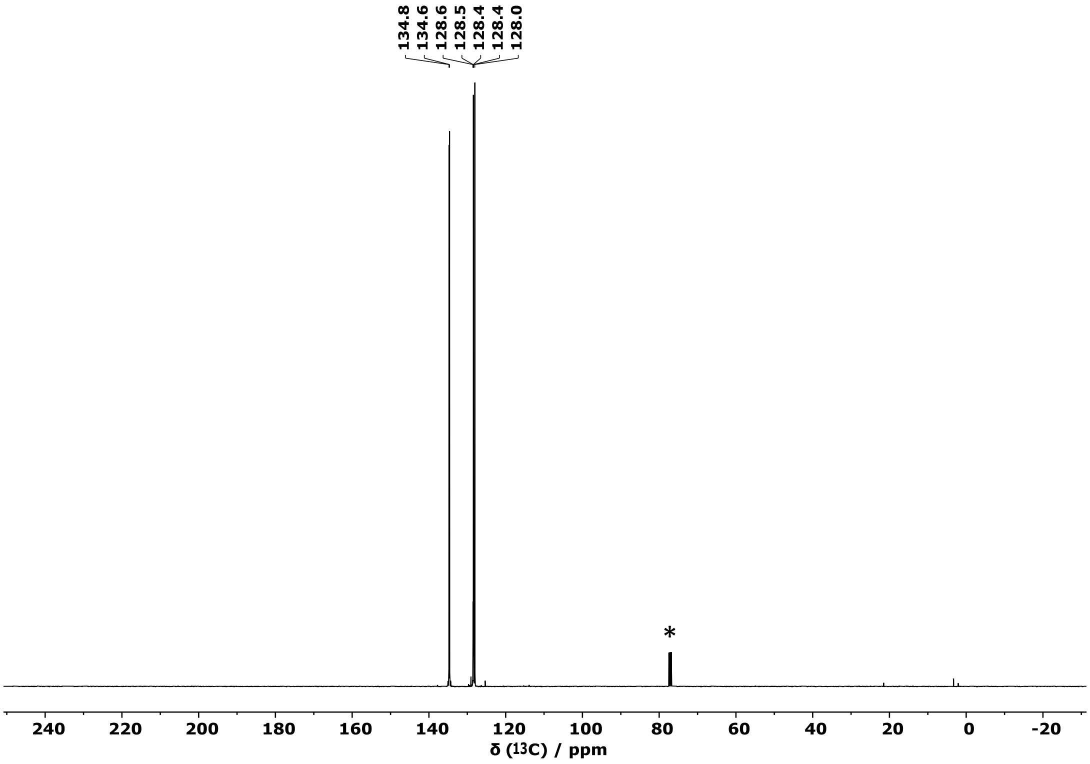


**Figure S16**: $^{13}C\{^1H\}$ NMR spectrum of $PhPH_2$ in $CDCl_3$ (*).

Quantification of residual Sn traces in isolated $PhPH_2$:

The quantification of the tin content was conducted using inductively coupled plasma optical emission spectroscopy (ICP-OES) using a spectrometer from SPECTRO. Calibration curves were recorded by measuring a series of standard samples. The sample was prepared as a ~460 µM solution in 18% HCl using the following procedure:

To 76 mg of isolated $PhPH_2$, 18% HCl (15 mL) was added at room temperature under air. The mixture was shaken and then stored for one day to allow the sample to dissolve fully. The resulting colourless solution was used for ICP-OES analysis. Literature shows that $Bu_3SnCl$ is very soluble in acidic aqueous solutions; therefore, all Sn-containing components should be solubilised sufficiently for detection.[5]

**Table S4**: Sn concentration detected by ICP-OES analyses.

| **No.** | **Sample 1** Conc. of Sn (µM) |
|---|---|
| 1 | 11.2 |
| 2 | 12.6 |
| 3 | 13.3 |
| **Average** | **12.3** |

The average concentration of 12.3 µM of Sn corresponds to a weight/weight content of 288 ppm of Sn atoms per $PhPH_2$.

Recovery of $Bu_3SnCl$:

The remaining orange suspension from the fractional distillation was placed under vacuum ($10^{-3}$ mbar) to remove all remaining volatiles. Subsequently, the suspension was distilled at elevated conditions (120 °C, $10^{-5}$ mbar). After filtering the resulting colourless distillate, $Bu_3SnCl$ could be recovered as a clear and colourless oil (292.4 g, 93% relative to $Bu_3Sn$ moieties originally used).

**$^1$H NMR** (400 MHz, 300 K, $CDCl_3$) δ[ppm] = 1.71-1.48 (m, 2H), 1.41-1.22 (m, 4H), 0.88 (t, $J$ = 7.3 Hz, 3H).

**$^{13}$C{$^1$H} NMR** (101 MHz, 300 K, $CDCl_3$) δ[ppm] = 27.8 (s), 26.8 (s), 17.6 (s), 13.6 (s).

**$^{119}$Sn{$^1$H} NMR** (121 MHz, 300 K, $CDCl_3$) δ[ppm] = 155.5 (s).

NMR data are consistent with previous reports.[6]

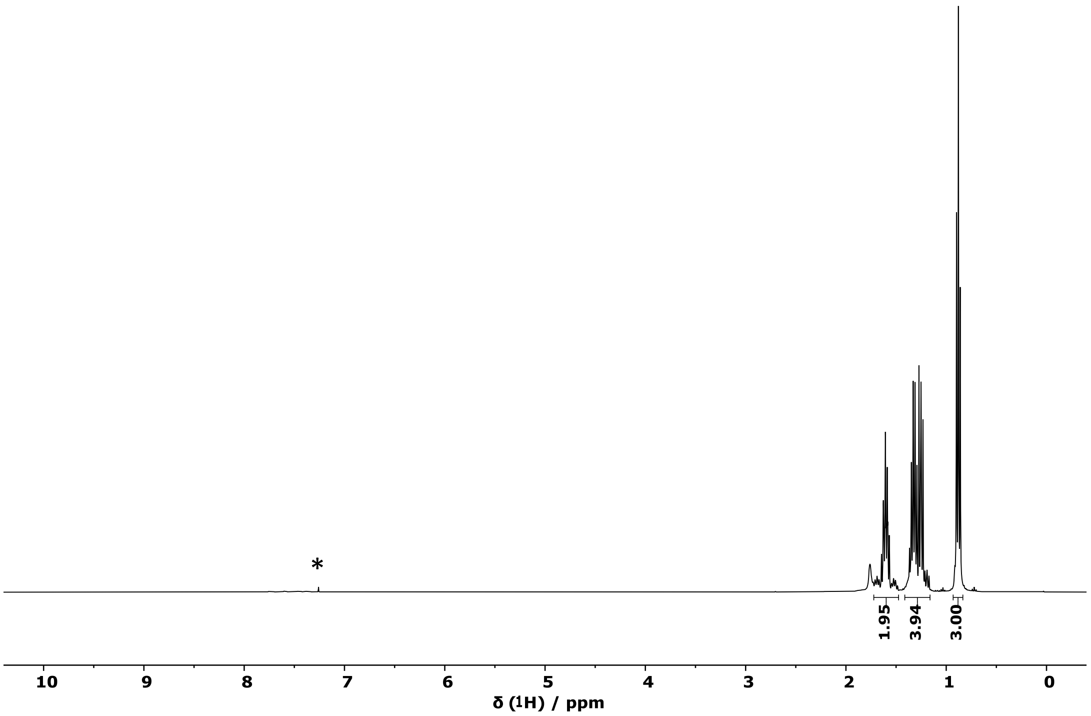


**Figure S17**: $^{1}$H NMR spectrum of $Bu_3SnCl$ in $CDCl_3$ (*).

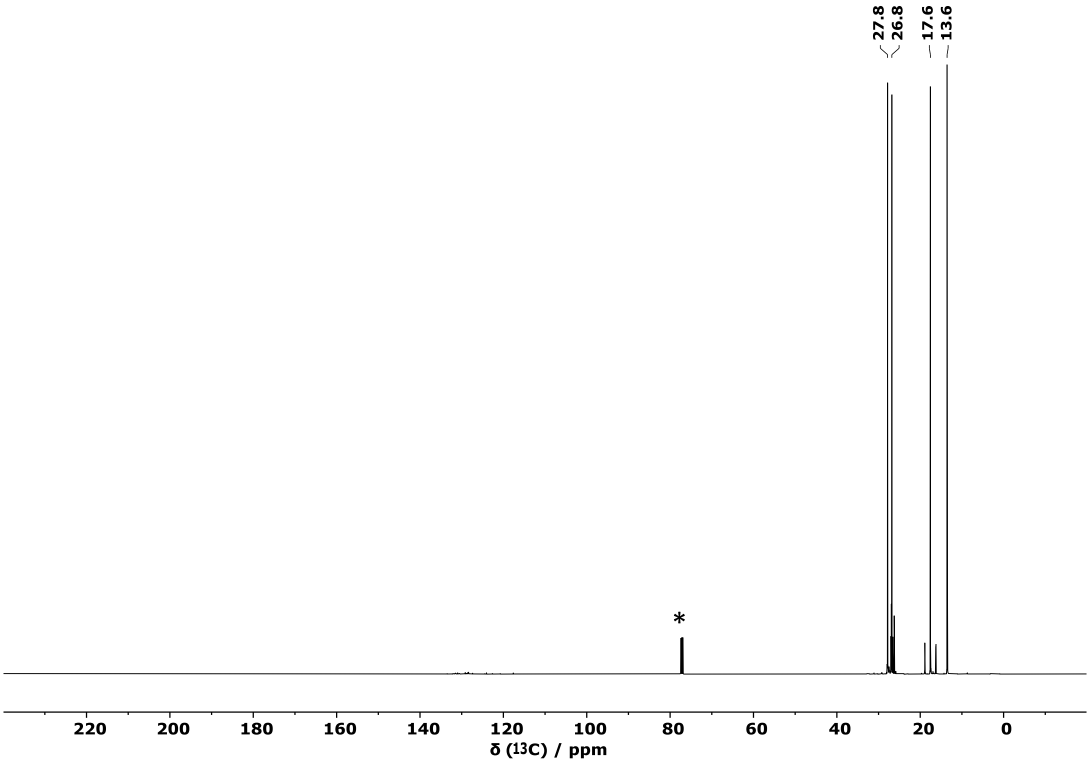


**Figure S18**: $^{13}C\{^{1}H\}$ NMR spectrum of $Bu_3SnCl$ in $CDCl_3$ (*).

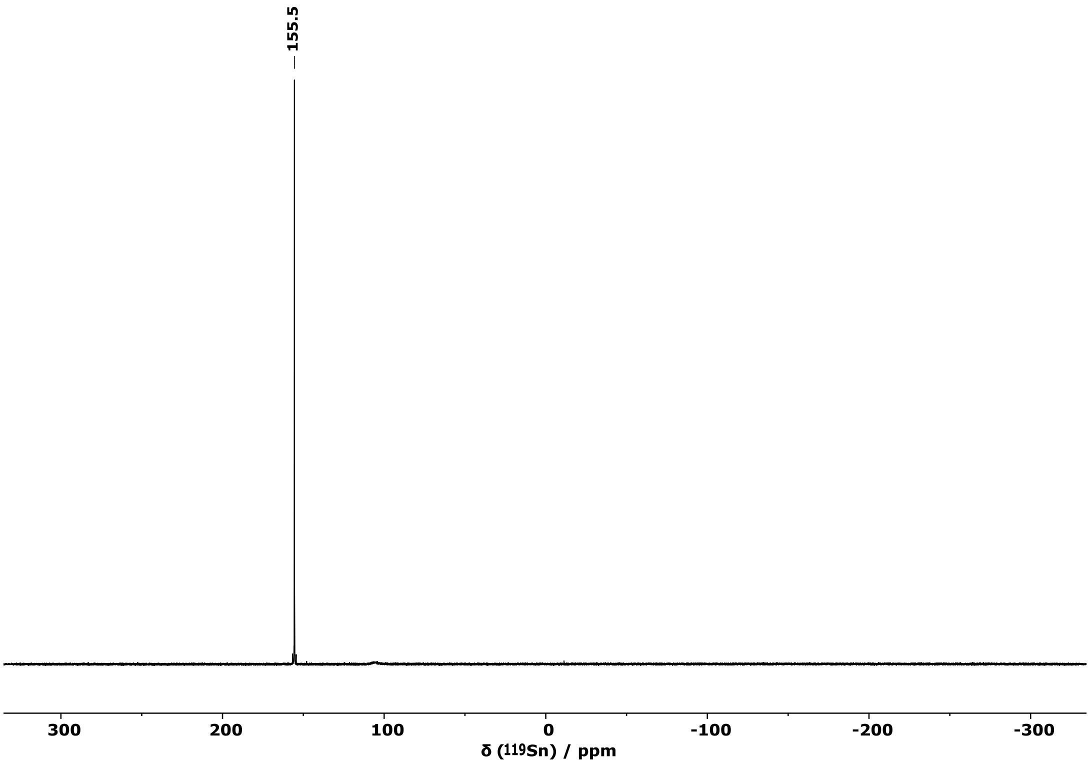


**Figure S19**: $^{119}Sn\{^{1}H\}$ NMR spectrum of $Bu_3SnCl$ in $CDCl_3$.

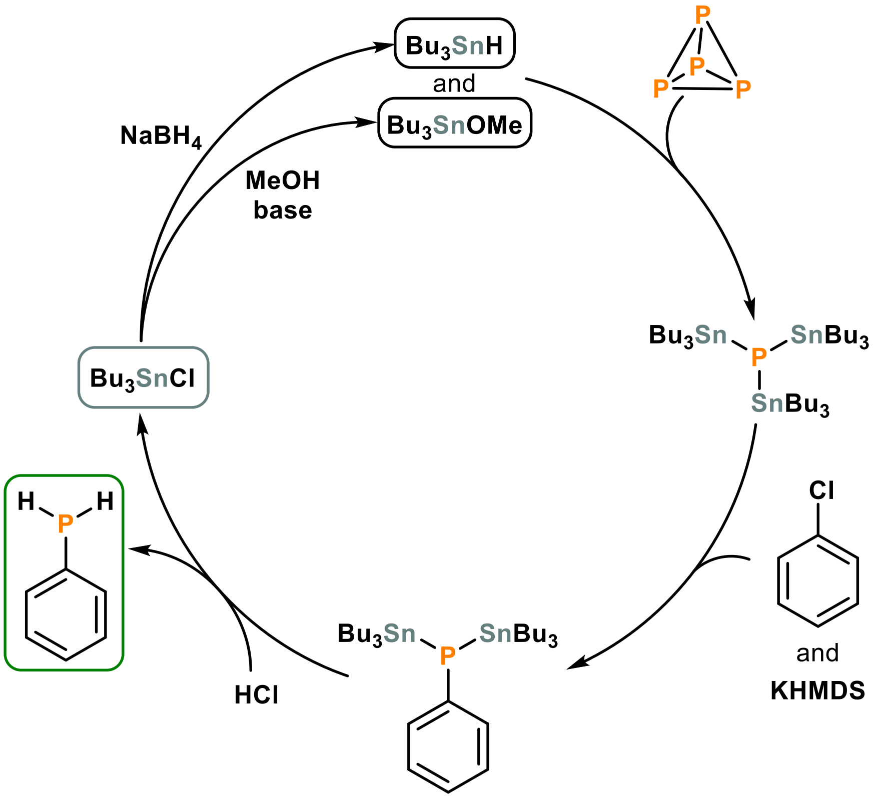


**Figure S20**: Proposed closed synthetic loop for the generation of $PhPH_2$.

## S6. Characterization of reactions of $(Bu_3Sn)_3P$ and aryl/alkyl halides on 0.04 mmol scale

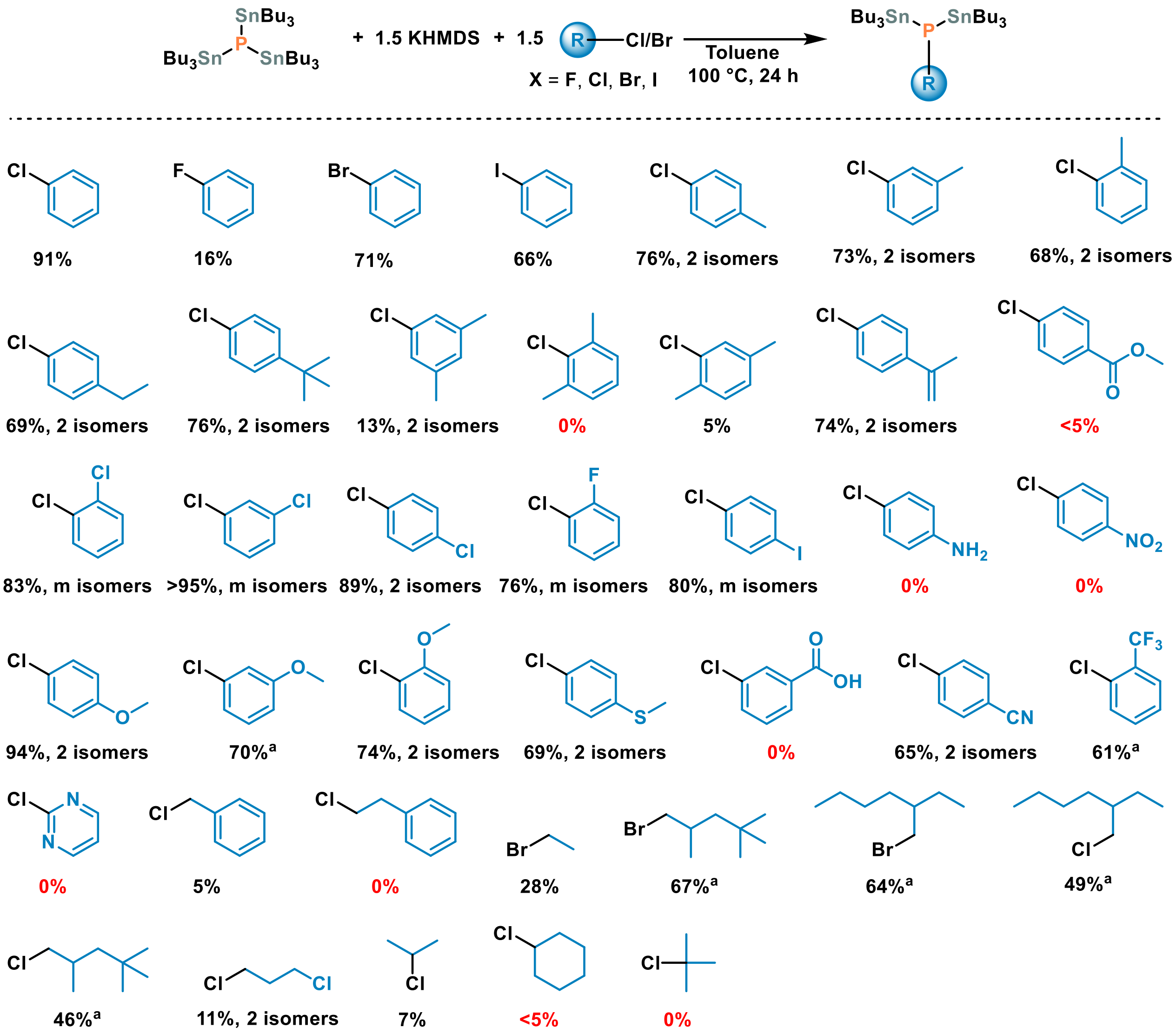


**Figure S21**: Substrate scope of the reaction of $(Bu_3Sn)_3P$ (0.04 mmol) and different aryl/alkyl halides. The procedure of chapter 3 was modified to use the corresponding substrate. Conversions stated above (spectroscopic yields) were determined using the method described in chapter 3, with $Ph_3PO$ as an internal standard (0.02 mmol) to calculate relative yields. Yields highlighted in red were considered as unsuccessful and the corresponding NMR spectra will not be displayed. [a] Conversions (spectroscopic yield) were determined using quantitative $^{31}P\{^1H\}$ NMR (zgig30, D1 = 30 s, inverse gated decoupled). m = multiple.

## S6.1 Reaction with fluorobenzene

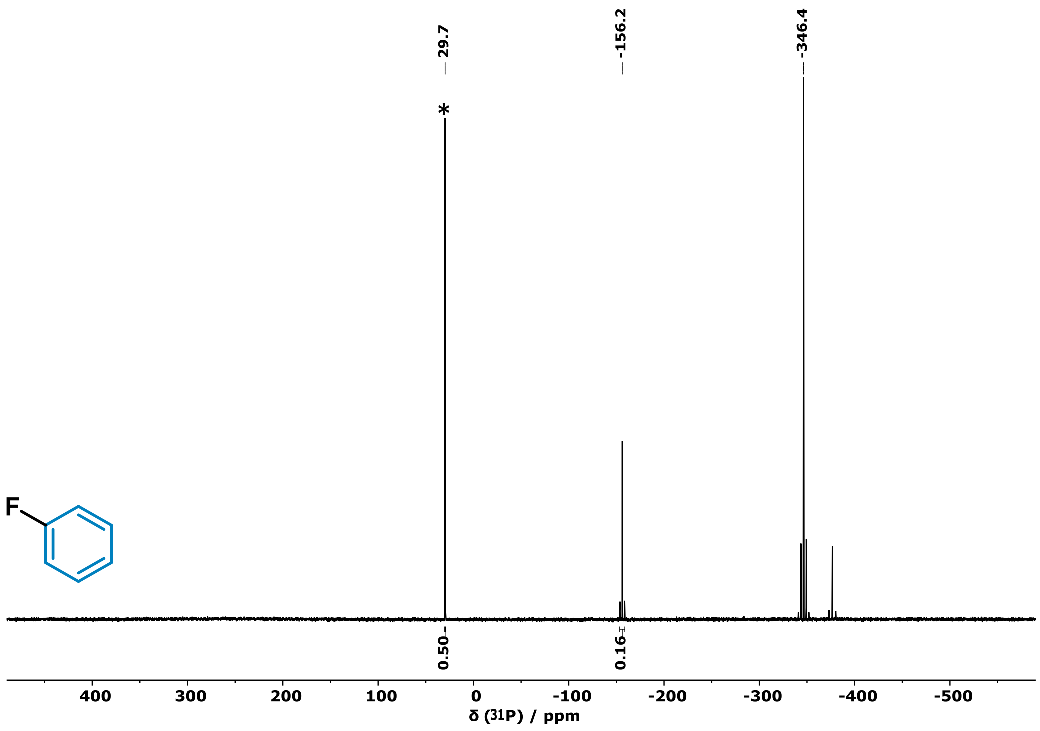


**Figure S22**: $^{31}P\{^{1}H\}$ NMR spectrum for the reaction of $(Bu_3Sn)_3P$ (0.04 mmol) and fluorobenzene with KHMDS in toluene. * marks the internal standard $Ph_3PO$ (0.02 mmol).

## S6.2 Reaction with bromobenzene

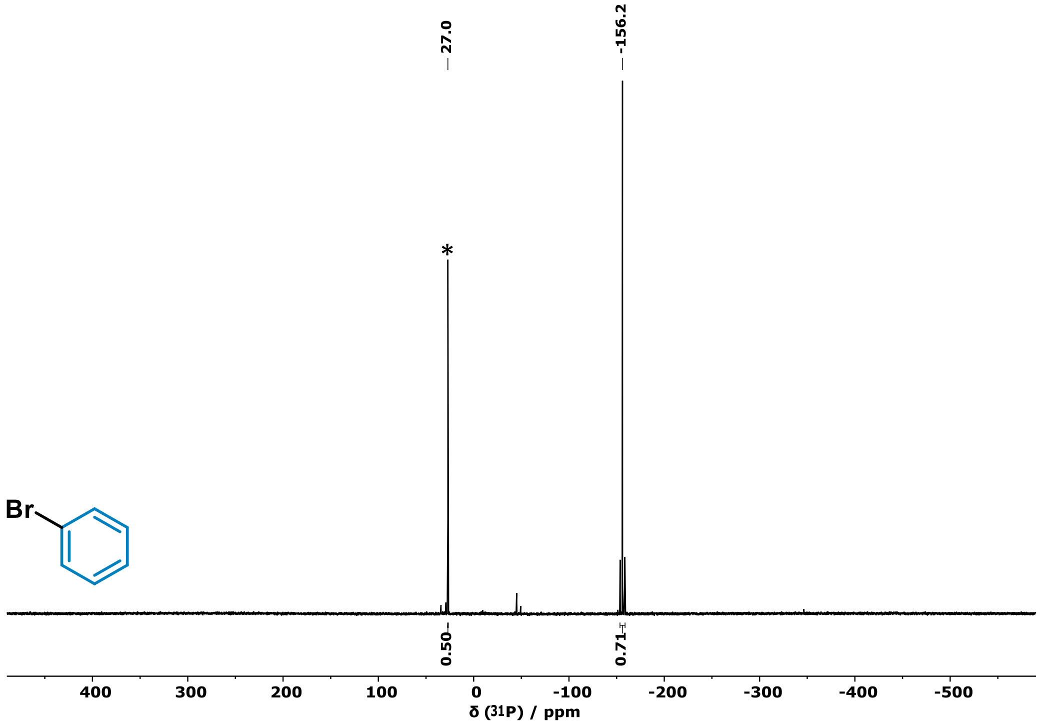


**Figure S23**: $^{31}P\{^{1}H\}$ NMR spectrum for the reaction of $(Bu_3Sn)_3P$ (0.04 mmol) and bromobenzene with KHMDS in toluene. * marks the internal standard $Ph_3PO$ (0.02 mmol).

## S6.3 Reaction with iodobenzene

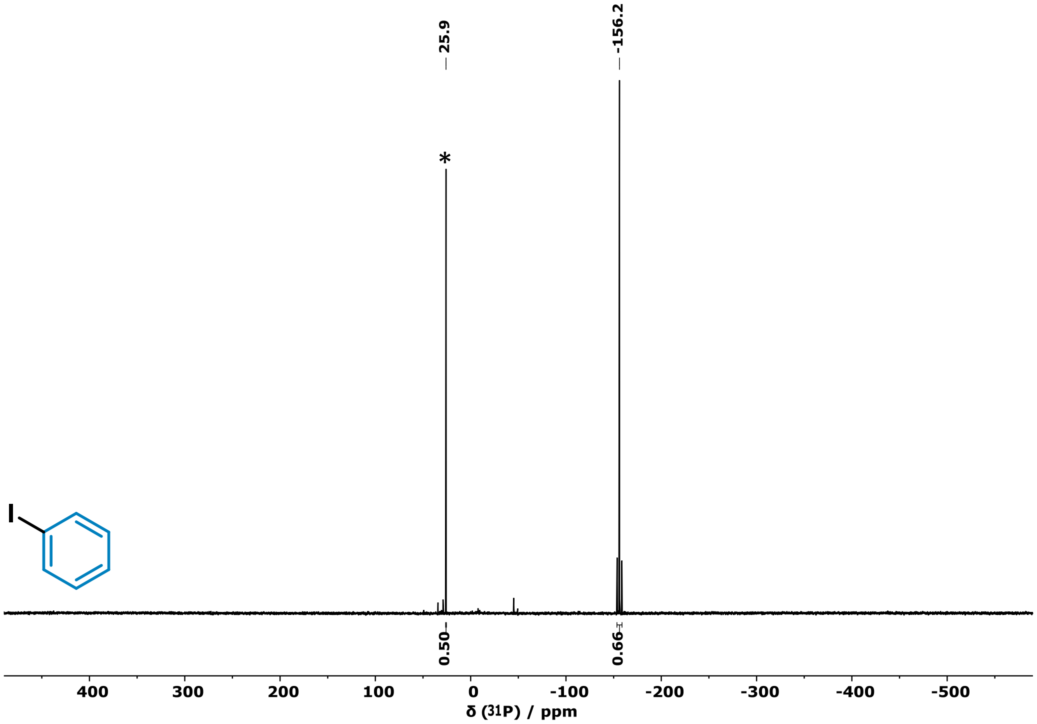


**Figure S24**: $^{31}P\{^{1}H\}$ NMR spectrum for the reaction of $(Bu_3Sn)_3P$ (0.04 mmol) and iodobenzene with KHMDS in toluene. * marks the internal standard $Ph_3PO$ (0.02 mmol).

## S6.4 Reaction with 4-chlorotoluene

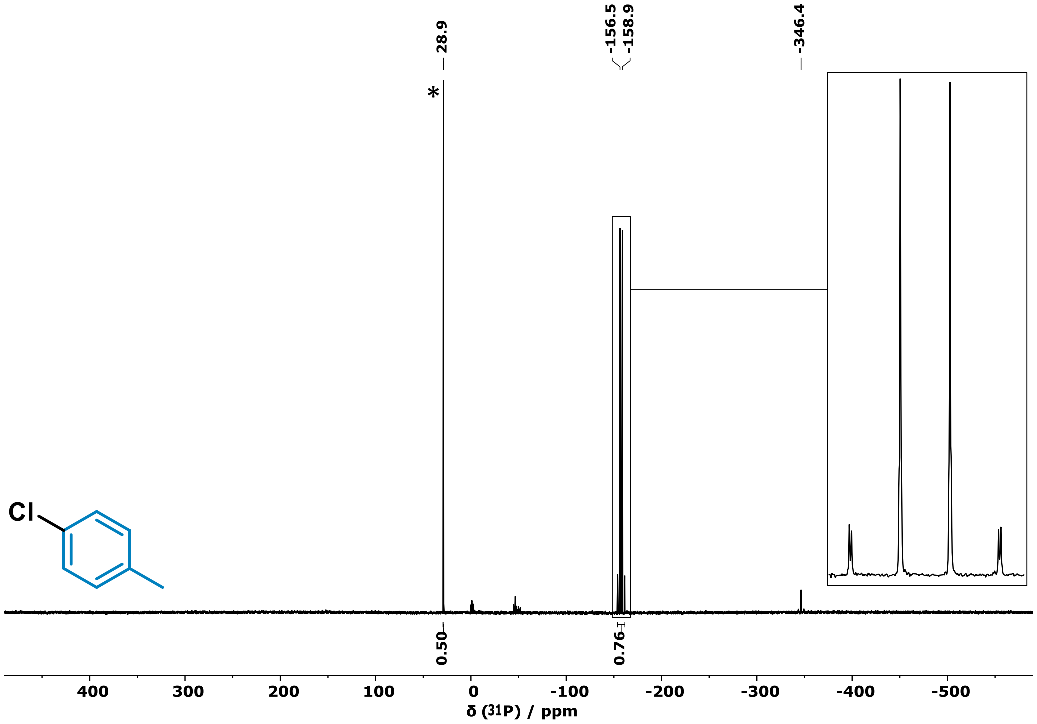


**Figure S25**: $^{31}P\{^{1}H\}$ NMR spectrum for the reaction of $(Bu_3Sn)_3P$ (0.04 mmol) and 4-chlorotoluene with KHMDS in toluene. * marks the internal standard $Ph_3PO$ (0.02 mmol).

## S6.5 Reaction with 3-chlorotoluene

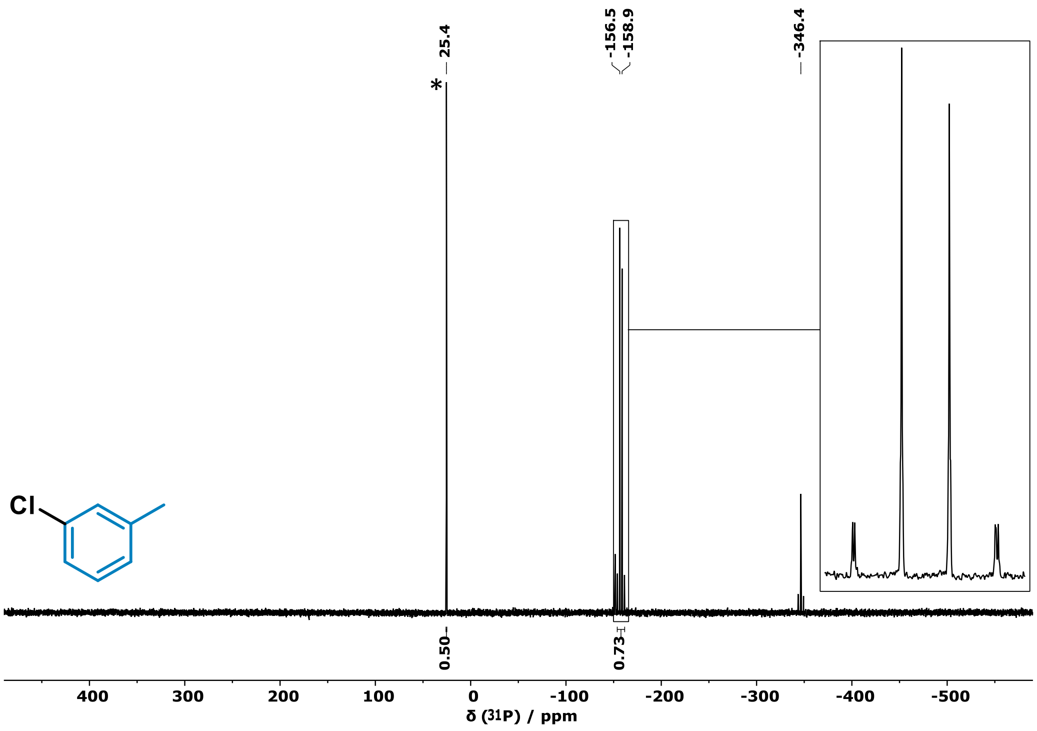


**Figure S26**: $^{31}P\{^{1}H\}$ NMR spectrum for the reaction of $(Bu_3Sn)_3P$ (0.04 mmol) and 3-chlorotoluene with KHMDS in toluene. * marks the internal standard $Ph_3PO$ (0.02 mmol).

## S6.6 Reaction with 2-chlorotoluene

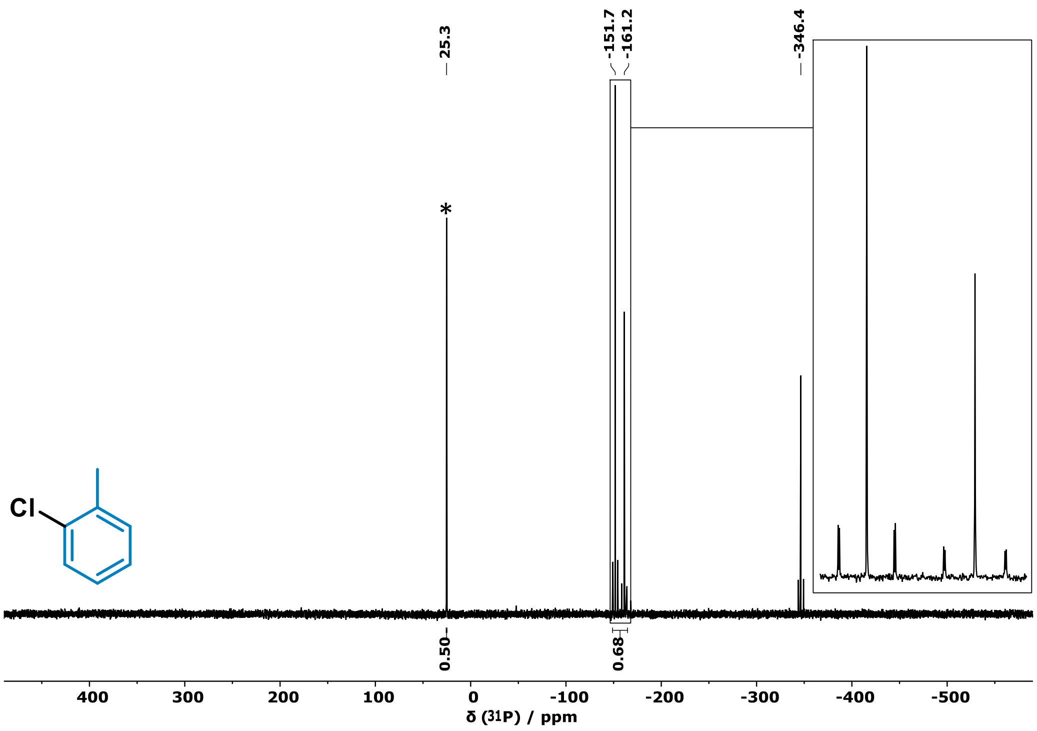


**Figure S27**: $^{31}P\{^{1}H\}$ NMR spectrum for the reaction of $(Bu_3Sn)_3P$ (0.04 mmol) and 2-chlorotoluene with KHMDS in toluene. * marks the internal standard $Ph_3PO$ (0.02 mmol).

## S6.7 Reaction with 1-chloro-4-ethylbenzene

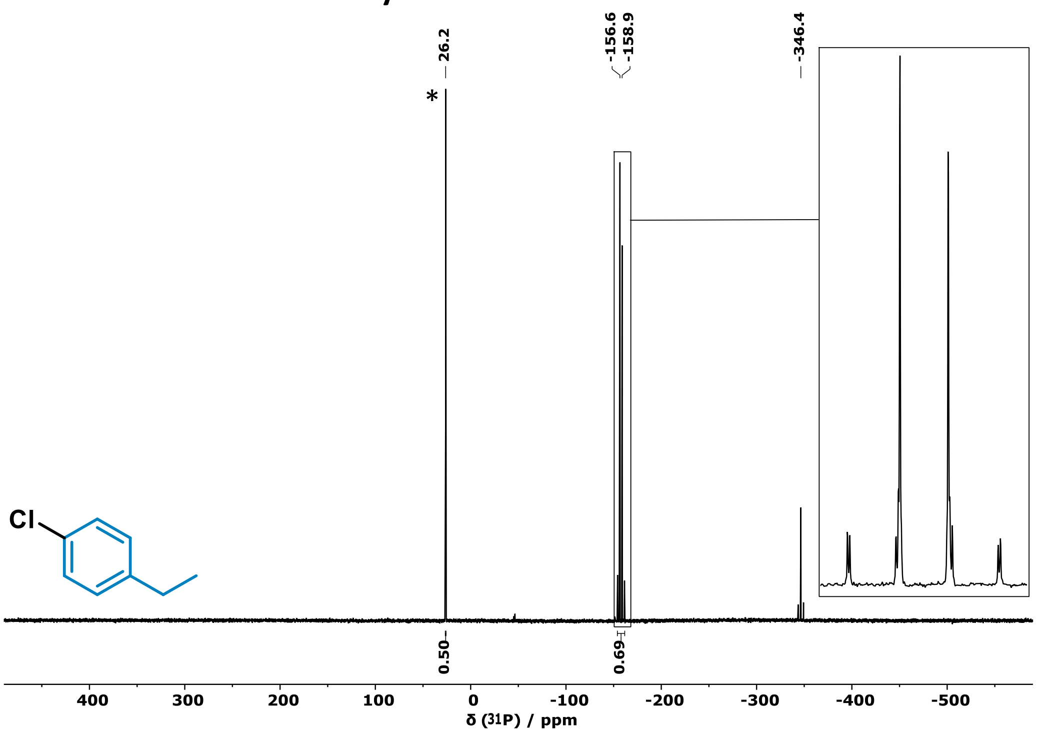


**Figure S28**: $^{31}P\{^{1}H\}$ NMR spectrum for the reaction of $(Bu_3Sn)_3P$ (0.04 mmol) and 1-chloro-4-ethylbenzene with KHMDS in toluene. * marks the internal standard $Ph_3PO$ (0.02 mmol).

## S6.8 Reaction with 1-(*tert*-butyl)-4-chlorobenzene

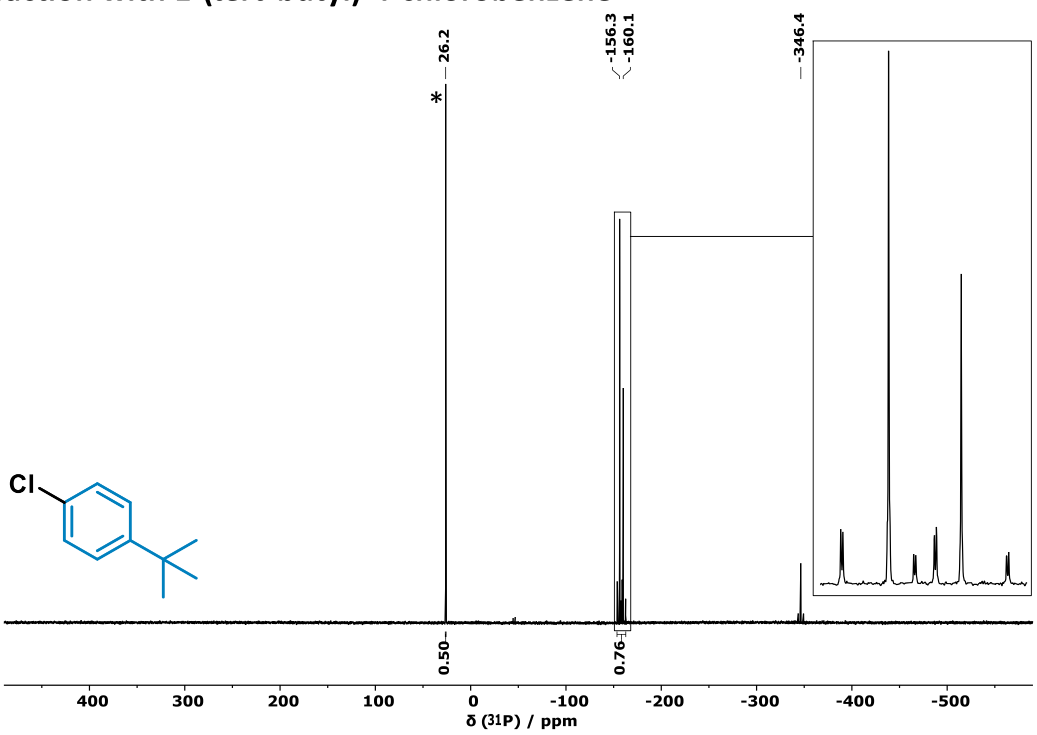


**Figure S29**: $^{31}P\{^{1}H\}$ NMR spectrum for the reaction of $(Bu_3Sn)_3P$ (0.04 mmol) and 1-(*tert*-butyl)-4-chlorobenzene with KHMDS in toluene. * marks the internal standard $Ph_3PO$ (0.02 mmol).

## S6.9 Reaction with 1-chloro-3,5-dimethylbenzene

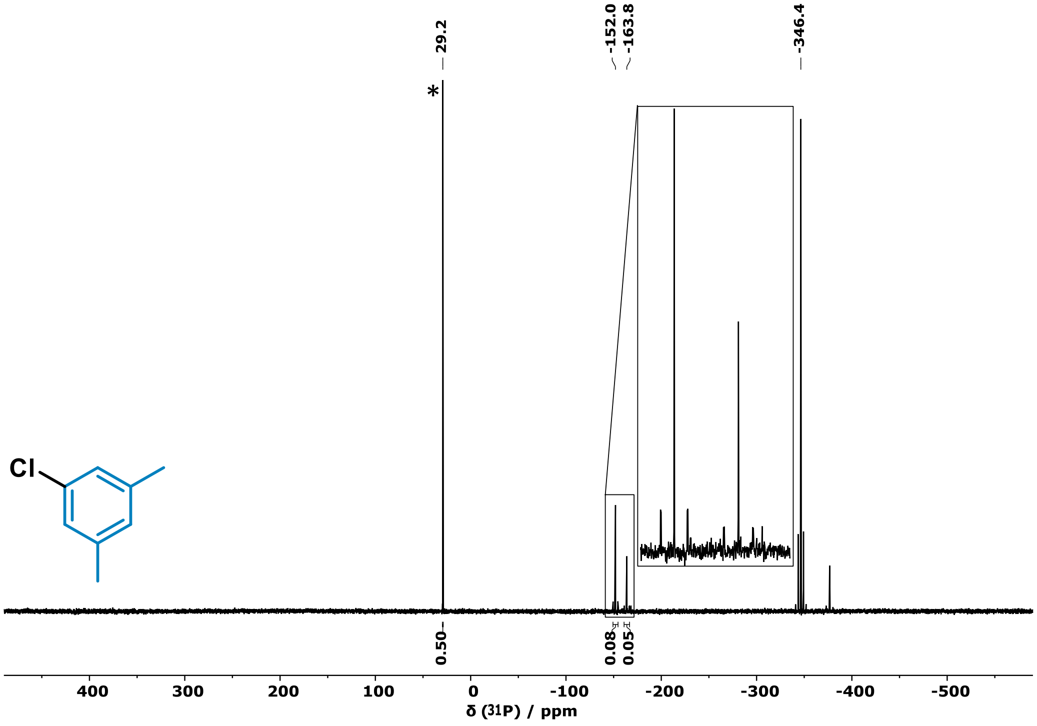


**Figure S30**: $^{31}P\{^1H\}$ NMR spectrum for the reaction of $(Bu_3Sn)_3P$ (0.04 mmol) and 1-chloro-3,5-dimethylbenzene with KHMDS in toluene. * marks the internal standard $Ph_3PO$ (0.02 mmol).

## S6.10 Reaction with 2-chloro-1,4-dimethylbenzene

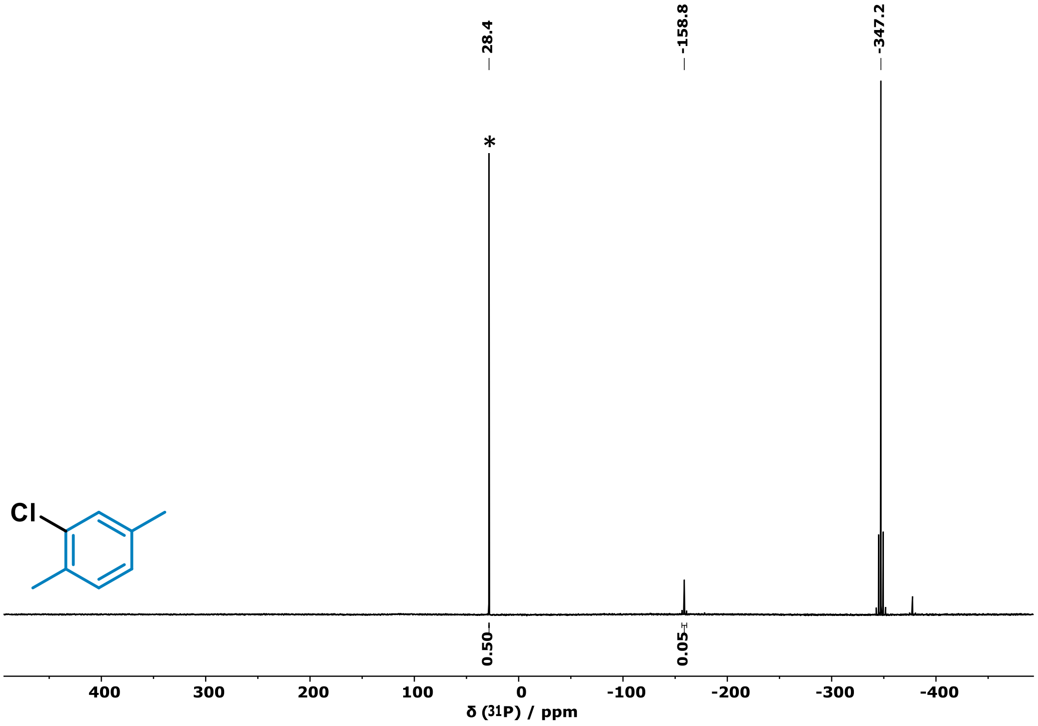


**Figure S31**: $^{31}P\{^1H\}$ NMR spectrum for the reaction of $(Bu_3Sn)_3P$ (0.04 mmol) and 2-chloro-1,4-dimethylbenzene with KHMDS in toluene. * marks the internal standard $Ph_3PO$ (0.02 mmol).

## S6.11 Reaction with 1-chloro-4-(prop-1-en-2-yl)benzene

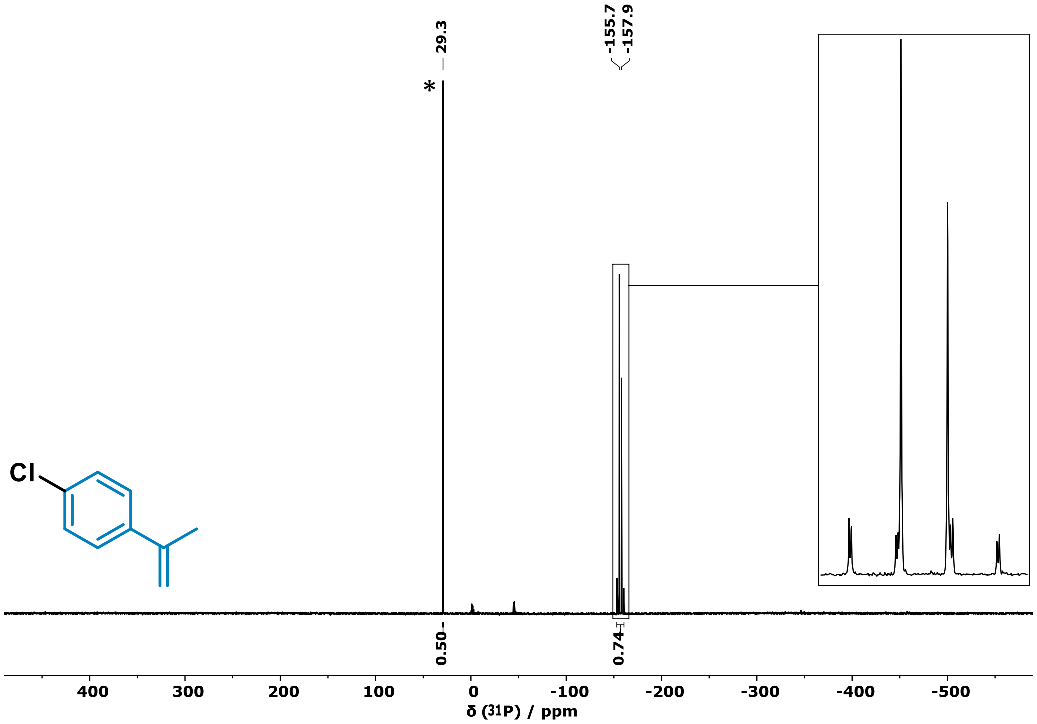


**Figure S32**: $^{31}P\{^{1}H\}$ NMR spectrum for the reaction of $(Bu_3Sn)_3P$ (0.04 mmol) and 1-chloro-4-(prop-1-en-2-yl)benzene with KHMDS in toluene. * marks the internal standard $Ph_3PO$ (0.02 mmol).

## S6.12 Reaction with 1,2-dichlorobenzene

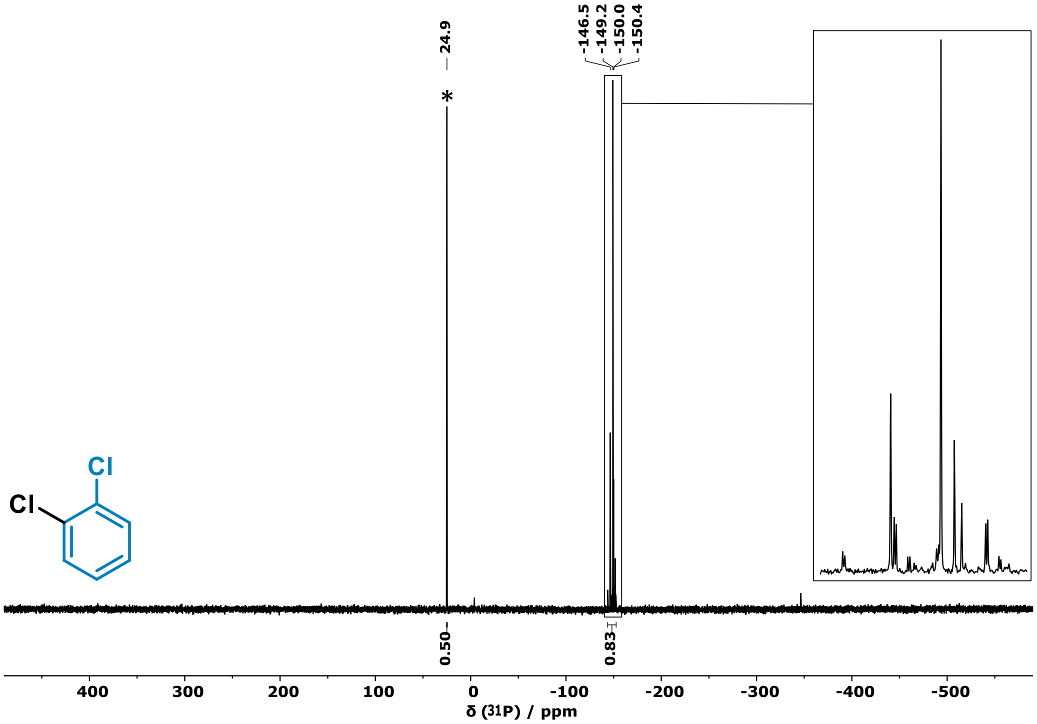


**Figure S33**: $^{31}P\{^{1}H\}$ NMR spectrum for the reaction of $(Bu_3Sn)_3P$ (0.04 mmol) and 1,2-dichlorobenzene with KHMDS in toluene. * marks the internal standard $Ph_3PO$ (0.02 mmol).

## S6.13 Reaction with 1,3-dichlorobenzene

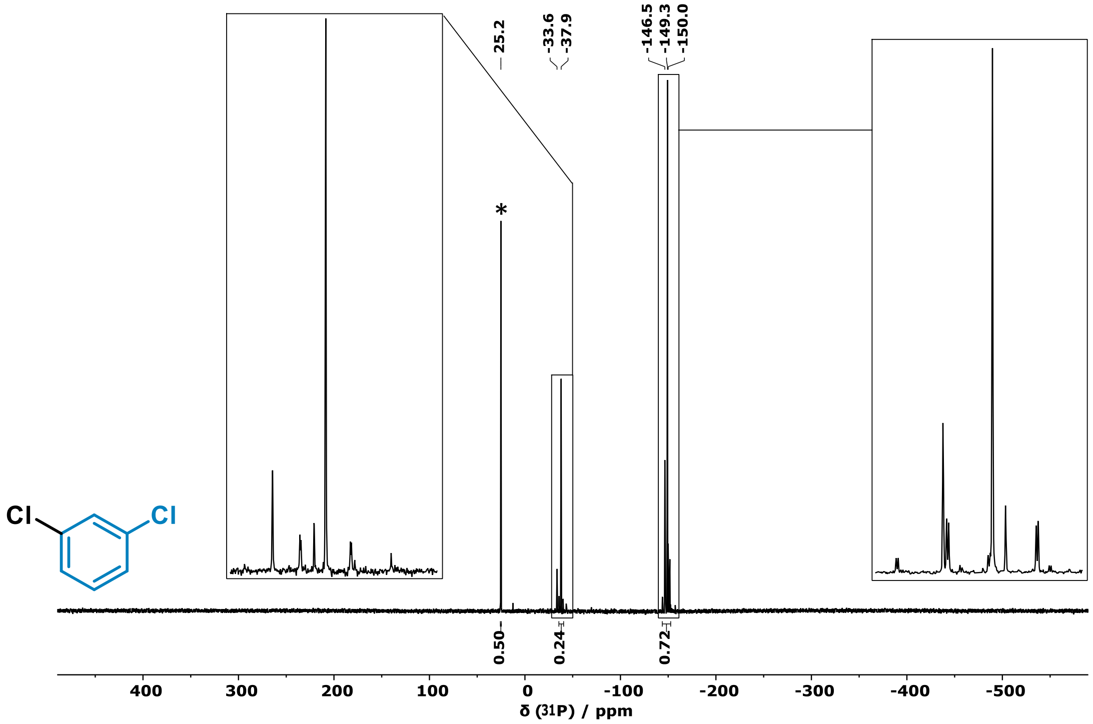


**Figure S34**: $^{31}P\{^{1}H\}$ NMR spectrum for the reaction of $(Bu_3Sn)_3P$ (0.04 mmol) and 1,3-dichlorobenzene with KHMDS in toluene. * marks the internal standard $Ph_3PO$ (0.02 mmol).

## S6.14 Reaction with 1,4-dichlorobenzene

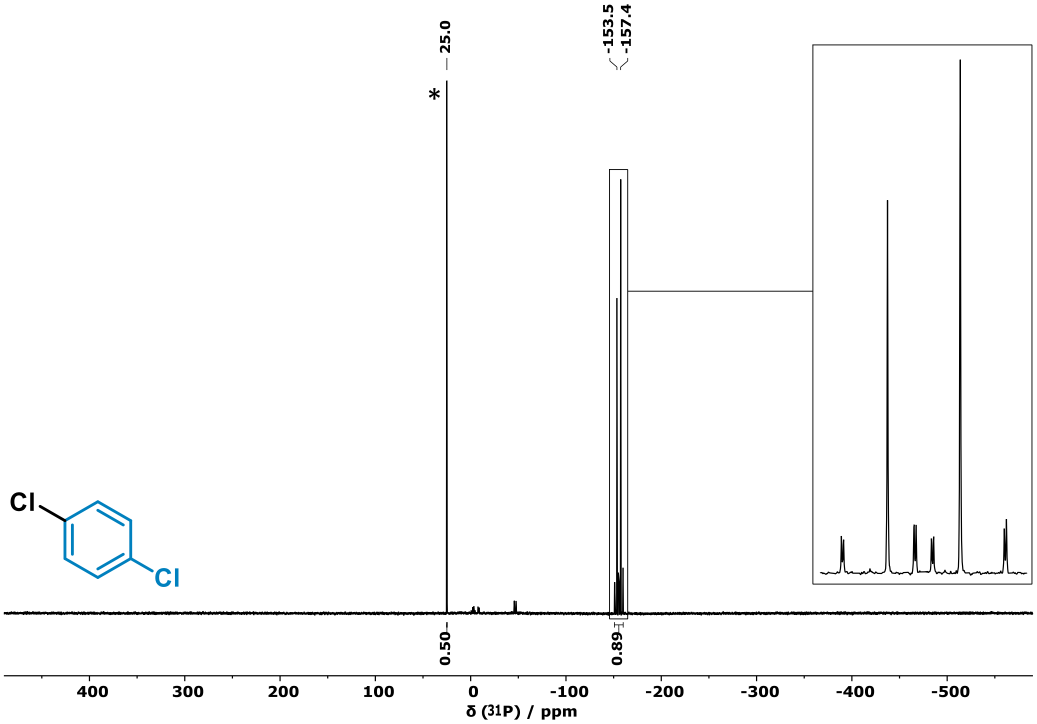


**Figure S35**: $^{31}P\{^{1}H\}$ NMR spectrum for the reaction of $(Bu_3Sn)_3P$ (0.04 mmol) and 1,4-dichlorobenzene with KHMDS in toluene. * marks the internal standard $Ph_3PO$ (0.02 mmol).

## S6.15 Reaction with 1-chloro-2-fluorobenzene

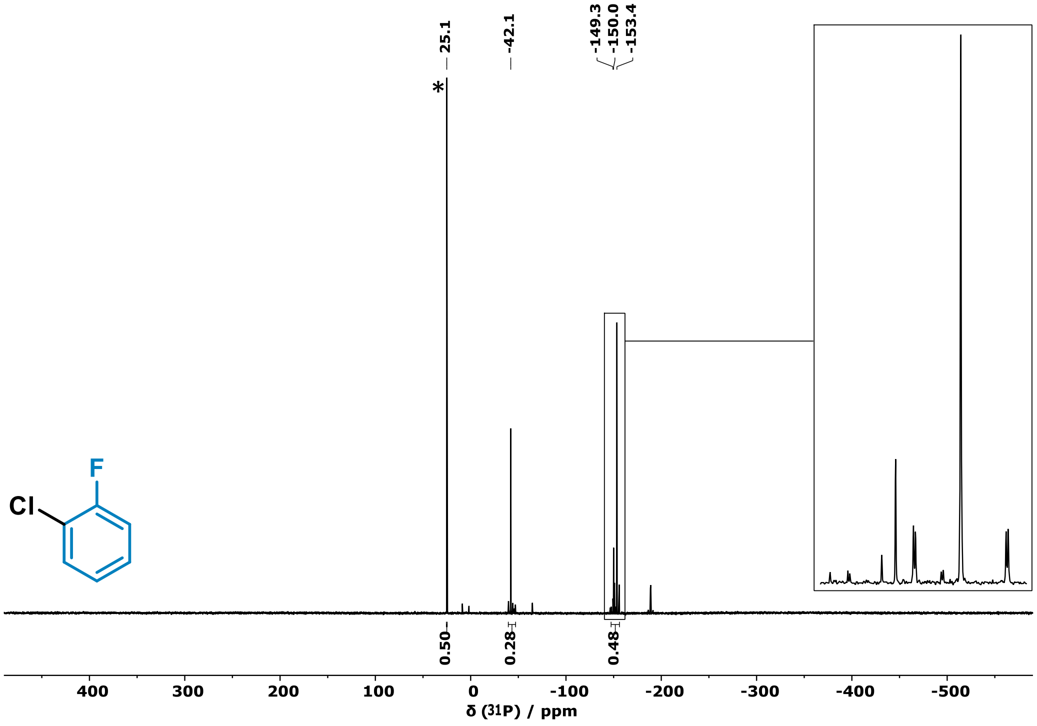


**Figure S36**: $^{31}P\{^{1}H\}$ NMR spectrum for the reaction of $(Bu_3Sn)_3P$ (0.04 mmol) and 1-chloro-2-fluorobenzene with KHMDS in toluene. * marks the internal standard $Ph_3PO$ (0.02 mmol).

## S6.16 Reaction with 1-chloro-4-iodobenzene

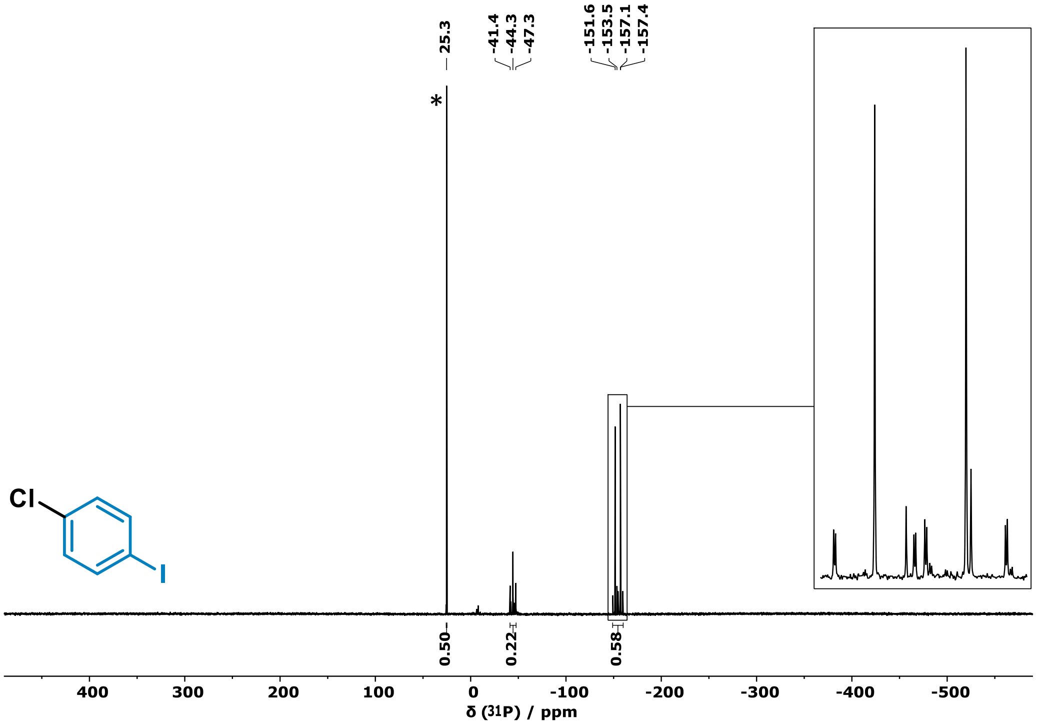


**Figure S37**: $^{31}P\{^{1}H\}$ NMR spectrum for the reaction of $(Bu_3Sn)_3P$ (0.04 mmol) and 1-chloro-4-iodobenzene with KHMDS in toluene. * marks the internal standard $Ph_3PO$ (0.02 mmol).

## S6.17 Reaction with 4-chloroanisole

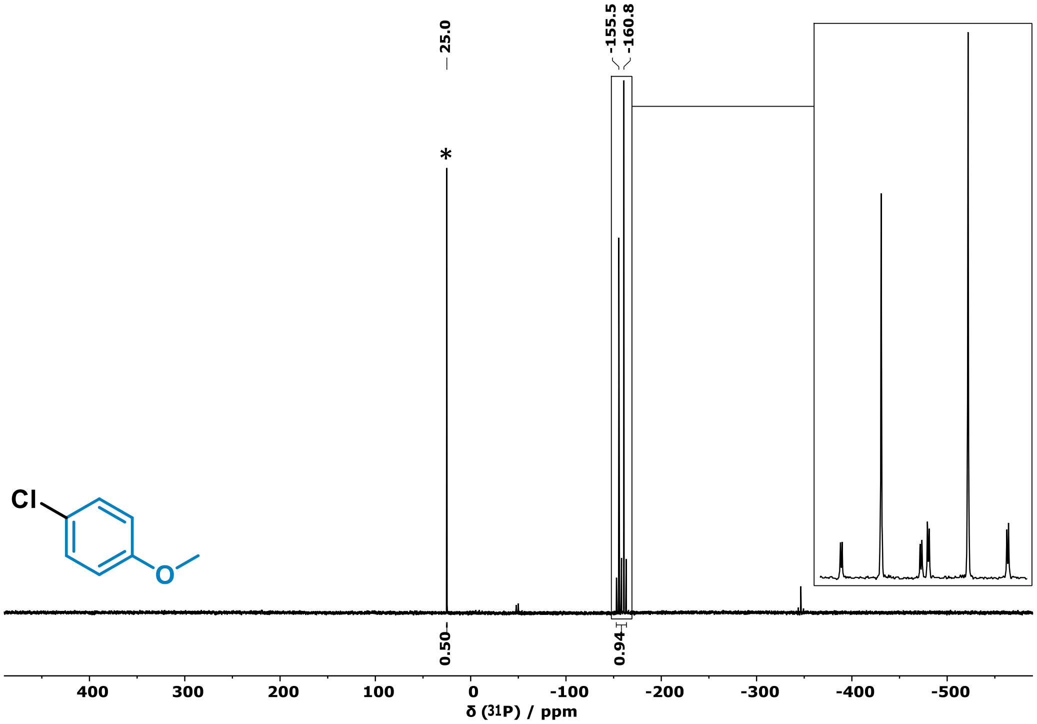


**Figure S38**: $^{31}P\{^{1}H\}$ NMR spectrum for the reaction of $(Bu_3Sn)_3P$ (0.04 mmol) and 4-chloroanisole with KHMDS in toluene. * marks the internal standard $Ph_3PO$ (0.02 mmol).

## S6.18 Reaction with 3-chloroanisole

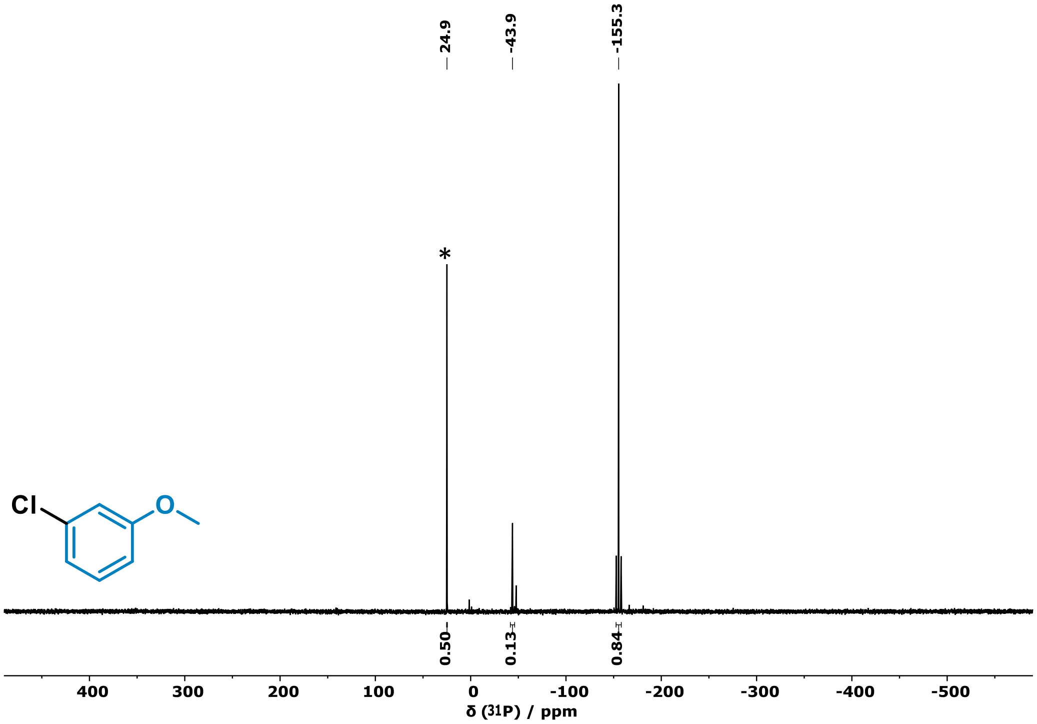


**Figure S39**: $^{31}P\{^{1}H\}$ NMR spectrum for the reaction of $(Bu_3Sn)_3P$ (0.04 mmol) and 3-chloroanisole with KHMDS in toluene. * marks the internal standard $Ph_3PO$ (0.02 mmol).

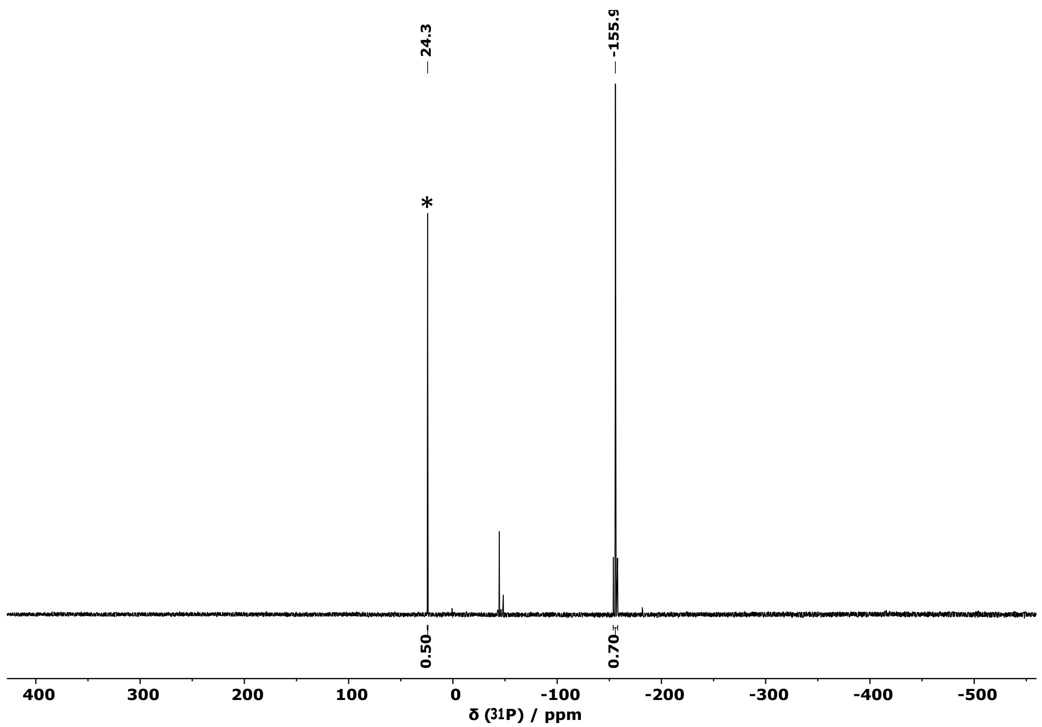


**Figure S40**: Quantitative $^{31}P\{^1H\}$ NMR (zgig30) spectrum for the reaction of $(Bu_3Sn)_3P$ (0.04 mmol) and 3-chloranisole with KHMDS in toluene. * marks the internal standard $Ph_3PO$ (0.02 mmol).

## S6.19 Reaction with 2-chloroanisole

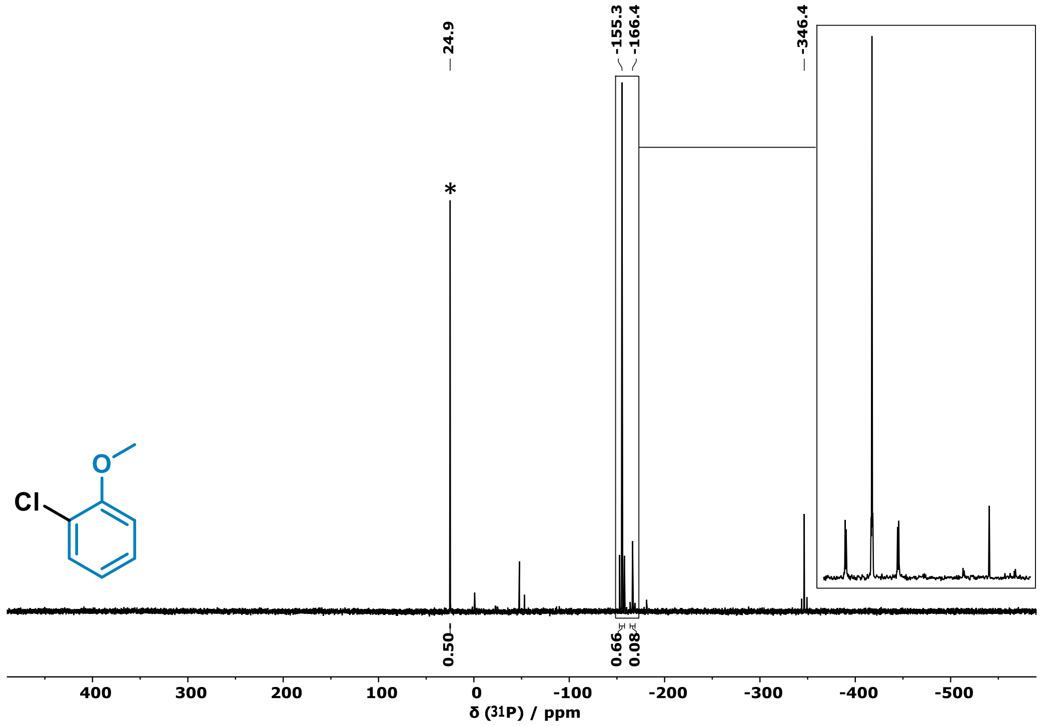


**Figure S41**: $^{31}P\{^1H\}$ NMR spectrum for the reaction of $(Bu_3Sn)_3P$ (0.04 mmol) and 2-chloroanisole with KHMDS in toluene. * marks the internal standard $Ph_3PO$ (0.02 mmol).

## S6.20 Reaction with 4-chlorothioanisole

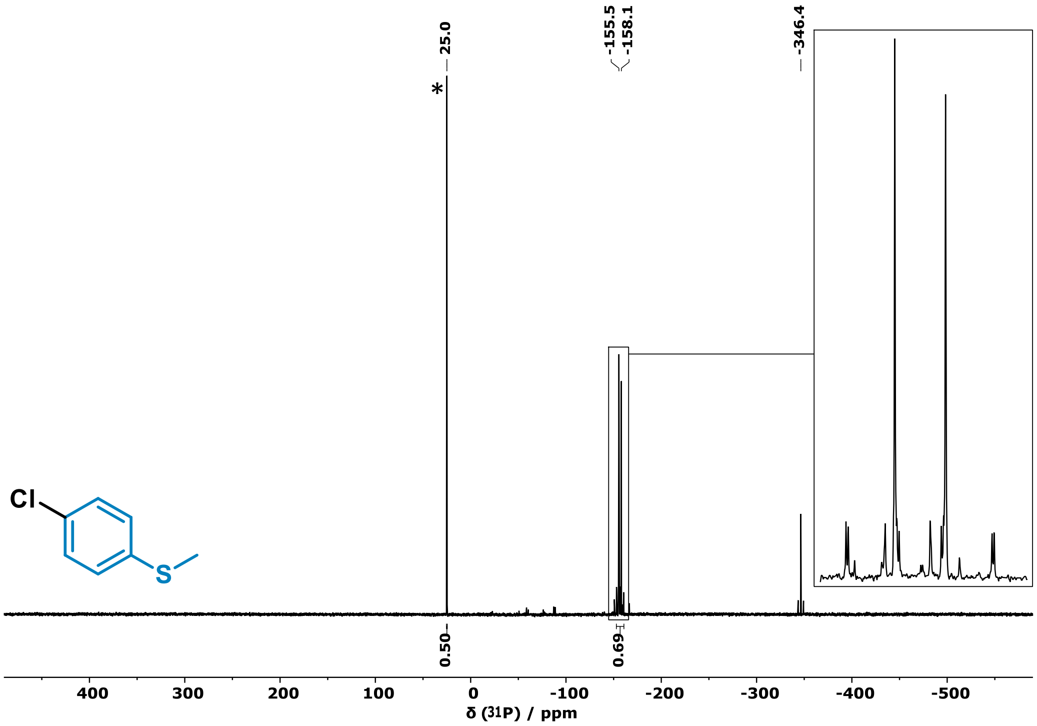


**Figure S42**: $^{31}P\{^{1}H\}$ NMR spectrum for the reaction of $(Bu_3Sn)_3P$ (0.04 mmol) and 4-chlorothioanisol with KHMDS in toluene. * marks the internal standard $Ph_3PO$ (0.02 mmol).

## S6.21 Reaction with 4-chlorobenzonitrile

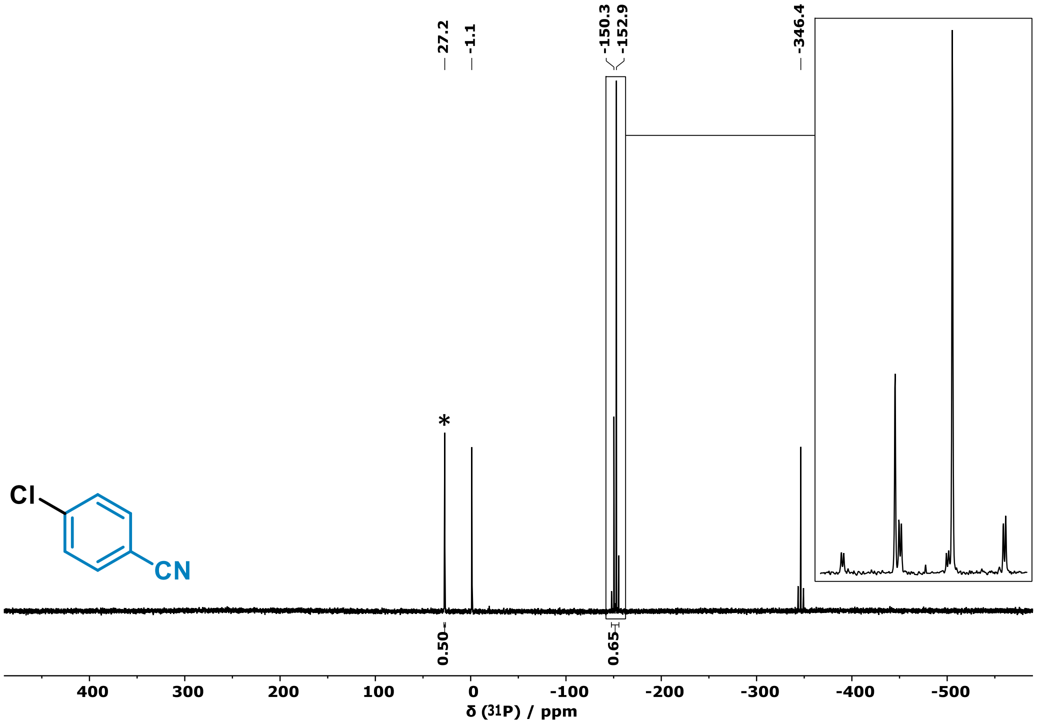


**Figure S43**: $^{31}P\{^{1}H\}$ NMR spectrum for the reaction of $(Bu_3Sn)_3P$ (0.04 mmol) and 4-chlorobenzonitrile with KHMDS in toluene. * marks the internal standard $Ph_3PO$ (0.02 mmol).

## S6.22 Reaction with 1-chloro-2-(trifluoromethyl)benzene

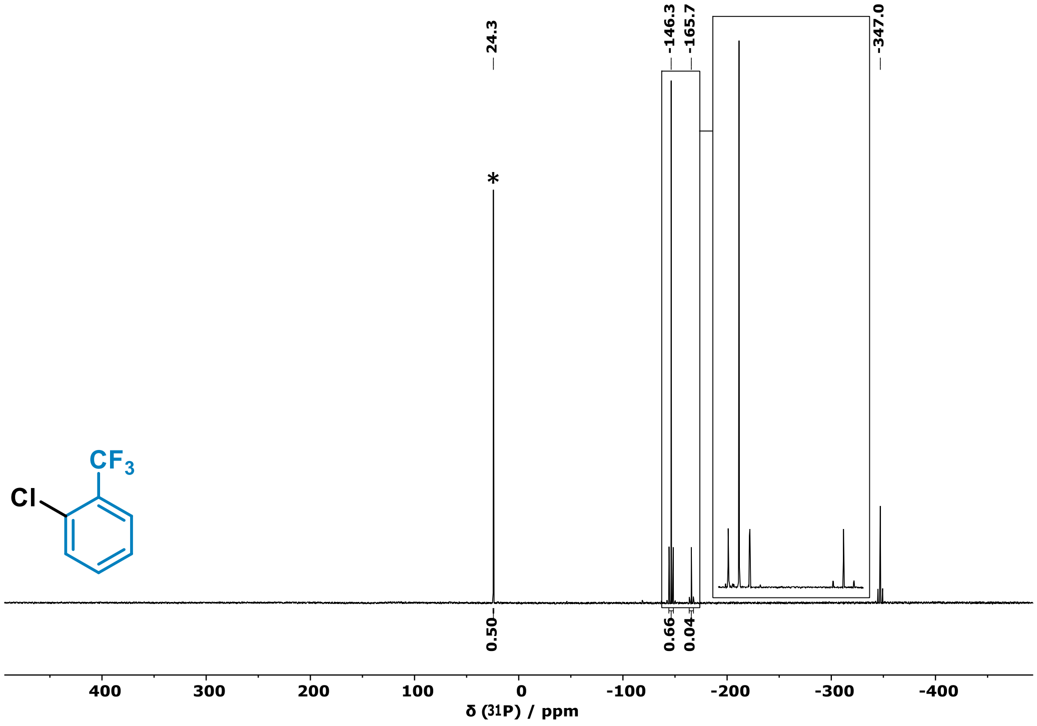


**Figure S44**: $^{31}P\{^{1}H\}$ NMR spectrum for the reaction of $(Bu_3Sn)_3P$ (0.04 mmol) and 1-chloro-2-(trifluoromethyl)-benzene with KHMDS in toluene. * marks the internal standard $Ph_3PO$ (0.02 mmol).

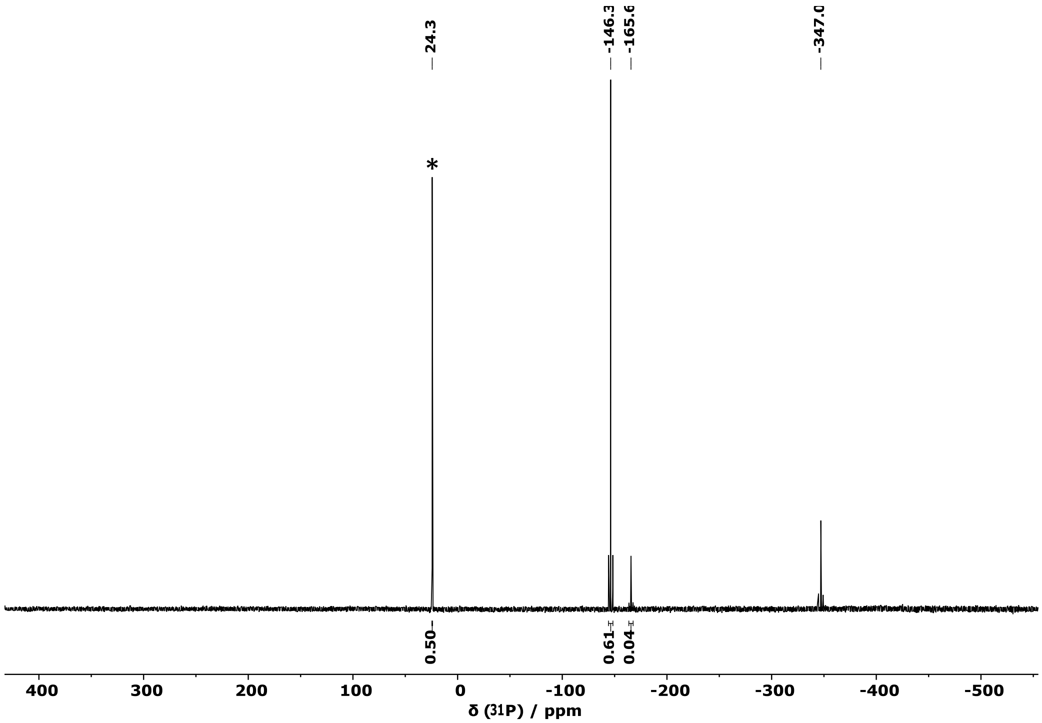


**Figure S45**: Quantitative $^{31}P\{^{1}H\}$ NMR (zgig30) spectrum for the reaction of $(Bu_3Sn)_3P$ (0.04 mmol) and 1-chloro-2-(trifluoromethyl)benzene with KHMDS in toluene. * marks the internal standard $Ph_3PO$ (0.02 mmol).

## S6.23 Reaction with benzyl chloride

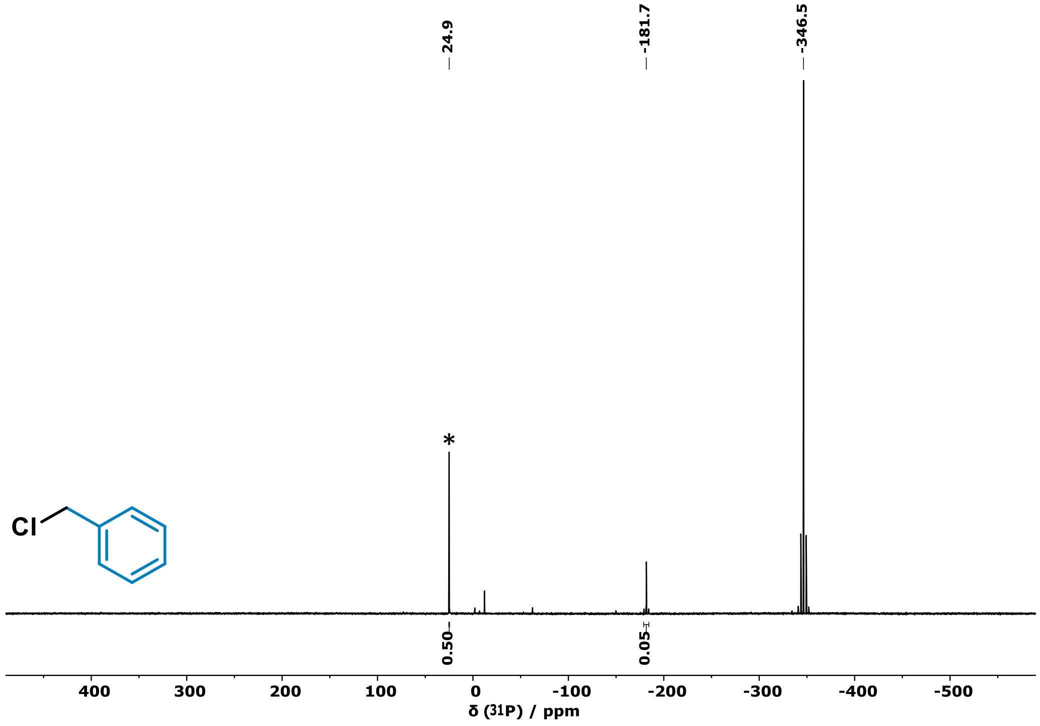


**Figure S46**: $^{31}P\{^{1}H\}$ NMR spectrum for the reaction of $(Bu_3Sn)_3P$ (0.04 mmol) and benzyl chloride with KHMDS in toluene. * marks the internal standard $Ph_3PO$ (0.02 mmol).

## S6.24 Reaction with bromoethane

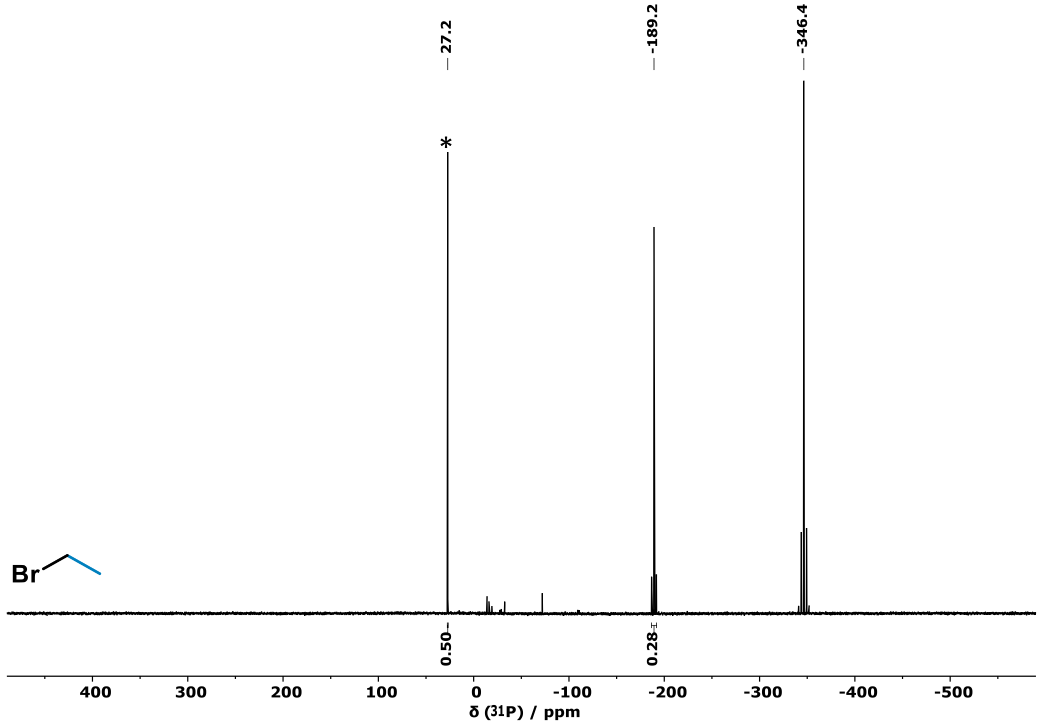


**Figure S47**: $^{31}P\{^{1}H\}$ NMR spectrum for the reaction of $(Bu_3Sn)_3P$ (0.04 mmol) and bromoethane with KHMDS in toluene. * marks the internal standard $Ph_3PO$ (0.02 mmol).

## S6.25 Reaction with 1-bromo-2,4,4-trimethylpentane

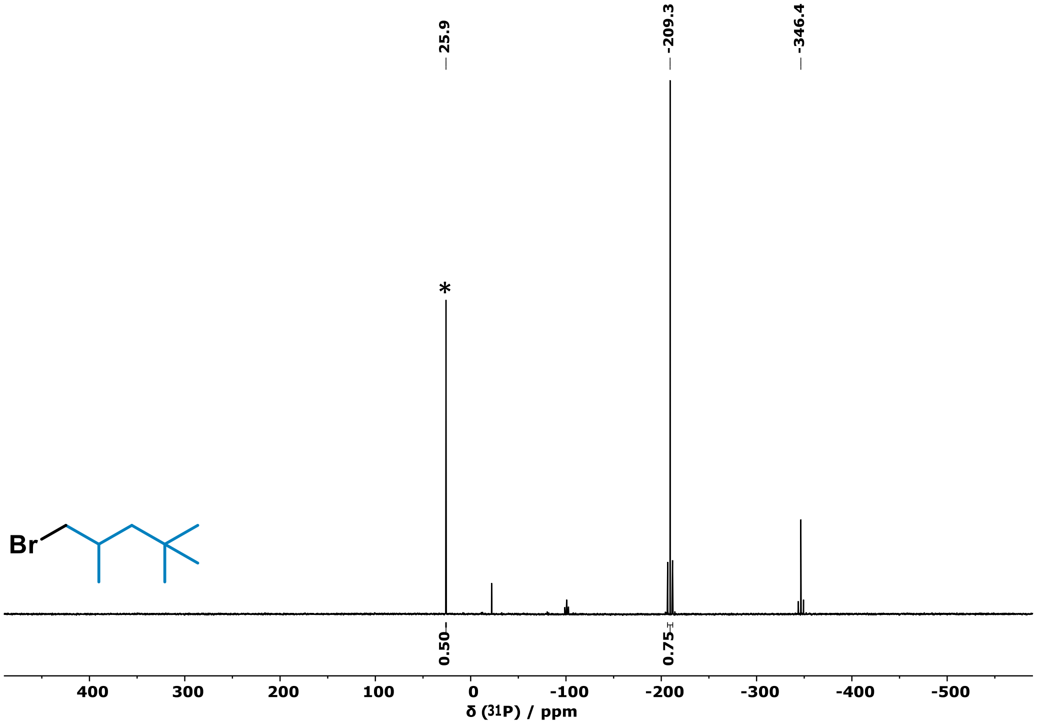


**Figure S48**: $^{31}P\{^{1}H\}$ NMR spectrum for the reaction of $(Bu_3Sn)_3P$ (0.04 mmol) and 1-bromo-2,4,4-trimethylpentane with KHMDS in toluene. * marks the internal standard $Ph_3PO$ (0.02 mmol).

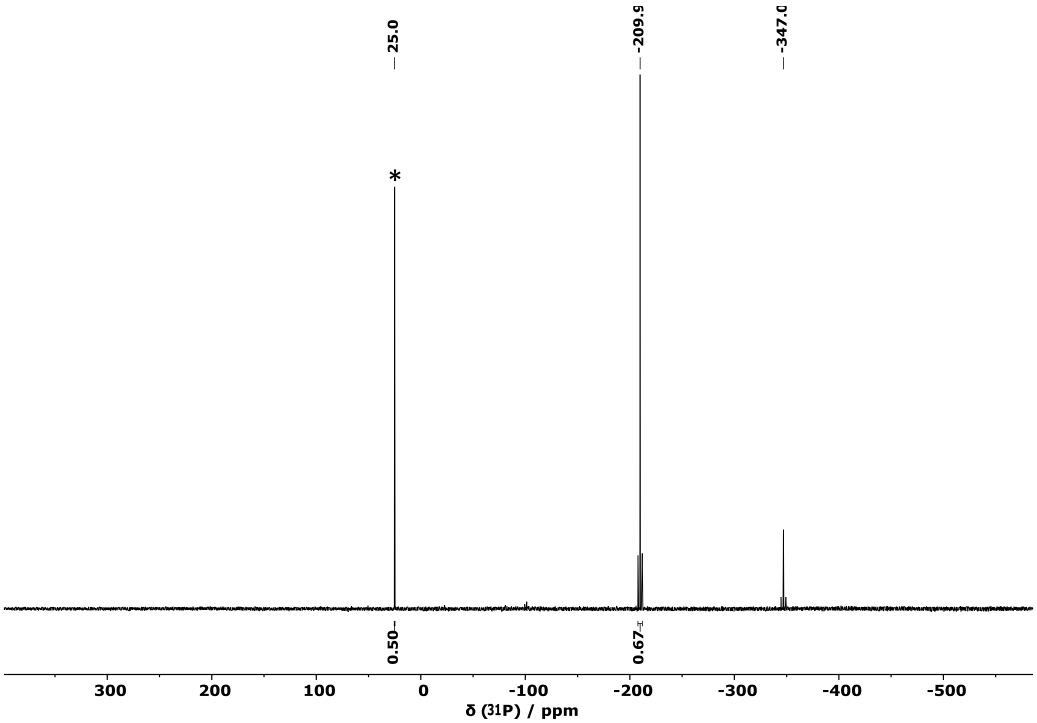


**Figure S49**: Quantitative $^{31}P\{^{1}H\}$ NMR (zgig30) spectrum for the reaction of $(Bu_3Sn)_3P$ (0.04 mmol) and 1-bromo-2,4,4-trimethylpentane with KHMDS in toluene. * marks the internal standard $Ph_3PO$ (0.02 mmol).

## S6.26 Reaction with 3-(bromomethyl)heptane

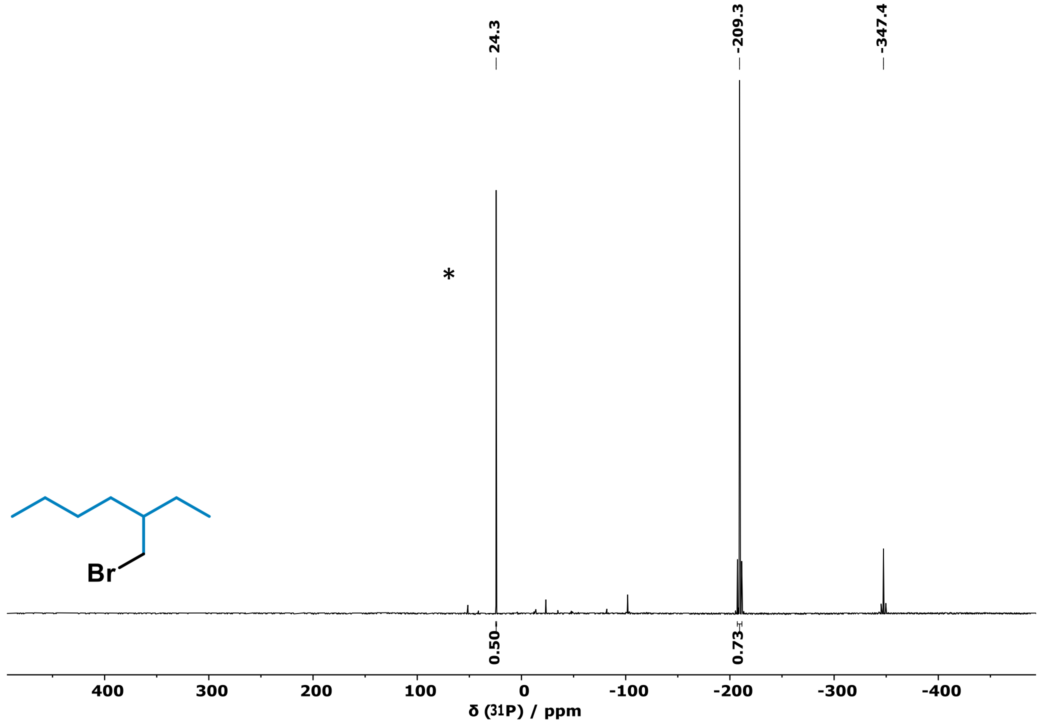


**Figure S50**: $^{31}P\{^{1}H\}$ NMR spectrum for the reaction of $(Bu_3Sn)_3P$ (0.04 mmol) and 3-(bromomethyl)heptane with KHMDS in toluene. * marks the internal standard $Ph_3PO$ (0.02 mmol).

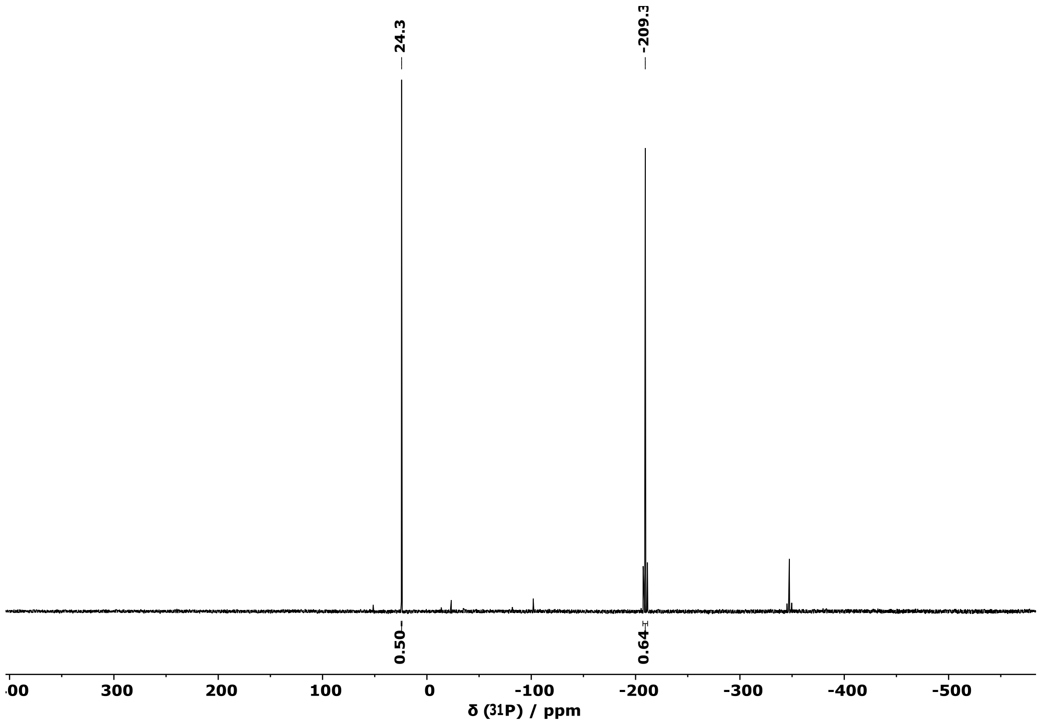


**Figure S51**: Quantitative $^{31}P\{^{1}H\}$ NMR (zgig30) spectrum for the reaction of $(Bu_3Sn)_3P$ (0.04 mmol) and 3-(bromomethyl)heptane with KHMDS in toluene. * marks the internal standard $Ph_3PO$ (0.02 mmol).

## S6.27 Reaction with 3-(chloromethyl)heptane

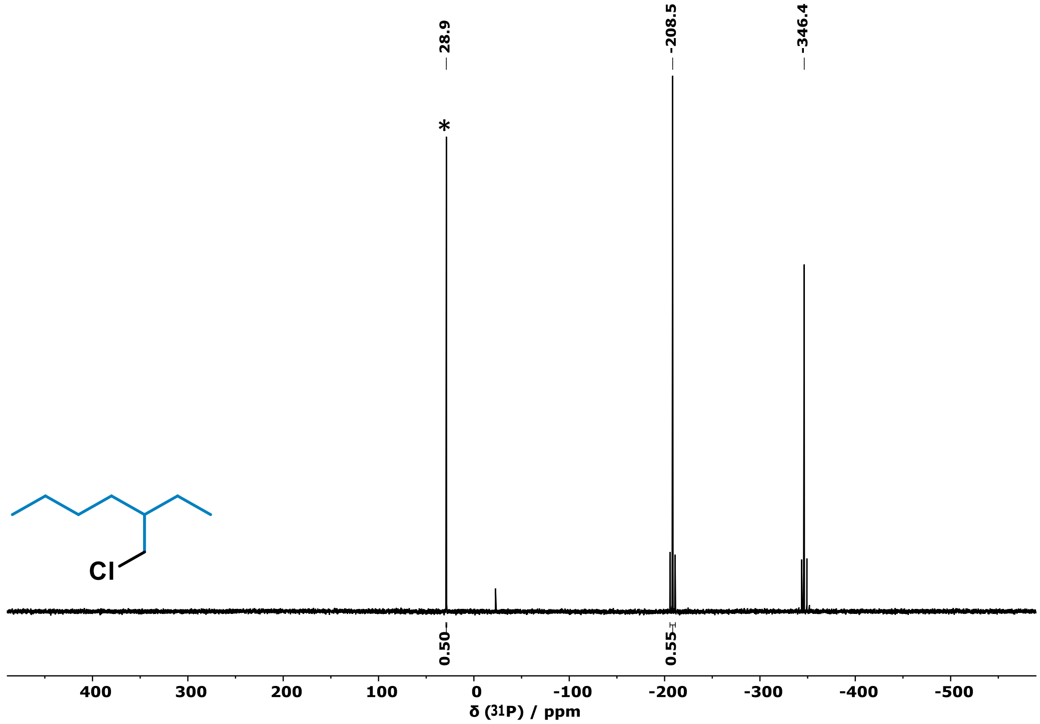


**Figure S52**: $^{31}P\{^{1}H\}$ NMR spectrum for the reaction of $(Bu_3Sn)_3P$ (0.04 mmol) and 3-(chloromethyl)heptane with KHMDS in toluene. * marks the internal standard $Ph_3PO$ (0.02 mmol).

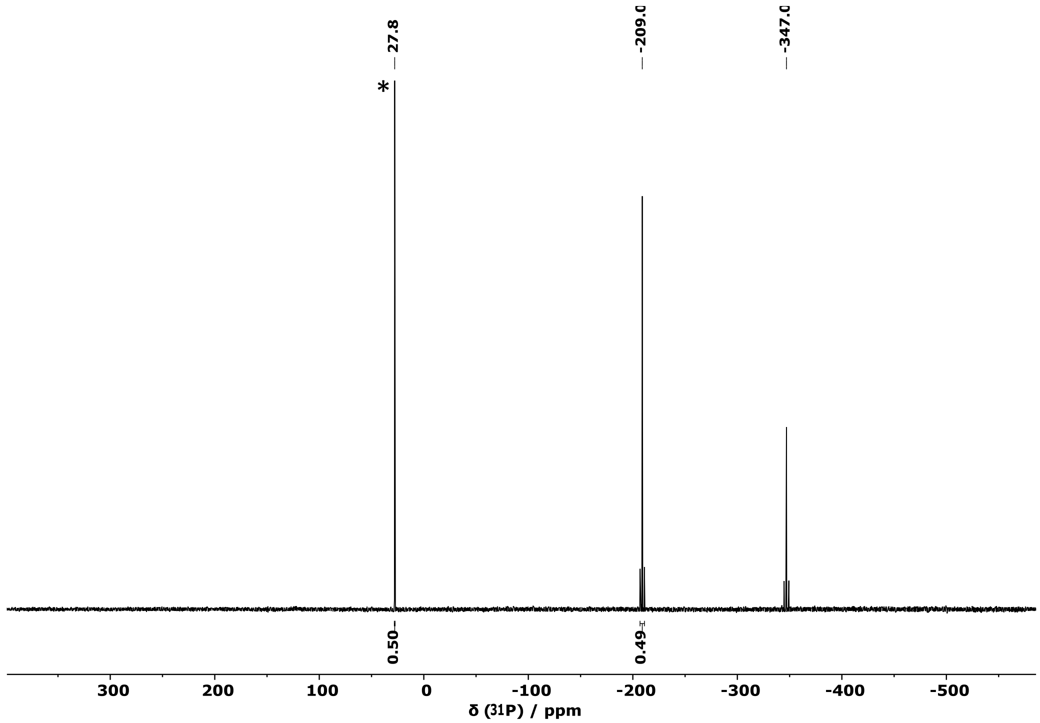


**Figure S53**: Quantitative $^{31}P\{^{1}H\}$ NMR (zgig30) spectrum for the reaction of $(Bu_3Sn)_3P$ (0.04 mmol) and 3-(chloromethyl)heptane with KHMDS in toluene. * marks the internal standard $Ph_3PO$ (0.02 mmol).

## S6.28 Reaction with 1-chloro-2,4,4-trimethylpentane

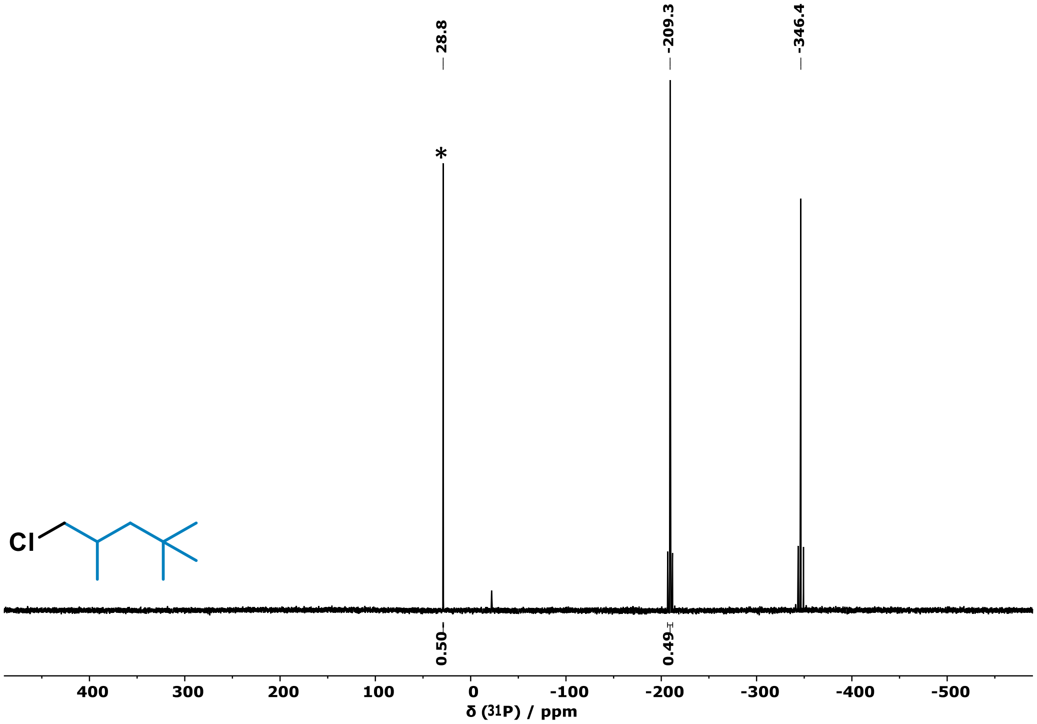


**Figure S54**: $^{31}P\{^1H\}$ NMR spectrum for the reaction of $(Bu_3Sn)_3P$ (0.04 mmol) and 1-chloro-2,4,4-trimethylpentane with KHMDS in toluene. * marks the internal standard $Ph_3PO$ (0.02 mmol).

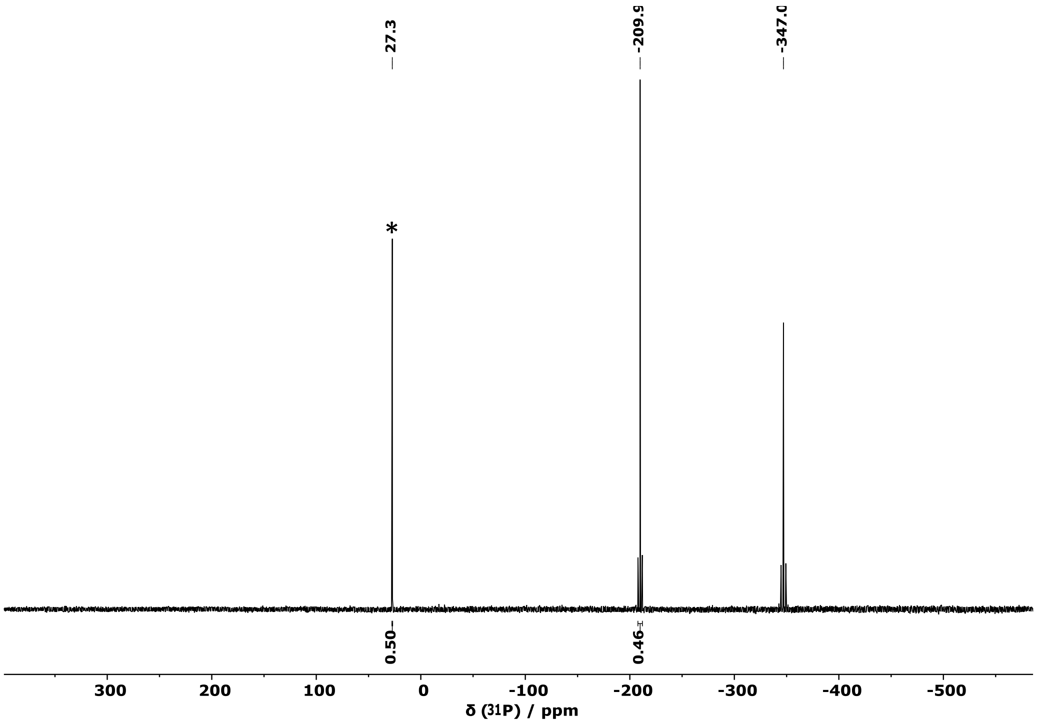


**Figure S55**: Quantitative $^{31}P\{^1H\}$ NMR (zgig30) spectrum for the reaction of $(Bu_3Sn)_3P$ (0.04 mmol) and 1-chloro-2,4,4-trimethylpentane with KHMDS in toluene. * marks the internal standard $Ph_3PO$ (0.02 mmol).

## S6.29 Reaction with 1,3-dichloropropane

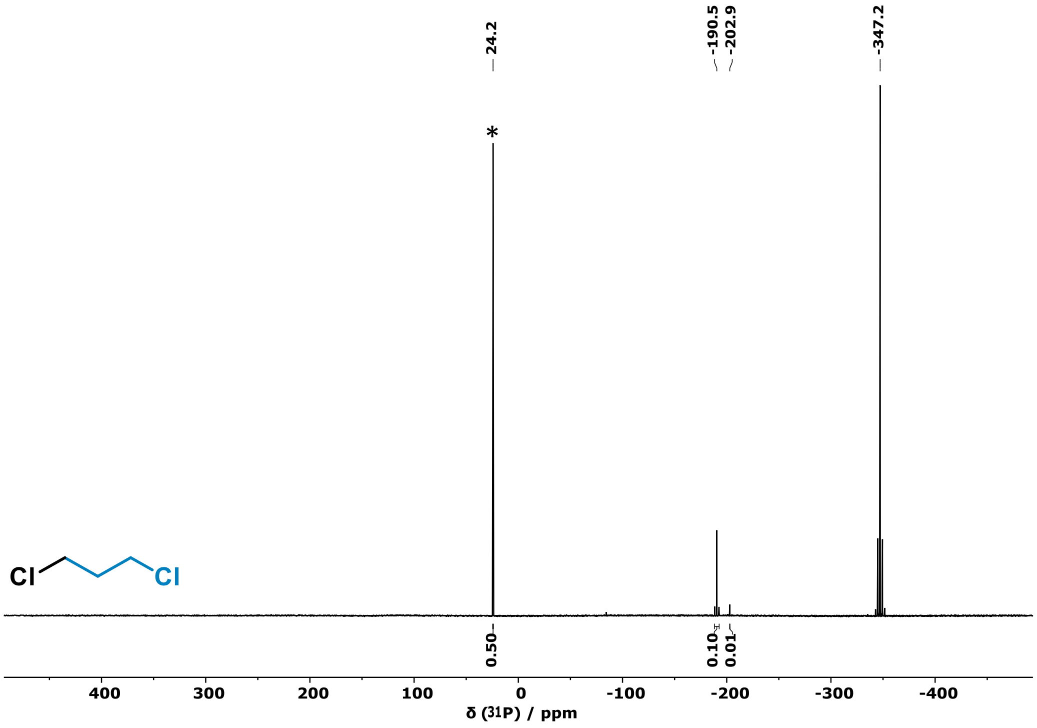


**Figure S56**: $^{31}P\{^{1}H\}$ NMR spectrum for the reaction of $(Bu_3Sn)_3P$ (0.04 mmol) and 1,3-dichloropropane with KHMDS in toluene. * marks the internal standard $Ph_3PO$ (0.02 mmol).

## S6.30 Reaction with 2-chloropropane

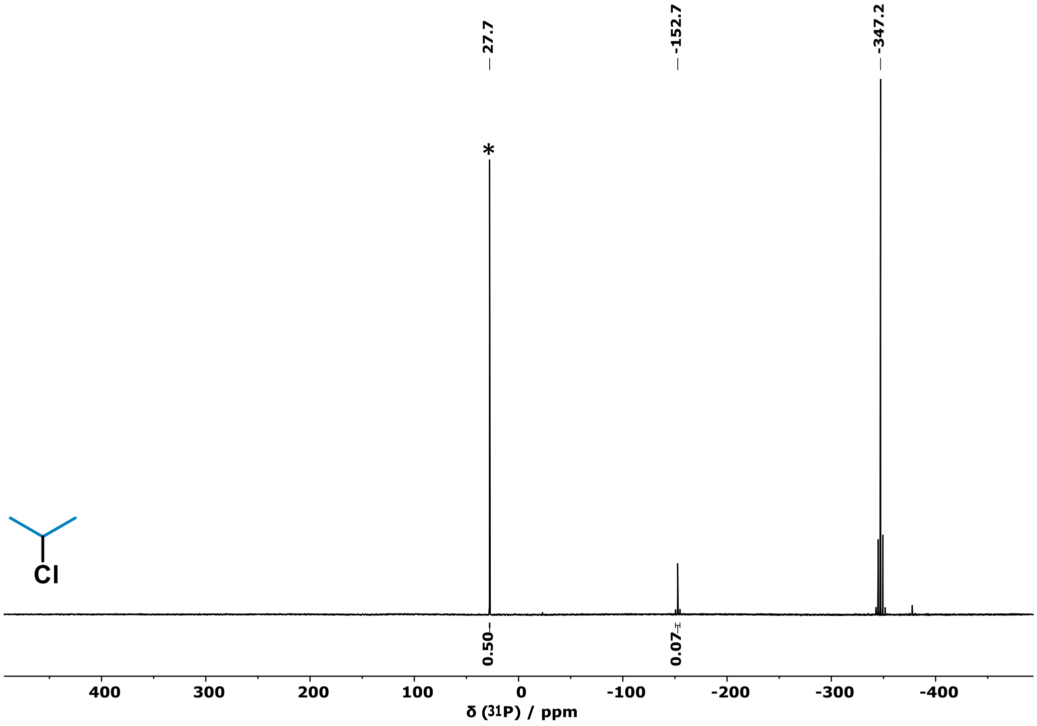


**Figure S57**: $^{31}P\{^{1}H\}$ NMR spectrum for the reaction of $(Bu_3Sn)_3P$ (0.04 mmol) and 2-chloropropane with KHMDS in toluene. * marks the internal standard $Ph_3PO$ (0.02 mmol).

# S7. Mechanistic investigations into the arylation of $(Bu_3Sn)_3P$

## S7.1 Reaction between $(Bu_3Sn)_3P$ and KHMDS

**Table S5**: Screening of the reaction of $(Bu_3Sn)_3P$ (0.04 mmol) and KHMDS in toluene at 100 °C for 18 h.[a]

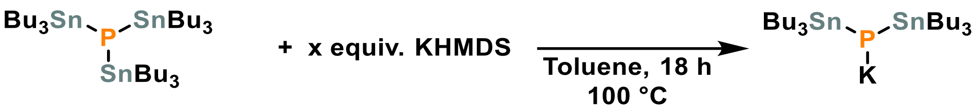


| Entry | Equivalents (x, per P) | Full conv. of $(Bu_3Sn)_3P$?[b] | Conversion towards product (%)[c] |
|---|---|---|---|
| 1 | 1 | ✘ | 4 |
| 2 | 1.5 | ✘ | 12 |
| 3 | 2 | ✘ | 16 |
| 4 | 3 | ✘ | 28 |
| 5 | 4 | ✘ | 32 |
| 6 | 5 | ✘ | 39 |

[a] The procedure was modified to use the corresponding reaction conditions. [b] Full conversion of $(Bu_3Sn)_3P$ was assessed by $^{31}P\{^1H\}$ NMR spectroscopy and the disappearance of the corresponding peak of $(Bu_3Sn)_3P$ at around −346.5 ppm. [c] Conversions towards the product were assessed by $^{31}P\{^1H\}$ NMR spectroscopy of the reaction and integrating the peaks of $(Bu_3Sn)_3P$ relative to the product.

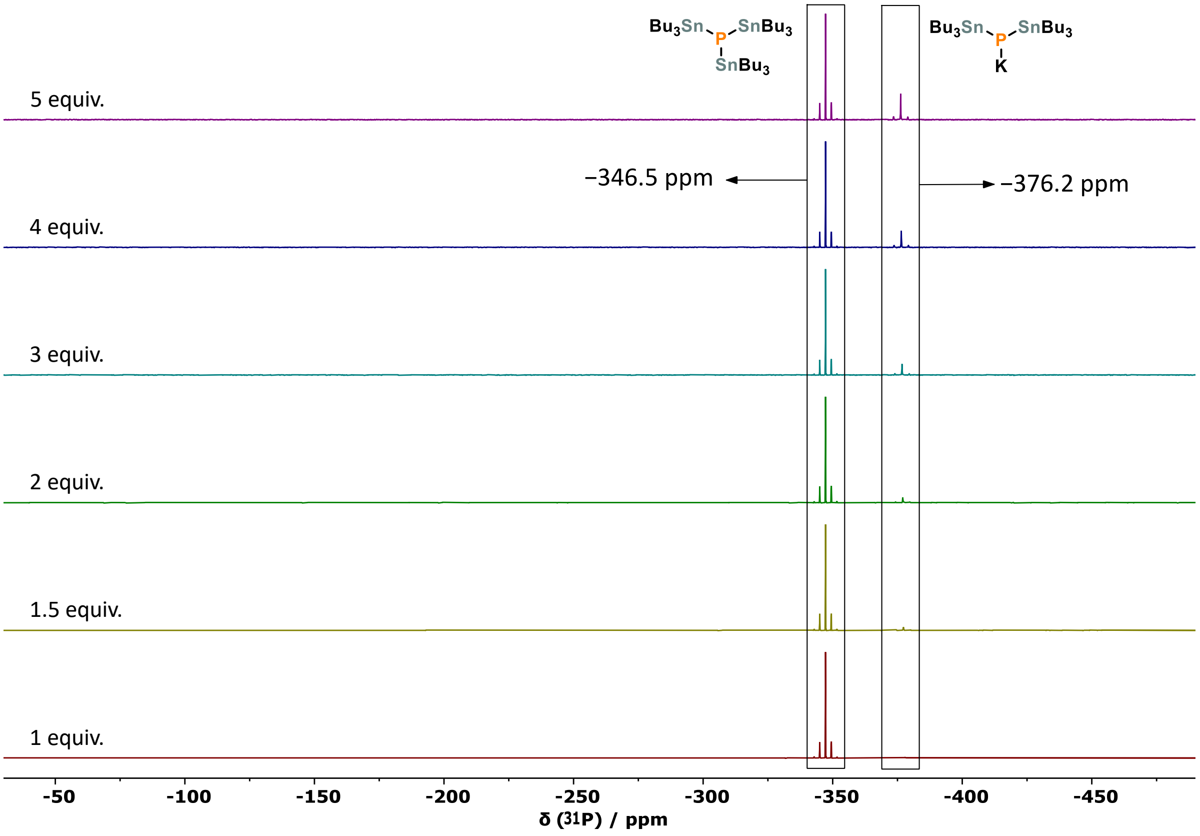


**Figure S58**: $^{31}P\{^1H\}$ NMR spectra of the reaction of $(Bu_3Sn)_3P$ with altering equivalents of KHMDS (1-5 equiv., bottom to top) in toluene at 100 °C for 18 h.

In the $^{31}P\{^1H\}$ NMR spectra, the ratio of the $^{117/119}Sn$ satellites to the main $^{31}P$ peak is approximately 0.2 for the newly formed signal. This indicates that two $Bu_3Sn$ groups are attached to the phosphorus atom. Furthermore, an upfield shift of the obtained product can be seen, which aligns with literature data, when comparing the chemical shifts for $P(SiMe_3)_3$ (−249.0 ppm) and $KP(SiMe_3)_2$ (−295.1 ppm), another "$P^{3-}$"-synthon and its related potassium phosphide, which is synthesised in the same manner, by using a strong, non-nucleophilic base.[7]

## S7.2 Reaction between the aryl halides and KHMDS

Most substituted aryl chlorides gave two isomeric primary phosphines (Figure S20, section S6). Furthermore, upon protonation using HCl as described in section 4, both products showed similar $^1J_{P\text{-}H}$ coupling constants, suggesting that two isomers are formed in these reactions. As a representative example, we can consider the case using 1-(*tert*-butyl)-4-chlorobenzene (for the $^{31}P\{^1H\}$ NMR spectrum before protonation, see Figure S29).

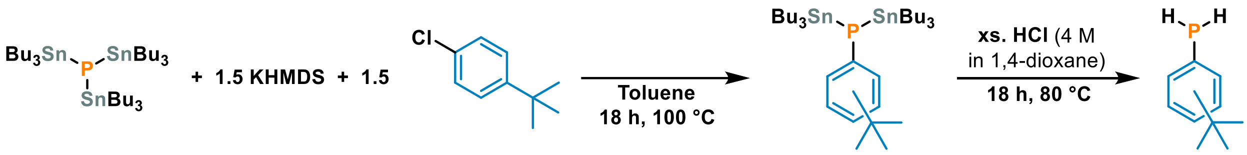


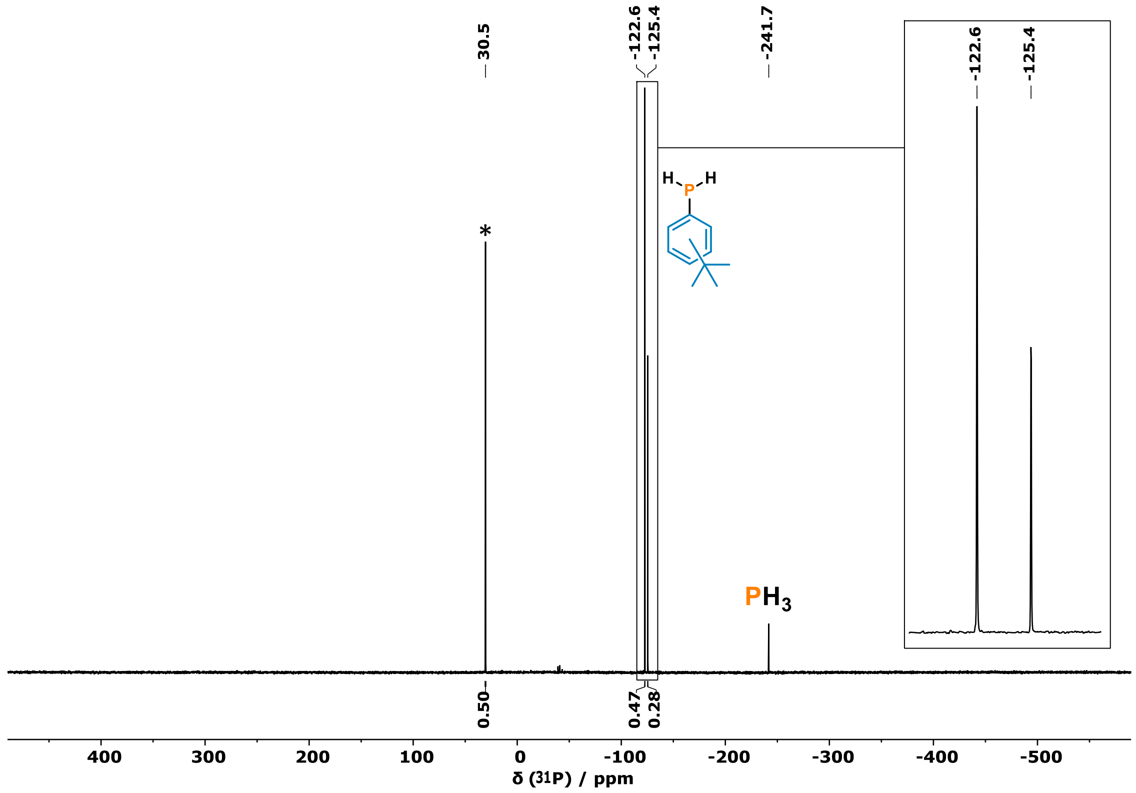


**Figure S59**: $^{31}P\{^1H\}$ NMR spectrum of the reaction of $(Bu_3Sn)_3P$ (0.04 mmol) with 1-(*tert*-butyl)-4-chlorobenzene (1.5 equiv.) and KHMDS (1.5 equiv.) in toluene at 100 °C for 18 h and subsequent protonation using an excess of HCl (4 M in 1,4-dioxane). * marks the internal standard $Ph_3PO$ (0.02 mmol).

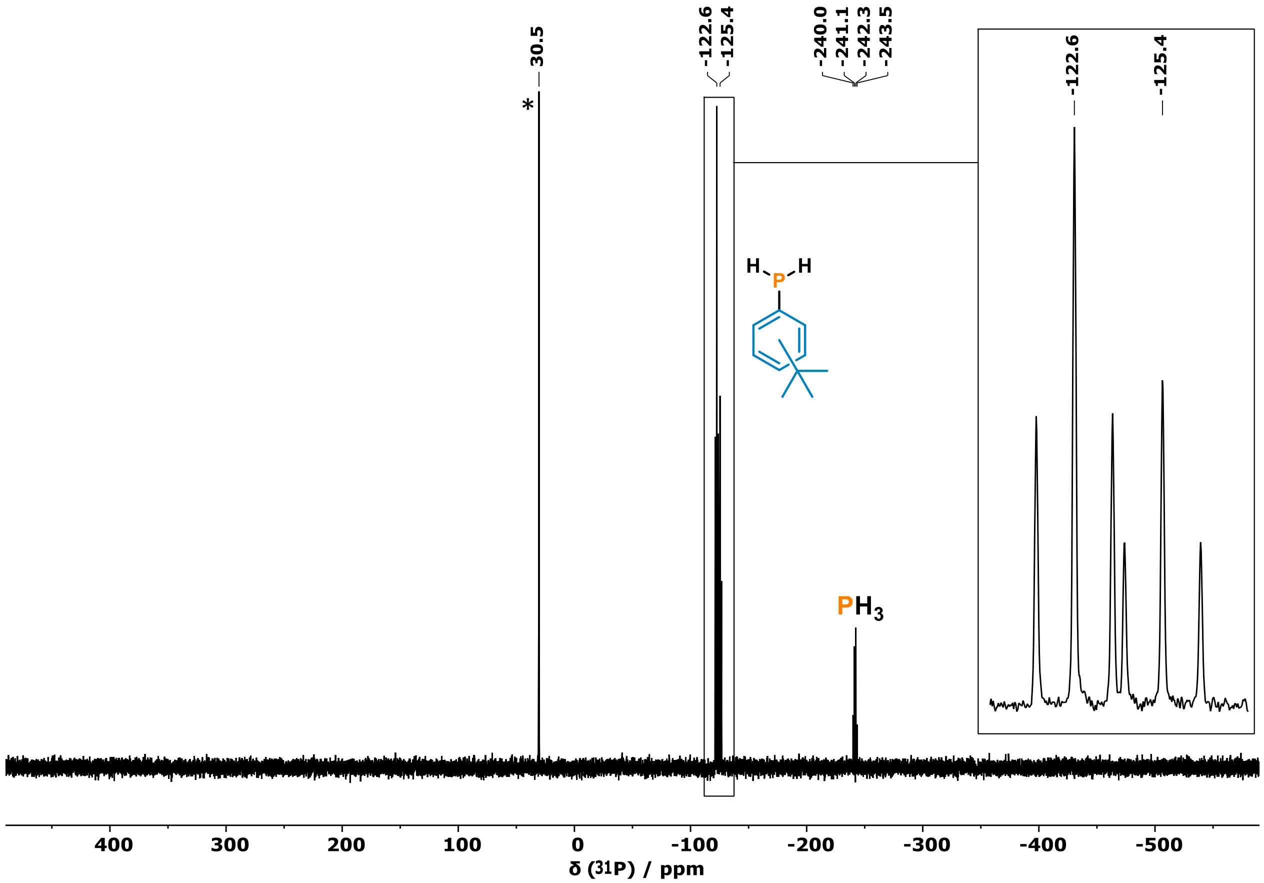


**Figure S60**: $^{31}P$ NMR spectrum of the reaction of $(Bu_3Sn)_3P$ (0.04 mmol) with 1-(*tert*-butyl)-4-chlorobenzene (1.5 equiv.) and KHMDS (1.5 equiv.) in toluene at 100 °C for 18 h and subsequent protonation using an excess of HCl (4 M in 1,4-dioxane). * marks the internal standard $Ph_3PO$ (0.02 mmol).

The signal in the NMR spectra at −241 ppm can be attributed to $PH_3$, which is formed by protonation of unreacted starting material $(Bu_3Sn)_3P$.[1] The two signals attributed to the products of the protonation show very similar chemical shifts. Furthermore, the $^{1}J_{P\text{-}H}$ couplings are identical for both products with a value of $^{1}J$ = 199 Hz. To further prove that these species are isomers, GC-MS measurements were performed on this reaction (Figure S54). Mass peaks at retention times (rt) of 5.2 min and 5.3 min can be assigned to the desired products with a m/z of 166.05. The fragmentation patterns of the corresponding mass peaks are also very similar, confirming that these are most likely isomers of the same product.

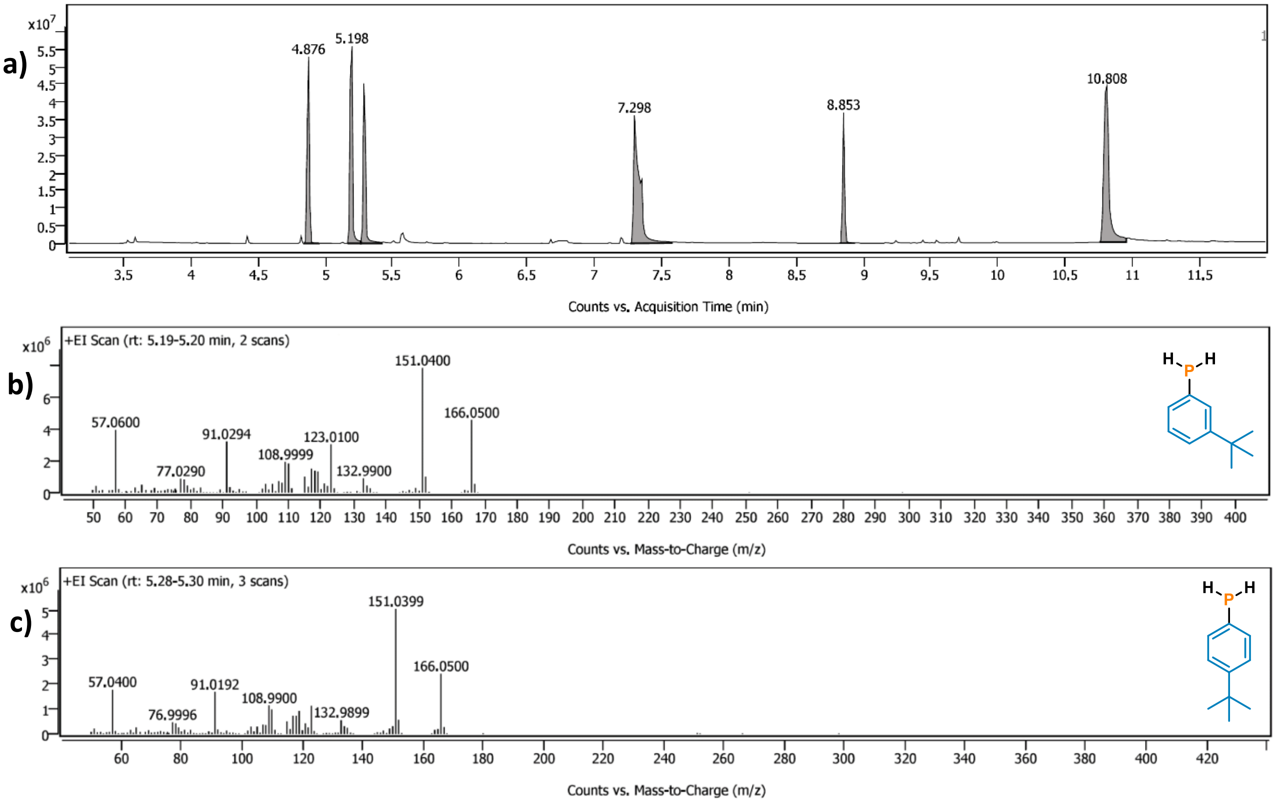


**Figure S61**: a) GC-MS chromatogram of the reaction of $(Bu_3Sn)_3P$ (0.04 mmol) with 1-(*tert*-butyl)-4-chlorobenzene (1.5 equiv.) and KHMDS (1.5 equiv.) in toluene at 100 °C for 18 h and subsequent protonation using an excess of HCl (4 M in 1,4-dioxane). b) Mass spectrum of one product isomer at the rt of 5.2 min. c) Mass spectrum of one product isomer at the rt of 5.3 min.

The reason for this observation can be seen when reacting KHMDS with chlorobenzene alone in toluene at 100 °C for 18 h. In the GC-MS chromatogram of this reaction, two peaks can be assigned to *N*,*N*-bis(trimethylsilyl)aniline and *N*,2-bis(trimethylsilyl)aniline, due to their slightly different fragmentation pattern, which is in alignment with literature for the similar reaction of bromobenzene and NaHMDS (Figure S62).[8] In accordance to these findings, it can be anticipated that the substitution at the aryl halide proceeds *via* a aryne intermediate featuring two adjacent carbons that are both viable sites for nucleophilic attack of $(Bu_3Sn)_3P$, resulting in two isomeric products (Figure S63).[8,9]

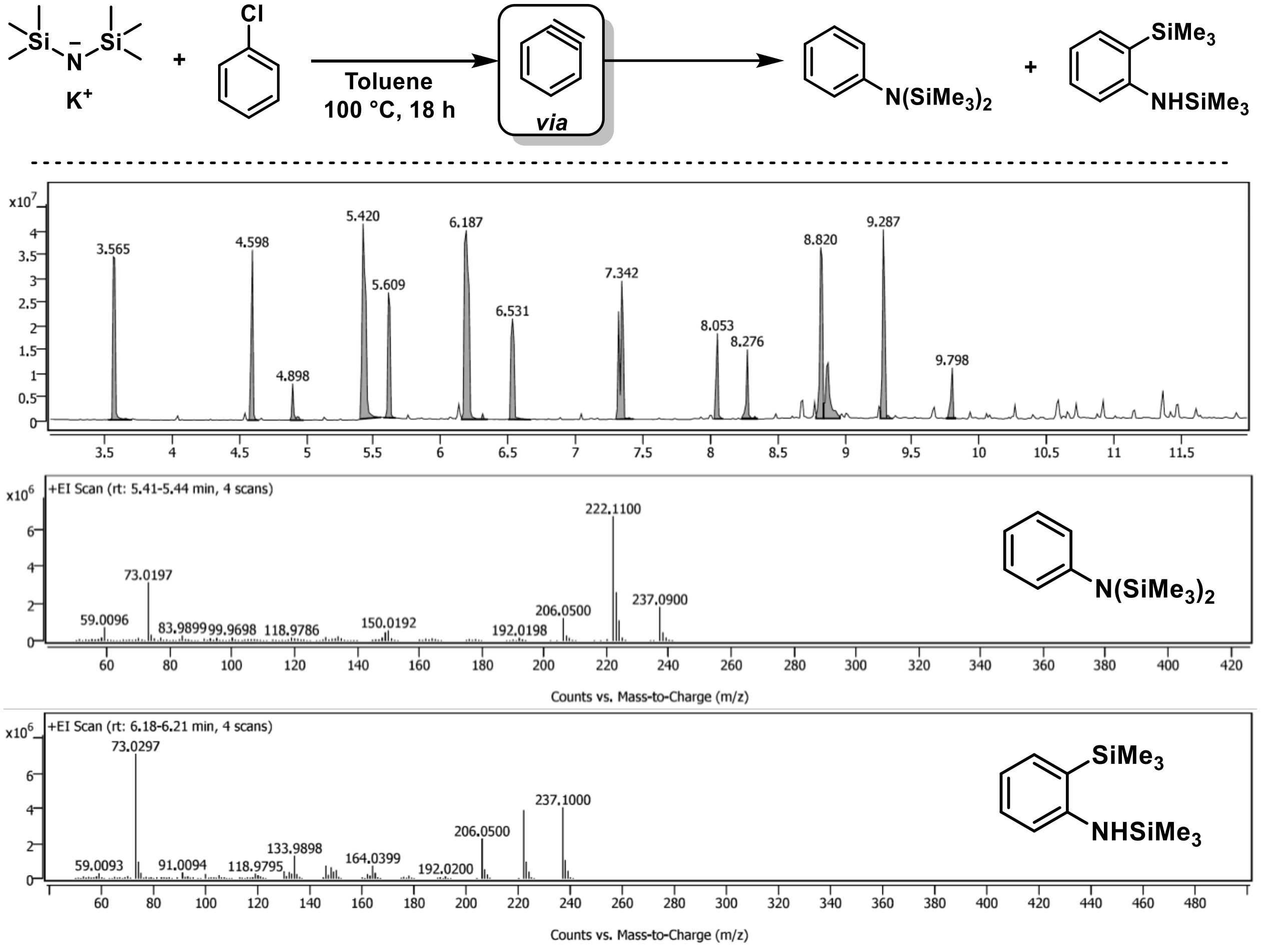


**Figure S62**: GC-MS chromatogram of the reaction of chlorobenzene (0.04 mmol) and KHMDS (1 equiv.) in toluene at 100 °C for 18 h (top) and mass spectra for selected products *N*,*N*-bis(trimethylsilyl)aniline (middle) and *N*,2-bis(trimethylsilyl)aniline (bottom).

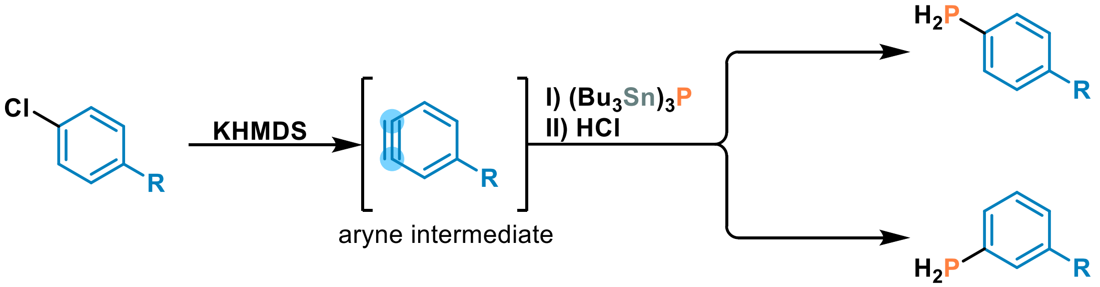


**Figure S63**: General pathways of the generation of isomeric primary phosphines *via* an aryne intermediate.

## S8. Structural investigation of reactions of $(Bu_3Sn)_3P$ and selected aryl chlorides

When conducting the arylation of $(Bu_3Sn)_3P$ with either 1-chloro-2-(trifluoromethyl)benzene or 3-chloroanisole as outlined in section 3, the $^{31}P\{^1H\}$ NMR spectrum reveals that only one product is selectively formed. According to the proposed mechanism detailed in section 7, which involves the dehydrohalogenation of aryl chlorides, two to three regioisomers could potentially form with 1-chloro-2-(trifluoromethyl)benzene or 3-chloroanisole, respectively (see Figure S64).

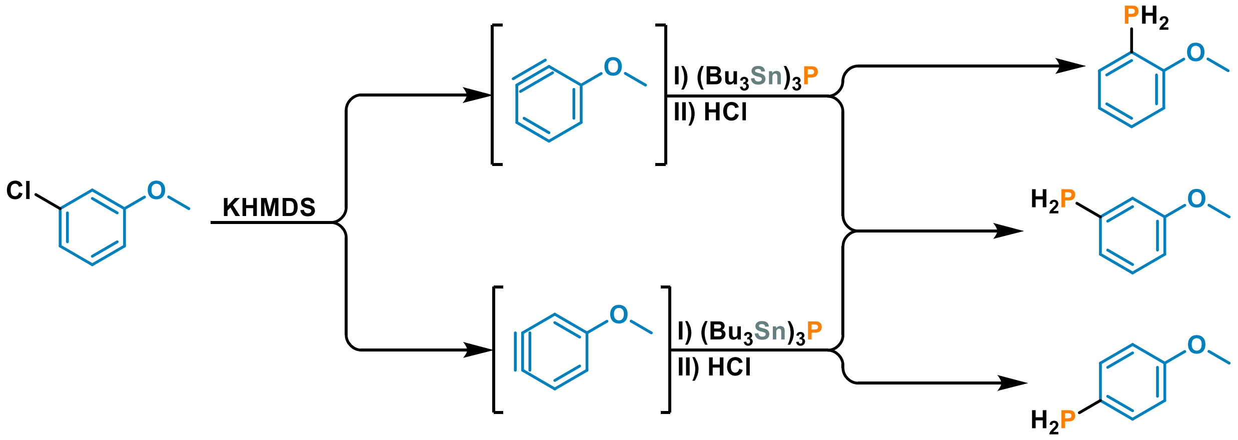


**Figure S64**: Possible pathways for the generation of three possible regioisomers for the reaction of $(Bu_3Sn)_3P$ with 3-chloroanisole.

Thus, efforts were made to determine which regioisomer was formed in the reactions. The $^{31}P\{^1H\}$ NMR spectrum of $(Bu_3Sn)_3P$ and 1-chloro-2-(trifluoromethyl)benzene reveals a signal at −123.5 ppm. In contrast, the $^1H$ NMR spectrum displays four signals in the aromatic region: two doublets and two triplets, with integrals 1:1:1:1. In the $^1H$-COSY spectrum, the doublet at 7.54 ppm does not correlate with any other $^1H$ signal. Additionally, the triplet at 7.45 ppm correlates only with the triplet at 7.18 ppm. Similarly, the doublet at 7.32 ppm correlates with the triplet at 7.18 ppm. This indicates that the *meta*-substituted phosphine is present, with $H^1$ corresponding to the doublet at 7.54 ppm, the triplet at 7.45 ppm linked to $H^4$, the doublet at 7.32 ppm assigned to $H^2$, and the triplet at 7.18 ppm representing proton $H^3$ (see Figure S65 for proton assignment).

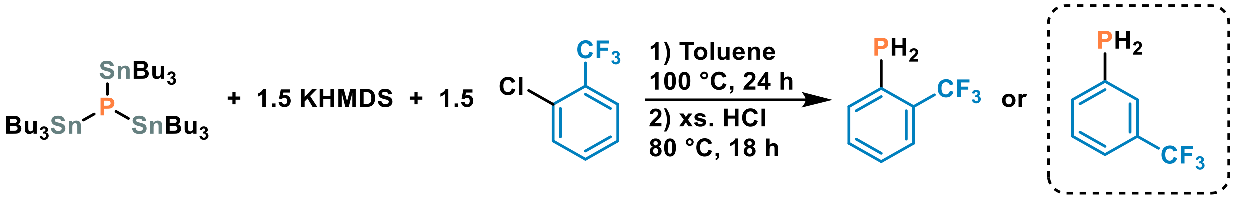


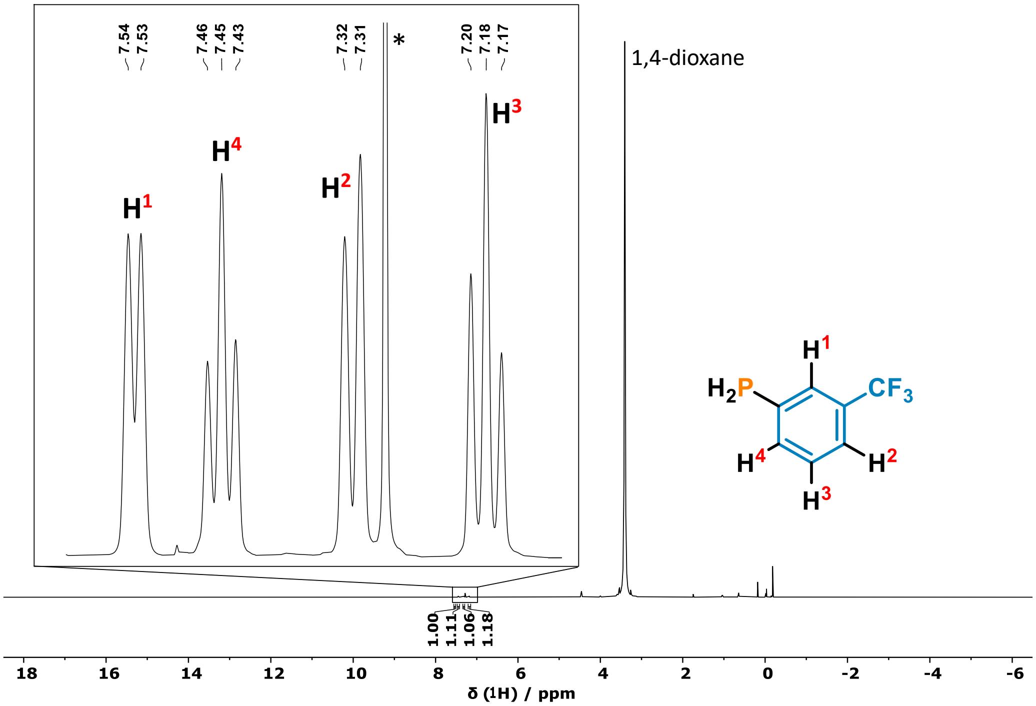


**Figure S65**: $^1$H NMR spectrum of the reaction of $(Bu_3Sn)_3P$ and 1-chloro-2-(trifluoromethyl)benzene and KHMDS and subsequent protonation, with proton assignment. * marks $C_6D_6$.

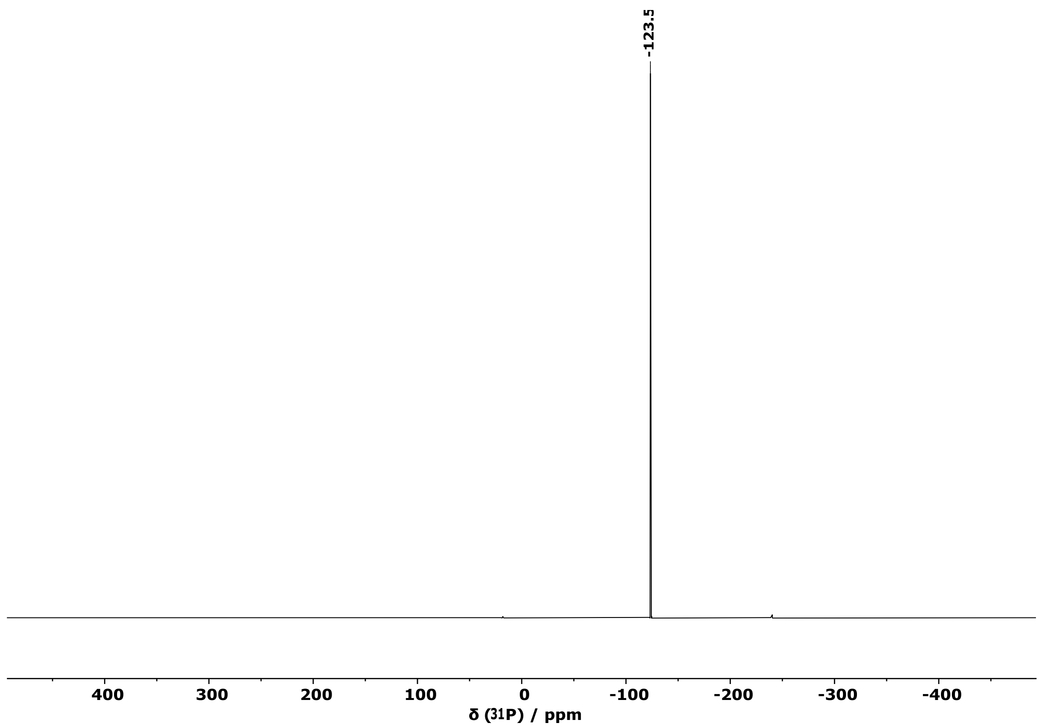


**Figure S66**: $^{31}P\{^1H\}$ NMR spectrum of the reaction of $(Bu_3Sn)_3P$ and 1-chloro-2-(trifluoromethyl)benzene and KHMDS and subsequent protonation.

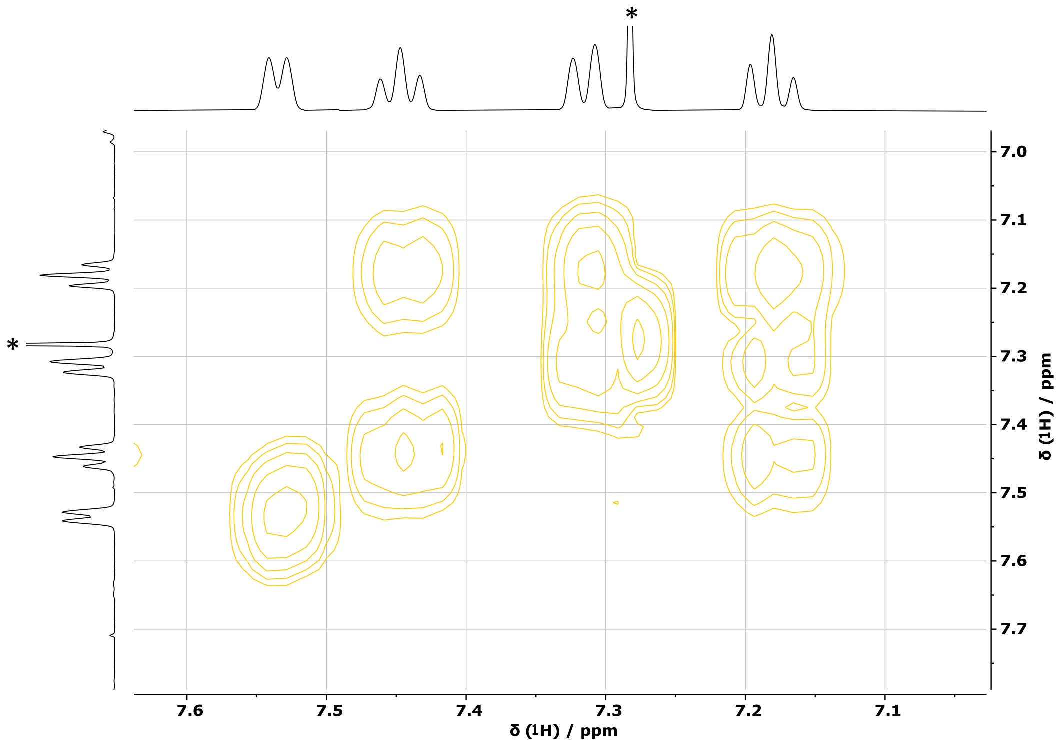


**Figure S67**: $^1H$-COSY NMR spectrum of the reaction of $(Bu_3Sn)_3P$ and 1-chloro-2-(trifluoromethyl)benzene and KHMDS and subsequent protonation. * marks $C_6D_6$.

Examining the reaction of $(Bu_3Sn)_3P$ with 3-chloroanisole, the $^{31}P\{^1H\}$ NMR spectrum again reveals one signal for the desired phosphine at −122.5 ppm. The $^1H$ NMR spectrum displays three signals with integrals of 1:2:1; however, the middle signal at 6.80 ppm is likely two signals superimposing. Therefore, since four distinct signals for the protons are present, the product is unlikely to have a *para*-configuration. Additionally, the literature reported $^{31}P\{^1H\}$ NMR signal for the *ortho*-substituted phosphine is at −140.0 ppm,[10] which is considerably more upfield shifted, indicating that the *ortho*-position is also unlikely to be applicable for the product here. Moreover, 2D-NMR experiments provide extra information supporting the presence of a *meta*-substituted phosphine. In the $^1H$-COSY NMR spectra, it can be observed that the doublet at 6.58 ppm does not correlate with the triplet at 6.80 ppm, and the triplet at 6.94 ppm does not correlating with the doublet at 6.77 ppm. In the $^1H$–$^{13}C$ HSQC NMR spectrum, the $^1H$ NMR doublet at 6.58 ppm correlates with the $^{13}C$ NMR signal at 113.6 ppm, which shows no $J_{C\text{–}P}$ coupling, suggesting that the $^1H$ NMR signal corresponds to proton $H^2$ and the $^{13}C$ NMR signal to $C^4$. Additionally, the $^1H$ NMR triplet at 6.80 ppm and the doublet at 6.77 ppm correlate with the $^{13}C$ NMR signals at 126.4 ppm and 119.3 ppm, respectively. Both $^{13}C$ NMR signals exhibit a $J_{C\text{–}P}$ coupling constant of 15.3 Hz for the former and 16.5 Hz for the latter, indicating $^2J$ couplings. Therefore, the $^{13}C$ NMR signal at 119.3 ppm corresponds to $C^2$, and the signal at 126.4 ppm corresponds to $C^6$, implying that the $^1H$ NMR doublet at 6.77 ppm is proton $H^1$ and the triplet at 6.80 ppm is proton $H^4$. Ultimately, the $^1H$ NMR triplet at 6.94 ppm correlates with the $^{13}C$ NMR signal at 129.1 ppm, which shows a $J_{C\text{–}P}$ coupling of 6.9 Hz, suggesting a $^3J$ coupling. Consequently, the $^{13}C$ NMR signal corresponds to $C^5$ and the proton signal to $H^3$ (see Figure S72 for carbon and proton assignments).

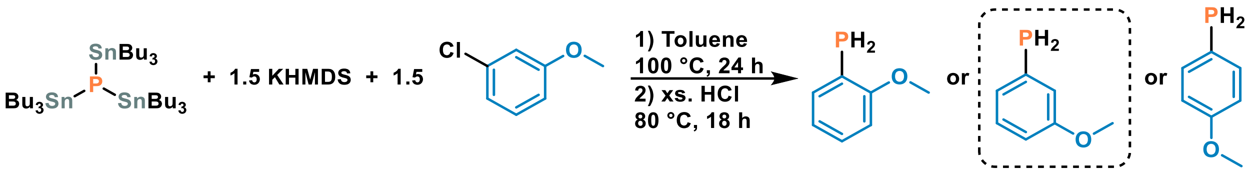


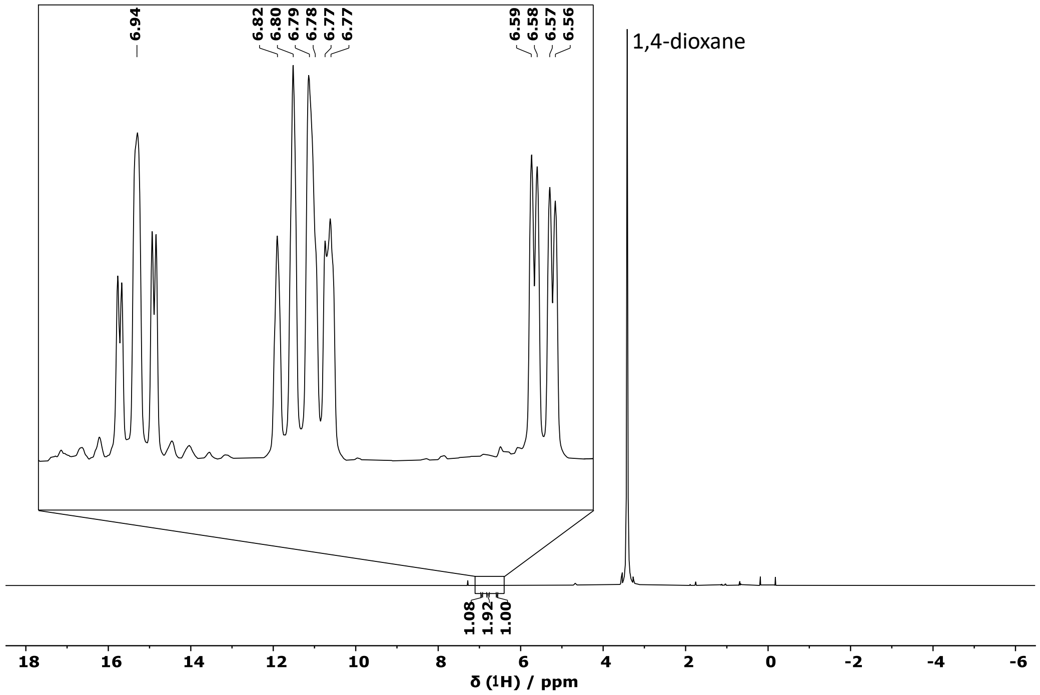


**Figure S68**: $^1$H NMR spectrum of the reaction of $(Bu_3Sn)_3P$ with 3-chloroanisole and KHMDS and subsequent protonation.

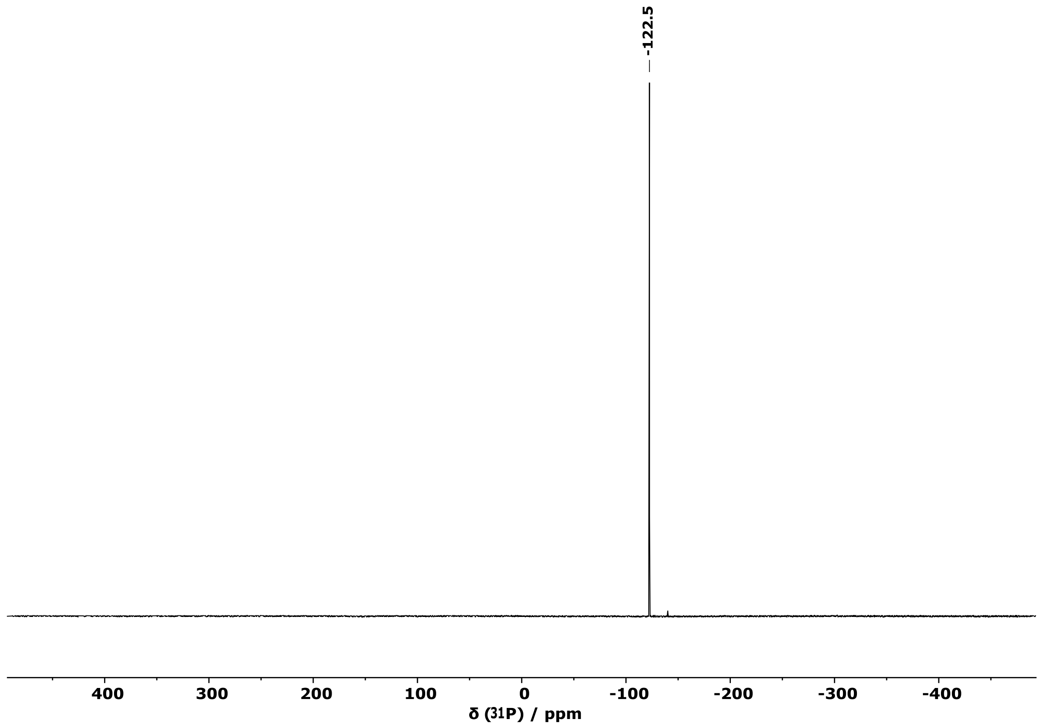


**Figure S69**: $^{31}P\{^{1}H\}$ NMR spectrum of the reaction of $(Bu_3Sn)_3P$ with 3-chloroanisole and KHMDS and subsequent protonation.

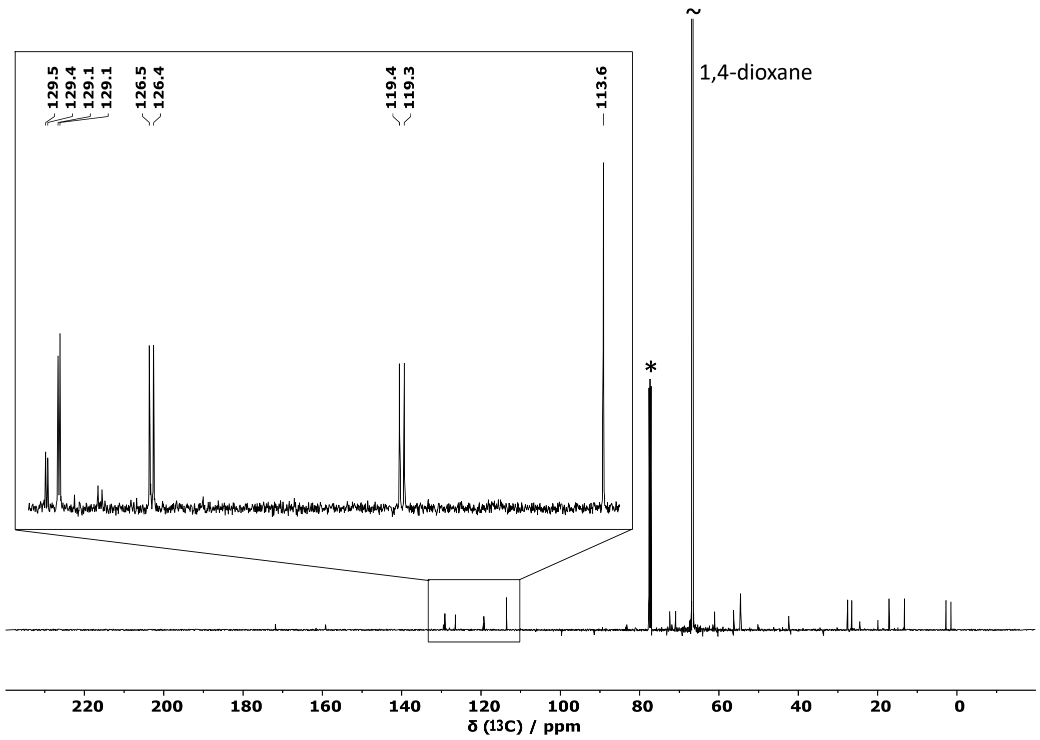


**Figure S70**: $^{13}C\{^{1}H\}$ NMR spectrum of the reaction of $(Bu_3Sn)_3P$ with 3-chloroanisole and KHMDS and subsequent protonation. * marks $CDCl_3$. The signal marked with "~" is truncated for clarity.

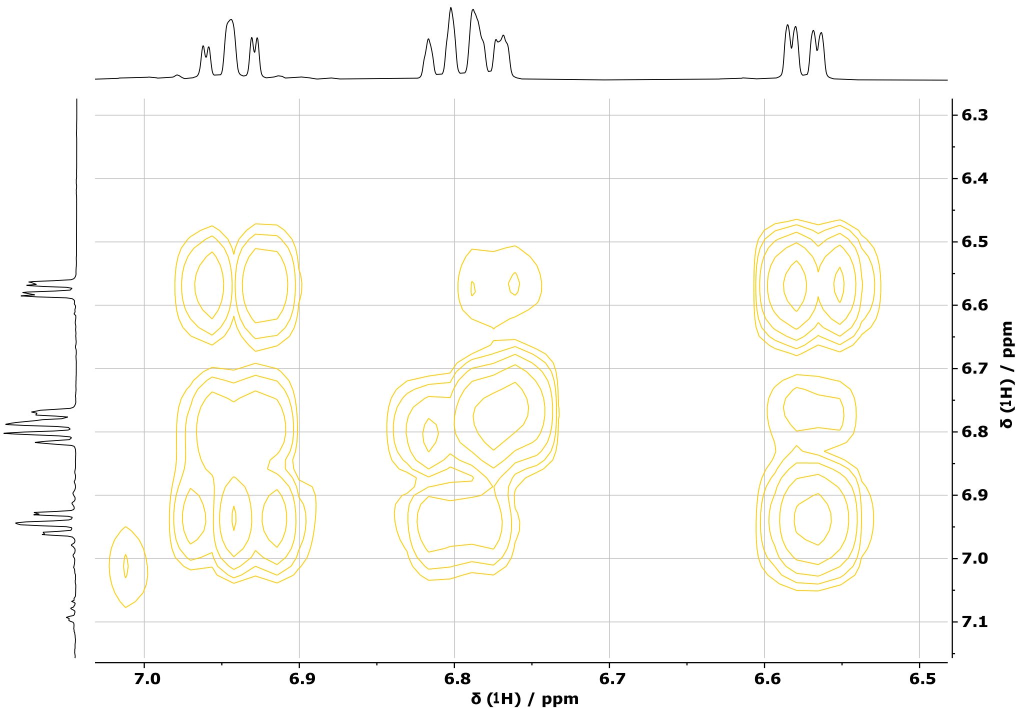


**Figure S71**: $^{1}$H-COSY NMR spectrum of the reaction of $(Bu_3Sn)_3P$ with 3-chloroanisole and KHMDS and subsequent protonation.

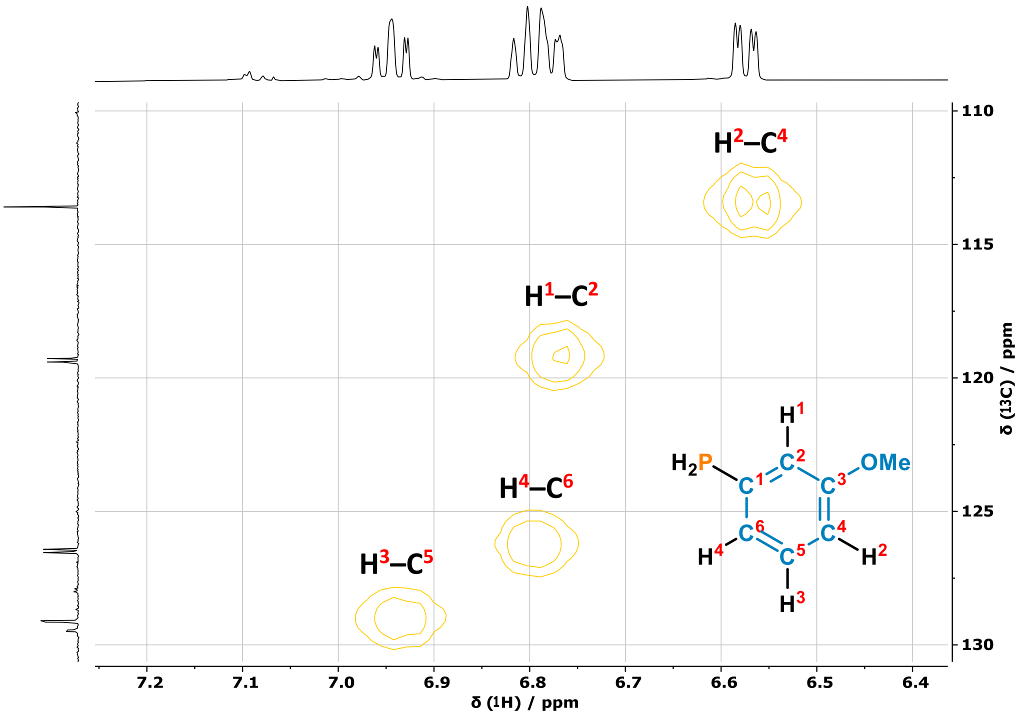


**Figure S72**: $^{1}$H–$^{13}$C HSQC NMR spectrum of the reaction of $(Bu_3Sn)_3P$ with 3-chloroanisole and KHMDS and subsequent protonation, with carbon and proton assignment.

## S9. References


[1] D. J. Scott, J. Cammarata, M. Schimpf, R. Wolf, *Nat. Chem.* **2021**, *13*, 458-464.

[2] I. Kalinina, F. Mathey, *Organometallics* **2006**, *25*, 5031-5034.

[3] M. Méndez, A. Cedillo, *Comput. Theor. Chem.* **2013**, *1011*, 44-56.

[4] M. Pereyre, J.-P. Quintard, A. Rahm, *Tin in Organic Synthesis*, Butterworths, **1987**.

[5] K. Inaba, H. Shiraishi, Y. Soma, *Wat. Res.* **1995**, *29*, 1415-1417.

[6] A. G. Davies, A. Sella. R. Sivasubramaniam, *J. Organomet. Chem.* **2006**, *691*, 3556-3561.

[7] C. A. Russel, N. S. Townsend, *Phosphorus(III) Ligands in Homogeneous Catalysis: Design and Synthesis*, **2012**, *11*, 343-354.

[8] A. V. Lis, I. P. Tsyrendorzhieva, A. I. Albanov, V. I. Rakhlin, M. G. Voronkov, *Russ. J. Org. Chem.* **2013**, *49*, 1451-1453.

[9] T. L. Gilchrist, *Science of Synthesis*, Thieme, **2008**, *43*, 151-224.

[10] I. Bonnaventure, A. B. Charette, *J. Org. Chem*. **2008**, *73*, 6330-6340.
